# Lessons from single cell omics: admixed American ancestry and sex confer cardiometabolic disease risk in Mexicans

**DOI:** 10.1186/s13073-026-01633-x

**Published:** 2026-03-28

**Authors:** Asha Kar, Seung Hyuk T. Lee, Marcus Alvarez, Sandhya Rajkumar, Sankha Subhra Das, Ana Ochoa-Guzmán, Linda Liliana Muñoz-Hernandez, Daniela Mariel Guillen-Quintero, Miguel Herrera-Hernandez, Ivette Cruz-Bautista, Sini Heinonen, Tuure Saarinen, Anne Juuti, Markku Laakso, Kirsi H. Pietiläinen, Brunilda Balliu, Carlos Aguilar-Salinas, Maria Teresa Tusié-Luna, Päivi Pajukanta

**Affiliations:** 1 Department of Human Genetics, David Geffen School of Medicine at UCLA, Los Angeles, CA USA; 2 Bioinformatics Interdepartmental Program, UCLA, Los Angeles, CA USA; 3 Unidad de Biología Molecular y Medicina Genómica, Instituto Nacional de Ciencias Médicas y Nutrición Salvador Zubirán, Mexico City, Mexico; 4 Unidad de Investigación de Enfermedades Metabólicas of the Instituto Nacional de Ciencias Médicas y Nutrición Salvador Zubirán, Mexico City, Mexico; 5 Consejo Nacional de Ciencia y Tecnología, Mexico City, Mexico; 6 Surgery Direction of the Instituto Nacional de Ciencias Médicas y Nutrición Salvador Zubirán, Mexico City, Mexico; 7 Obesity Research Unit, Research Program for Clinical and Molecular Metabolism, Faculty of Medicine, University of Helsinki, Helsinki, Finland; 8 Department of Abdominal Surgery, Abdominal Center, Helsinki University Hospital and University of Helsinki, Helsinki, Finland; 9 Institute of Clinical Medicine, University of Eastern Finland and Kuopio University Hospital, Kuopio, Finland; 10 Healthy Weight Hub, Abdominal Center, Helsinki University Hospital and University of Helsinki, Helsinki, Finland; 11 Department of Pathology and Laboratory Medicine, David Geffen School of Medicine at UCLA, Los Angeles, CA USA; 12 Department of Computational Medicine, David Geffen School of Medicine at UCLA, Los Angeles, CA USA; 13 Department of Biostatistics, UCLA Fielding School of Public Health, Los Angeles, CA USA; 14 Tecnológico de Monterrey, Escuela de Medicina y Ciencias de La Salud, Mexico City, México; 15 Unidad de Biología Molecular y Medicina Genómica, Instituto de Investigaciones Biomédicas UNAM/Instituto Nacional de Ciencias Médicas y Nutrición Salvador Zubirán, Mexico City, Mexico; 16 Institute for Precision Health, David Geffen School of Medicine at UCLA, Los Angeles, CA USA

**Keywords:** Single cell RNA-sequencing, Cardiometabolic diseases, Mexicans, Subcutaneous adipose tissue, Polygenic risk, Colocalization of cell-type level *cis*-eQTL and GWAS signals

## Abstract

**Background:**

Subcutaneous adipose tissue (SAT), the key human fat depot for cardiometabolic health, exhibits high cellular heterogeneity. However, the contributions of contexts and cardiometabolic diseases (CMDs) to this heterogeneity are poorly understood, especially in admixed populations. Despite the substantially increased risk of obesity and obesity-related CMDs in Mexicans, cell-type-level mechanisms behind their elevated CMD risk have remained elusive.

**Methods:**

To investigate how cell-type and subcell-type level profiles of SAT are impacted by sex, admixed American ancestry, CMD traits, and cell-type level *cis* regulation in Mexicans, we generated a Mexican SAT single nucleus RNA sequencing cohort (*n* = 49). We performed cell-type level differential expression testing, weighted gene co-expression analysis, and c*is*-expression quantitative trait locus (eQTL) mappings. We then integrated genome-wide association study (GWAS) and Mexican population level data to assess partitioned polygenic risk for lipid outcomes and colocalization between SAT cell-type level *cis*-eQTL variants and lipid GWAS variants.

**Results:**

First, we discovered and validated a sex-associated adipocyte subtype, overlapping an adipocyte co-expression network with 132 adipocyte function centered genes, including key triglyceride biosynthesis genes, *GPAM*, *DGAT2*, *ACSL1*, and *LPL*, that are differentially expressed (DE) by sex. The *cis* regional variants of these network genes DE by sex confer a significant sex-specific polygenic risk to serum triglycerides, a clinically important atherogenic lipid trait. We also found significant enrichment of progesterone receptor binding at these variant sites, contributing to the sex-specific findings. Second, we identified 34 colocalized genes for three lipid traits, of which 25 (74%) genes have not been identified in previous European colocalization studies using SAT bulk tissue. Among the discovered 25 lipid GWAS genes, 12 are regulated by Mexican enriched and seven by European enriched *cis*-eQTL variants, thus mechanistically elucidating the genetic dyslipidemia susceptibility at the cell-type level in both Europeans and Mexicans. The identified 12 lipid GWAS genes regulated by a Mexican enriched variant include an important adipogenesis gene, *CYP26B1*, and a regulator of adipocyte browning and beiging, *GPR180*.

**Conclusions:**

We identify sex- and ancestry-stratified genes and variants contributing to the risk of adverse cardiometabolic outcomes and improve understanding of the complex cell-type level biological mechanisms underlying CMDs in Mexicans.

**Supplementary Information:**

The online version contains supplementary material available at 10.1186/s13073-026-01633-x.

## Background

White adipose tissue is a lipid-dense connective tissue that plays fundamental roles in energy homeostasis [1]. In humans, the adipose tissue is mainly concentrated in two depots; the subcutaneous adipose depot (SAT), which is positioned beneath the skin, and the deeper visceral depot (VAT), which is concentrated in the omentum and around the stomach organs [1]. SAT, which is the larger, more readily available depot of the two and comprises over 80% of fat present in the human body [2, 3], contributes to many vital processes, including hormonal regulation and insulation of the body [1]. SAT is also a key tissue of interest for cardiometabolic health, both buffering and expanding in the presence of obesity [3]. Thus, especially amid the growing epidemics of obesity-related cardiometabolic diseases (CMDs) [4], it is crucial to obtain a comprehensive understanding of the cell populations forming the SAT depot.

SAT harbors a heterogeneous, dynamic population of cell-types. These include the fat-storing adipocytes, the progenitor and fibroblast-like adipose stem and precursor cells (ASPCs), immune cell-types from both the myeloid and lymphoid lineages, and components of the vasculature, such as pericytes and endothelial cells [5–7]. Although adipocytes typically constitute the largest proportion of cells in SAT, followed by either the progenitor or vascular cell-types [6, 8, 9], the cellular composition of the tissue is prone to fluctuations and heterogeneity. This cellular heterogeneity in SAT has also been linked to cell-type-specific changes in physical characteristics and functional capabilities in SAT [8–13].

Enhanced by the emerging high throughput single cell and nucleus RNA sequencing technologies, which enable transcriptional profiling of SAT at the single cell resolution, distinct alterations in the SAT cell-type level gene expression profiles have been identified as underlying these patterns of cell-type variation [8, 14, 15]. Single cell atlas efforts, which aim to systematically characterize the resident cell-types in each biological system, including SAT, have proposed that these transcriptional diversities may map to subcell-types that are specific to contexts, such as sex, CMDs, or clinical interventions [5, 6, 16–23]. However, the patterns of context and CMD specificity and genetic *cis* regulation have remained largely elusive in SAT cell-types and subcell-types, as challenges in sample size, sequencing depth, and the difficulty to capture the entire SAT population due to the fatty, fragile structure of adipocytes [5, 24] have limited previous studies.

Particularly unexplored is the influence of different contexts and CMDs in admixed populations, since most prior SAT studies comprise largely European cohorts [5, 6, 20, 22, 23]. The Mexican population contains a unique genetic architecture, stemming from a 3-way admixture of European, African, and indigenous American ancestral lineages [25]. Mexicans are also predisposed to substantially increased risks of obesity, type 2 diabetes (T2D) [26–28], and unfavorable serum lipid profiles, including high levels of triglycerides (TGs) and low levels of high-density lipoprotein cholesterol (HDL-C) [26, 29–31]. The previous genome wide association studies (GWASs) have attributed this higher CMD susceptibility to increased allele frequencies of the known risk variants, as well as altered patterns of linkage disequilibrium (LD) due to the admixture and the presence of novel, still unknown population-specific risk variants [32–34]. Yet, due to the current lack of Mexican single cell level omics studies of CMDs, there is a wide knowledge gap regarding how these CMD risk variants affect biological function in cell-types of key CMD tissues in Mexicans.

To address this medical knowledge gap, we generated a Mexican SAT single nucleus RNA sequencing (snRNA-seq) cohort, and conducted single cell based genetic risk assessments of lipid traits in an independent Mexican GWAS cohort. Analyzing the SAT single cell level transcriptomics data of the cohort, we studied the tissue at the cellular and subcell-type resolutions, discerned the cell-type level co-expression networks, surveyed the cell-type level *cis* regulatory landscape of SAT, and linked these data to contexts (e.g., sex and admixed American ancestry) and CMD traits. First, our work resulted in the discovery of large sex-specific effects on adipocyte genes contributing to CMDs. We identified a sex-associated adipocyte subtype and its corresponding adipocyte co-expression network with genes differentially expressed (DE) by sex. We then showed that regional variants of these DE genes also confer a sex-dependent polygenic risk of serum TGs in Mexicans using cell-type level network polygenic risk scores (PRSs). Second, we uncovered colocalized genes *cis* regulated at the cell-type level by cardiometabolic GWAS risk variants with large allele frequency differences between populations, thus elucidating the role of ancestry in SAT cell-type level *cis* regulatory signals underlying CMDs. To summarize, we not only created a large Mexican single cell level SAT reference but also discovered new biology by revealing previously unknown sex- and ancestry-specific genes and variants underlying SAT interindividual heterogeneity and the genetic risk of unfavorable serum lipid levels in this admixed population with a high CMD risk.

## Methods

### Study cohorts

#### Mexican subcutaneous adipose tissue snRNA-seq cohort

The Mexican subcutaneous adipose tissue snRNA-seq cohort comprises 49 participants, recruited in the Instituto Nacional de Ciencias Médicas y Nutrición Salvador Zubirán, Mexico City. The mean age of the cohort is 45.0 years (SD = 11.9 years), with 37 (75.5%) females. The mean body mass index (BMI) is 30.0 kg/m^2^ (SD = 7.6 kg/m^2^), where 14 (28.6%) individuals have normal BMI, 16 (32.7%) individuals have overweight BMI, and 19 (38.8%) are obese (Additional file 1: Table S1). Fasting serum lipid levels (triglycerides, HDL-C, and total cholesterol (TC)) were measured using standardized methods, as described previously [35]. The study was approved by the Ethics Committee of the Instituto Nacional de Ciencias Médicas y Nutrición Salvador Zubirán, Mexico City, and all participants gave written informed consent. All research conformed to the principles of the Declaration of Helsinki.

#### Mexican Metabolic (MetMex) cohort for PRS studies

The MetMex cohort (*n* = 8,375 unrelated individuals with both genotype and phenotype data available for study), was previously recruited in the Instituto Nacional de Ciencias Médicas y Nutrición Salvador Zubirán, Mexico City [35]. Fasting serum lipid levels (TGs, HDL-C, and TC) were measured using standardized methods [35]. MetMex participants have been genotyped using the Illumina Global array (see below). The MetMex study was approved by the Ethics Committee of the Instituto Nacional de Ciencias Médicas y Nutrición Salvador Zubirán, Mexico City, and all participants gave written informed consent. All research conformed to the principles of the Declaration of Helsinki.

#### RYSA SAT snRNA-seq replication cohort

The Roux-en-Y versus one-anastomosis gastric bypass (RYSA) bariatric surgery cohort comprises Finnish individuals with obesity, recruited at the Helsinki University Hospital, Helsinki, Finland, as described in detail previously [36]. To verify and replicate our subtype, network, DE gene, and *cis*-eQTL results, we used existing SAT snRNA-seq and genotype data, generated using the Infinium Global Screening Array-24 v1, from 68 individuals in the RYSA cohort [37]. The study was approved by the Helsinki University Hospital Ethics Committee, Helsinki, Finland, and all participants gave written informed consent. All research conformed to the principles of the Declaration of Helsinki.

#### METabolic Syndrome In Men (METSIM) cohort

We used existing genotype data from the METSIM cohort (*n* = 8,254), which comprises Finnish males who were originally recruited at the University of Eastern Finland and Kuopio University Hospital, Kuopio, Finland [38, 39]. The METSIM study was approved by the Ethics Committee of the Northern Savo Hospital District, and all participants provided written informed consent. All research conformed to the principles of the Declaration of Helsinki.

#### Mexican Obesity Study (MOSS) bulk RNA-sequencing cohort

The Mexican Obesity Study (MOSS) cohort consists of individuals recruited in the Instituto Nacional de Ciencias Médicas y Nutrición Salvador Zubirán. The participants underwent bariatric surgery and participated in a 1-year follow-up, as described in detail previously [13, 40]. In this study, we used previously generated bulk RNA-sequencing (bulk RNA-seq) data collected from 45 participants at both the operation and 1-year follow-up time-points [13, 40]. The MOSS study was approved by the Ethics Committee of the Instituto Nacional de Ciencias Médicas y Nutrición Salvador Zubirán, Mexico City, and all participants provided written informed consent. All research conformed to the principles of the Declaration of Helsinki.

#### UK Biobank (UKB) cohort

The UKB cohort comprises over 500,000 individuals from the general population of the United Kingdom, originally recruited through the UK National Health Service between 2006 and 2010 [41, 42]. In this study, we used existing genotype and phenotype data from 373,483 unrelated individuals of European background from UKB to extend our polygenic risk assessments. The UK Biobank has approval from the North West Multi-centre Research Ethics Committee (MREC), and all participants provided written informed consent. All research conformed to the principles of the Declaration of Helsinki. Data from UKB were accessed under application number 33934.

#### Definition of the traits in the Mexican SAT snRNA-seq cohort

**Fig. 1:**
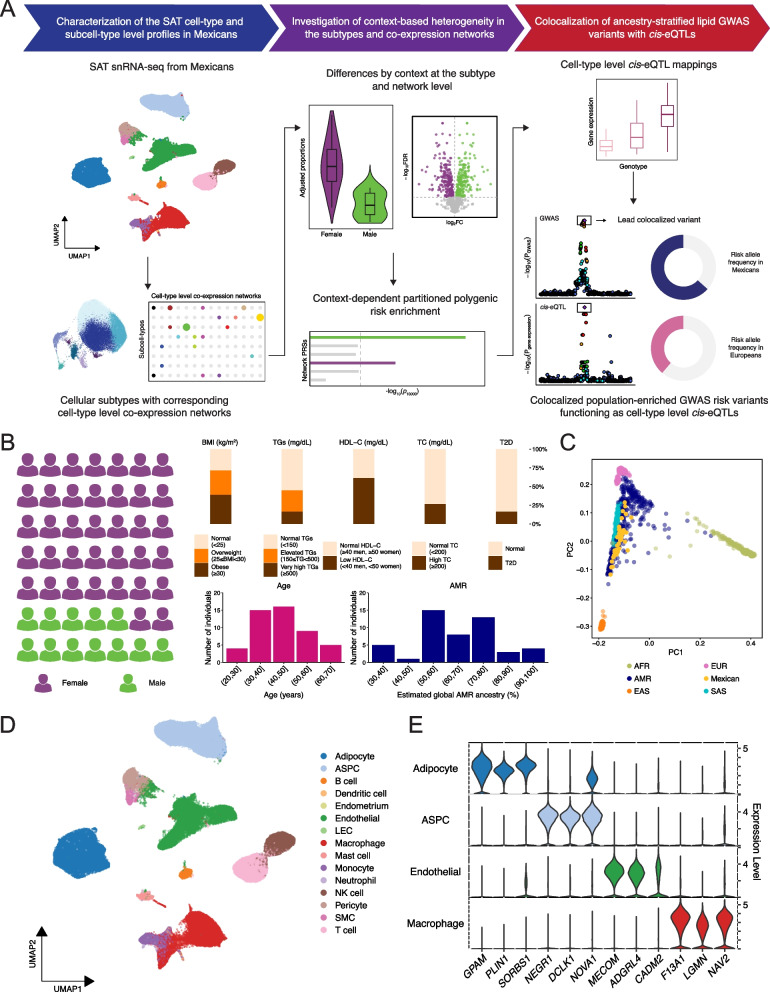
Subcutaneous adipose tissue (SAT) single nucleus RNA-sequencing of Mexican cohort with widespread age range, ancestral admixture, and BMI categories captures the different SAT cell-type populations and their key transcriptional signatures. **A** Schematic overview of the study design that discovers sex- and ancestry-stratified genes and variants contributing to risk profiles of adverse cardiometabolic outcomes in Mexicans. **B** Graphical representations of the phenotype distributions in the Mexican cohort by the context of sex, global admixed American (AMR) ancestry, and age, and the CMD traits of body mass index (BMI), type 2 diabetes (T2D), serum triglycerides (TGs), serum high-density lipoprotein cholesterol (HDL-C), and serum total cholesterol (TC) (for definition of these traits, see Methods). **C** The genotype data from the Mexican SAT single nucleus RNA-sequencing (snRNA-seq) cohort (*n* = 49) are projected onto the 1000 Genomes [46] principal component space using FlashPCA [47]. Each dot represents an individual, colored by the population of origin, i.e., one of the superpopulations defined in the 1000 Genomes Project, or Mexican for those in the present study. The admixed American super population is shown as AMR, colored dark blue; European EUR, pink; East Asian EAS, orange; South Asian SAS, teal; African AFR, green; and the Mexican SAT snRNA-seq cohort, yellow. **D** Uniform manifold approximation and projection (UMAP) visualization of the 128,057 nuclei obtained from the SAT biopsies of the 49 Mexican participants, colored by assigned cell-type. **E** Violin plots show the expression in adipocytes, ASPCs, macrophages, and endothelial cells of the top three unique cell-type marker genes by the log_2_-fold change per cell-type. (D-E) ASPC indicates adipose stem and precursor cells, LEC lymphatic endothelial cells, and SMC smooth muscle cells

We defined the traits for the three contexts (age, sex, and estimated global admixed American (AMR) ancestry proportion, and five CMD traits (T2D, BMI, fasting serum TC, HDL-C, and TGs) as follows: lower age (age ≤ median of 47 years) and higher age (age > 47); males and females; T2D cases and controls [35, 43]; lower global AMR ancestry (global AMR proportion ≤ median of 0.624) and higher global AMR ancestry (global AMR proportion > 0.624), estimated using ADMIXTURE [44] (see below); normal BMI (BMI < 25 kg/m^2^), overweight (25 ≤ BMI < 30), and obese (BMI ≥ 30); normal TC (TC < 200 mg/dL) and high TC (TC ≥ 200); low HDL-C (HDL-C < 40 mg/dL for males, < 50 mg/dL for females) and normal HDL-C; normal TGs (TGs < 150 mg/dL), elevated TGs (150 ≤ TGs < 500), and very high TGs (TGs ≥ 500), respectively (Fig. 1B; Additional file 1: Table S1). The used lipid trait cut-points are based on the American Heart Association guidelines [27, 45]. For pseudobulk DE and heterogeneity assessments, continuous values were used for quantitative outcomes (age, global AMR ancestry, BMI, TC, HDL-C, and TGs). For the comparisons of cell-type and subcluster proportions, we binarized BMI into non-obese (BMI < 30) and obese (BMI ≥ 30), and TGs as normal TGs (TGs < 150 mg/dL) and high TGs (TGs ≥ 150).

#### Genotyping the Mexican SAT snRNA-seq and MetMex cohorts

Both the Mexican snRNA-seq cohort and the MetMex cohort were genotyped using the Infinium Global Screening Array-24 v1 (Illumina). The genotype data from each cohort were quality controlled (QCed) using PLINK v1.9 [48]. We first excluded the individuals with genotype data missingness > 5% and cross-checked each individual’s reported sex with the inferred biological sex imputed using the ‘–sex-check’ function in PLINK v1.9 [48]. We included unrelated individuals, defined by KING v2.3.2 [49] with ‘–unrelated –degree 2’. We additionally checked for excess heterozygosity by excluding individuals with heterozygosity ± 3 × standard deviation (SD) from the mean and removed duplicate individuals. Next, we excluded unmapped and strand ambiguous single nucleotide polymorphisms (SNPs), monomorphic SNPs, and variants with missingness > 5%, and Hardy–Weinberg Equilibrium (HWE) *p*-value < 10^−12^. All remaining high-quality genotypes in PLINK format from the individuals in the Mexican snRNA-seq and MetMex cohorts were then converted to VCF format for the imputation.

We performed genotype imputation for both cohorts separately on the TOPMed imputation server against the TOPMed reference panel version r2 [50] after excluding duplicate SNPs and SNPs that did not match with the reference panel. Any strand flips or allele switches based on the reference panel were performed by the server, and the haplotype phasing and imputation were performed using Eagle v2.4 [51] and minimac4 [52], respectively. We filtered out imputed SNPs by imputation score, Rsq metric from minimac4 < 0.6, minor allele frequency (MAF) < 1%, and HWE *p*-value < 10^−12^ for the downstream analyses.

### Global ancestry estimation

We estimated the global ancestry proportions separately in the Mexican snRNA-seq cohort using ADMIXTURE [44], similarly as previously [53]. We first limited the QC-passing genotype data to SNPs with only an A, C, G, or T allele and MAF > 0.05, and then merged the data with the 1000 Genomes Phase 3 dataset [46] by the common SNPs. Next, the merged datasets were LD pruned every 2 kb (‘–bp-space 2000’) using PLINK v1.9 [48]. We used the LD pruned set of SNPs and the unsupervised clustering mode of ADMIXTURE with the number of clusters k = 4 to estimate the global ancestry proportions. We annotated each cluster based on the proportions in the 1000 Genomes Phase 3 dataset [46] where the cluster which was most abundant for the admixed American superpopulation was labeled as AMR, for the African superpopulation as African, for the East Asian superpopulation as East Asian, and for the European superpopulation as European ancestry (Additional file 2: Fig. S1; Additional file 1: Table S2).

### Bulk RNA-seq preprocessing

We aligned the previously generated SAT bulk RNA-seq reads from the MOSS cohort [13, 40] to the GRCh38 human reference genome with GENCODE v42 annotations [54] using STAR v.2.7.10b [55] in a two-pass mode. Retaining only uniquely mapped reads, we obtained the technical metrics from Picard Tools v2.25.0 (http://broadinstitute.github.io/picard) and gene-name level counts using featureCounts v2.0.6 [56].

### Nuclei isolation, snRNA-seq library construction, and sequencing

We performed snRNA-seq experiments on open abdominal subcutaneous adipose biopsies obtained from 49 individuals of the Mexican adipose biopsy cohort [57, 58], as previously described with minor modifications [40]. We isolated nuclei from frozen subcutaneous adipose tissue from individuals independently or by pooling biopsy samples, resulting in 15 singletons and 10 pools, QCed as detailed below. We determined the isolated nuclei quality and measured concentration using Countess II FL Automated Cell Counter after staining the nuclei with trypan blue and Hoechst stain. The nuclei were then immediately processed using the Single Cell 3’ Reagent Kit v3.1(10 × Genomics) for the library construction. We sequenced the libraries on an Illumina NovaSeq S4 with a target sequencing depth of 60,000 reads per nucleus.

### Read alignment and gene quantification of SAT snRNA-seq data

We aligned the snRNA-seq reads against the GRCh38 reference genome and obtained gene level counts with GENCODE v42 [54] annotations using STARSolo v2.7.10b [55, 59] with the following parameters: soloType CB_UMI_Simple, soloUMIlen 12, soloUMIdedup 1MM_CR, soloUMIfiltering MultiGeneUMI_CR, soloCBmatchWLtype 1MM_multi_Nbase_pseudocounts, soloStrand Forward, clipAdapterType CellRanger4, soloFeatures Gene GeneFull SJ Velocyto GeneFull_ExonOverIntron GeneFull_Ex50pAS, alignSJoverhangMin 8, alignSJDBoverhangMin 3, alignIntronMin 20, alignIntronMax 1,000,000, soloCellReadStats Standard, outSAMattributes NH HI nM AS CR UR CB UB GX GN sS sQ sM, outFilterScoreMin 30.

### Removing contaminated and low-quality snRNA-seq data

For the snRNA-seq data, we first applied cluster-based DIEM [60] filtration to remove reads from extranuclear RNA using a unique molecular identifier (UMI) cutoff of 500 for debris droplets and cluster initialization size of 50. We manually identified and removed ambient RNA clusters, defined as clusters with lower UMI and gene counts than the remaining clusters, high presence of mitochondrial genes among the top cluster expressed genes, and high mitochondrial read percentage. We also removed low quality reads, retaining only the droplets with over 500 total UMIs, 200 genes with non-zero expression, mitochondrial read percentage below 10%, and spliced read fraction below 0.75.

After DIEM, we used Seurat v4 [61] to cluster the data. We log-normalized and scaled the data and computed principal components (PCs) with the top 2,000 variable features, excluding mitochondrial and ribosomal genes. We then applied a Uniform Manifold Approximation and Projection (UMAP) dimensional reduction and identified clusters using the Louvain-based approach implemented by Seurat [61] with 30 PCs and a resolution of 0.5. To further account for contamination within each of the nuclei, we performed read decontamination with decontX [62], using the previously computed clusters as the prior for broad cell-type identity and all aligned reads with below 500 UMIs as the background ambient profile. As decontX [62] directly corrects gene expression counts, we removed contaminated reads by recomputing the UMI counts, feature counts, and mitochondrial read percentage, and again only keeping droplets with over 500 and below 30,000 total UMIs, 200 genes with non-zero expression, and mitochondrial read percentage below 10%.

### Demultiplexing of pooled samples to individuals of origin

We used the demuxlet [63] tool to demultiplex the DIEM-cleaned data of the pooled samples back to their original individual identities based on their imputed DNA-based genotype. Doublets identified by demuxlet [63] were removed from the decontX [62] cleaned data of the sample pools.

### Doublet estimation and removal

To remove heterotrophic doublets, we first re-clustered each sample using Seurat [61]. We then identified heterotypic doublets by using the doubletFinder R package [64] with 30 PCs. As recommend by McGinnis et al. [64], we determined the pK and expected doublet rate on a per sample basis due to the transcriptional similarities between many of the adipose cell-types. We observed that a bimodal distribution for the proportion of artificial nearest neighbors (pANN) biased the assignment of doublets towards adipocytes. Thus, for each of the singleton samples and sample pools, we empirically selected the pK values with optimal or near optimal mean–variance-normalized bimodality coefficient and the expected doublet rate based on the calculated pANN distribution for the selected pK. After removing doublet nuclei identified by doubletFinder [64], we recalculated the clusters for each sample with Seurat [61].

### Cell-type annotation of quality-controlled snRNA-seq data

We used SingleR [65] to assign the cell-type identity for each individual nucleus, providing the annotated single cell level data from SAT by Emont et al. [5, 66] as the reference. We removed the droplets labeled as NA, indicating that their cell-type could not be determined, resulting in 49 samples and 128,057 QC passing nuclei. We included a summary of the cell-type prediction confidence scores in Additional file 3: Table S3 and noticed that these clean nuclei distributed across the samples and batches (Additional file 2: Fig. S2) in a highly similar way as shown in previous papers of single cell level data in solid tissues [67, 68].

### Clustering and integration of quality-controlled snRNA-seq data

We combined and clustered samples together with Seurat [61]. We filtered the genes to only the expressed genes, considered a gene “expressed” if it had counts of greater than or equal to three in at least three nuclei [62]. To integrate the data, we recomputed the PCs and used Harmony [69] to obtain batch-corrected embeddings. Finally, clusters were called by Seurat [61] using a resolution of 0.5 and 30 Harmony [69] components.

### Subclustering of snRNA-seq data into subtypes

To subcluster the data and identify cellular subtypes, we first annotated each of the clusters to one of five broad cell-type categories both by using SingleR [65] with broad annotations from the SAT reference snRNA-seq data [5, 66] and checking the known canonical marker expression [5] for adipocytes, myeloid cells, lymphoid cells, vascular cells, and ASPCs, omitting the few nuclei labelled as endometrium. We split the data into five separate objects, one per broad cell-type, and recomputed the Harmony [69] components. We re-clustered the data, where both the number of Harmony [69] components and clustering resolution were selected separately for each broad cell-type based on the marker gene profiles, cluster annotations with SingleR [65], and cluster stability. To manually assign subtype labels to each cluster, we integrated multiple lines of evidence, including the expression of canonical marker genes previously reported by earlier literature [5, 6, 16–19, 24, 70, 71], the overall marker gene signatures per cluster, and the SingleR subtype annotations from the previous adipose atlas reference [5, 66]. For cell-types with multiple clusters, we numbered them by cluster size, such that the largest cluster was assigned as subtype 1. Finally, we verified that all assigned subtypes had unique subtype marker genes within the broad cell-type and were not specific to a single individual.

To compare and harmonize our subtype annotations against the previously published SAT atlases by Miranda et al. [22, 72], Lazarescu et al. [21, 73], and Emont et al. [5, 66], we obtained their publicly available subtype-annotated human SAT single cell gene expression data files and split the data into five broad cell-type objects for adipocytes, ASPCs, vascular cells, lymphoid, and myeloid cell-types. In each reference broad cell-type data object, we computed the Harmony [69] embeddings, as described above, and used Seurat [61] to project the reference Harmony embeddings to the gene expression data of the corresponding broad cell-type in our study. We then annotated our data with the reference subtype labels based on the shared nearest neighbor space of 30 Harmony components.

### Identification of cell-type marker genes

We determined the marker genes for each cell-type and subtype using the Wilcoxon rank sum test implemented by the FindAllMarkers function in Seurat [61], with logFC.threshold = 0.25 and min.pct = 0.25, as described previously [74, 75]. To obtain unique marker genes, we removed the genes identified as marker genes for more than one cell-type or subtype.

### Pathway enrichments of unique marker genes

We examined our unique cell-type and subtype marker gene sets for enrichment of KEGG pathways and Gene Ontology (GO) biological processes, cellular components, and molecular function relative to a background of all expressed genes in the cell-type using WebGestalt [76].

### Comparisons of cell-type and subtype proportions by CMDs and context

We computed raw cell-type and subtype proportions for each individual using the nuclei counts per cell-type per individual. To compare the proportions of cell-types and subtypes by contexts and CMDs, we adjusted the raw proportions for global AMR ancestry, age, BMI, and sex (Additional file 2: Fig. S3), excluding each of these variables from the corrections when it was the tested outcome, and partitioned the cohort as defined in our marker gene assessments. Wilcoxon tests were performed to determine significant differences (Wilcoxon *p*-value < 0.05) in proportions within each subgroup per context. We also assessed the Spearman’s correlation between the adjusted proportions and continuous variables of age, BMI, global AMR ancestry, and lipid outcomes, defining significant correlations as Spearman’s correlation *p*-value < 0.05.

### Transcriptional heterogeneity assessments with CNA

To quantitatively assess global transcriptional heterogeneity, we used the covarying neighborhood analysis (CNA) tool [77]. We performed global association tests by sex, T2D status, and the continuous outcomes of global AMR ancestry, age, BMI, TC, HDL-C, TGs in the overall snRNA-seq data and each of the main cell-type. We applied an inverse-normal transformation to the outcomes of BMI, TGs, HDL-C, and TC prior to testing, and included age, global AMR ancestry, BMI, sex, and the number of nuclei of the tested data as covariates (Additional file 2: Fig. S3).

### Unsupervised multi-cellular factor analysis to capture system-level transcriptional programs across cell-types

We used the MOFAcellulaR tool to perform an unsupervised multi-cellular factor analysis (MOFAcell) [78] to capture transcriptomic variability across the four main SAT cell-types (adipocytes, ASPC, endothelial cells, and macrophages), as previously described with minor modifications [23]. Briefly, for each of the cell-types, we first aggregated the UMI level gene expression data into pseudobulk expression by summing up all counts per gene per individual from droplets identified as the cell-type. Lowly expressed genes, defined as genes with < 10 counts per individual or detected in < 25% of all individuals [23], were removed from the expression data and Trimmed Mean of M-values (TMM)-normalization was performed. We then trained a model to capture the variability in the data through six latent factors [23] using the highly variable genes in each cell-type. From them, we excluded the cell-type marker genes that may contribute background noise to other cell-types. Lastly, we adjusted the data for the number of nuclei to control for technical confounding.

To assess associations between the latent factors and continuous outcomes of global AMR ancestry, age, BMI, TC, HDL-C, TGs, and categorical outcomes of sex and the T2D status, we fit linear models between the factor scores and each continuous outcome and conducted analyses of variance (ANOVAs) for the categorical outcomes. The continuous outcomes of BMI, TGs, HDL-C, and TC were normalized using rank-based inverse-normal transformations, and *p*-values were adjusted for multiple testing using the Benjamini-Hochberg (FDR) approach, defining significance as FDR < 0.05.

### Testing of expressed genes in each cell-type for differential expression

To test the genes expressed in each cell-type for DE by estimated global AMR ancestry, age, sex, BMI, TC, HDL-C, TGs, and the T2D status, and the adipocyte subtype marker and network genes for DE by sex, we employed the limma R package with the voom [79] normalization method, using continuous values for the quantitative traits. Using the pseudobulk expression data of each cell-type as described above, we retained only cell-type expressed genes, defined as having transcripts per million (TPM) greater than 0.1, at least six raw counts in over 20% of the individuals [80], and occupying the top 90% of reads in the single cell data. We TMM-normalized the pseudobulk counts, and then for each tested outcome, estimated its effects on gene expression, where we included age, global AMR ancestry, BMI, sex, and the number of nuclei of the tested cell-type per individual as covariates (Additional file 2: Fig. S3). We also applied rank-based inverse-normal transformations to the continuous outcomes of BMI, TGs, HDL-C, and TC. After fitting the described model, DE genes, identified as genes with an FDR-adjusted *p*-value < 0.1, were assessed for functional enrichment for WebGestalt [76], similarly as done for the full gene sets.

### Testing DE and sex-associated MOFAcell factor genes for enrichment in subtypes

To evaluate whether adipocyte genes DE by sex are enriched within specific adipocyte subtypes, we performed Fisher’s exact tests to assess the enrichment of their overlap with the unique marker genes of each adipocyte subtype. We separately examined the overlap of the DE genes upregulated in females and upregulated in males. Additionally, we tested whether the subtype marker genes were enriched among the genes contributing to sex-associated MOFAcell [78] factors using gene set enrichment analysis implemented in decoupleR [81], with genes weighted by their loadings for the corresponding MOFAcell factor. Significant enrichment was defined as Bonferroni-adjusted *p*-value (*p*_adj_) < 0.05.

### Single cell level weighted gene co-expression network analysis

To search for cell-type level networks of co-expressed genes and their differential co-expression by the tested phenotypic traits, we performed Weighted Gene Co-expression Network analysis (WGCNA) at the single cell level for all cell-type expressed genes in each of the broad cell-types using hdWGCNA [82, 83]. Briefly, with the separated data per broad cell-type, we grouped nuclei from each sample into metacells with k = 25 (default), max_shared = 10, and the Harmony embeddings. We then used the metacell expression data to compute the optimal soft power threshold and construct a co-expression network. In the constructed network, we derived the harmonized module eigengenes using our single cell expression data, where we harmonized per individual sample identity, and the eigengene-based connectivity of each gene. We conducted Fisher’s exact tests to assess each network for enrichment in overlap with the unique marker genes of each subcluster (FDR < 0.05).

To assess the preservation of the networks in the external SAT snRNA-seq data by Miranda et al. (separately by condition, i.e., lean, obese, and weight loss) [22, 72], in the data by Lazarescu et al. [21, 73], in the data by Emont et al. [5, 66], and in the Finnish RYSA SAT snRNA-seq data [37, 84], we projected the co-expression networks onto the snRNA-seq data for the corresponding broad cell-type of each cohort with the ProjectModules function from hdWGCNA. The ModulePreservation function was used to determine preservation of each network based on the single cell level expression data in the external cohort. We considered networks with Z_summary_ > 10 as highly preserved networks and Z_summary_ > 2 as moderately preserved, as suggested previously [85].

We also compared the first PC of pseudobulk expression in adipocytes of the Adipocyte network VIII genes by sex using a Wilcoxon test. Prior to running PCA, the adipocyte pseudobulk expression of the network genes were TMM and log-normalized, and corrected for age, global AMR ancestry, BMI, and the number of adipocyte nuclei. We then applied rank-based inverse-normal transformations to the computed PCs, and multiplied PC1 by –1 if it was negatively correlated with the average expression of the network, such that PC1 would positively correlate with the overall network gene expression and thus accurately reflect the overall directionalities of the network gene expression.

### Replications of subtype proportion and network eigengene differences by sex and DE genes by sex in the independent RYSA cohort

We used existing SAT snRNA-seq data (*n* = 68) from the independent RYSA cohort [37, 84] to test the observed sex differences in adipocyte subtype proportions and network eigengene values as well as the DE genes by sex for replication. After subsetting the SAT data of the RYSA cohort to only the adipocyte nuclei and recomputing the Harmony embeddings, we transferred the Mexican adipocyte subtype labels to the RYSA data with Seurat [61] using the shared nearest neighbor space of 30 Harmony components to determine anchors for the projection. We then assessed the subtype proportions and preserved cell-type level networks for sex differences in the same way as was done in the Mexican data, but omitting ancestry from the covariates due to the homogeneity of Finns [86]. In these subtype and network analyses in RYSA, we considered the proportion and module eigengenes with differences passing Wilcoxon *p*-value < 0.05 and in same directions as the Mexican cohort to be replicated. We also tested the subtype marker and adipocyte co-expression network genes which showed DE by sex in the Mexican data for DE by sex in RYSA, similarly employing the covariates of age, BMI, and number of nuclei, and considering genes with FDR < 0.1 and consistent DE direction with the discovery cohort as replicated.

### Construction of network polygenic risk scores in the MetMex cohort

We constructed polygenic risk scores (PRSs) for log(TGs), HDL-C and TC for the individuals of the MetMex cohort (*n* = 8,375) using the set based clumping method implemented in PRSice-2 [87]. We used the METAL summary statistics of the trans-ancestry meta-analyses of each lipid outcome published by the Global Lipids Genetics Consortium (GLGC) [88] as the base data, and the quality-controlled genotype data from the MetMex cohort as the target data. We used PRset [89], implemented in PRSice-2 [87], to build network PRSs, comprising only variants landing within the *cis* regions (± 500 kb from ends of gene body) of the genes with replicated DE by sex in adipocyte subtype Adip2 and Adipocyte network VIII. We computed the variance explained (R^2^) by each PRSs for their PRS outcomes in a linear model, where we inverse-normal transformed the outcome and included age, age^2^, sex, BMI, and 20 genetic PCs as covariates (Additional file 2: Fig. S3). We accounted for the use of cholesterol lowering medications by dividing the TC value by 0.8 [88] for those on such medications, prior to the normalization. To assess each of the network PRSs for enrichment in signal, we calculated their competitive *p*-values or rankings of the observed R^2^ relative to the R^2^ of 10,000 random PRSs, each built from a randomly selected independent set of variants of same size from gene regions of cell-type expressed genes, using PRSet [89]. We then constructed each of the network PRSs separately in the males (*n* = 2,893) and females (*n* = 5,482) from the MetMex cohort. Similarly as was done in all individuals, we employed PRSet [87, 89] to build the PRSs and assess for enriched variance explained, but instead using the sex-stratified GWAS summary statistics from GLGC [88] as the base data and only including age, age^2^, BMI, and top 20 genetic PCs as covariates in the regressions.

### Targeted genotype by sex interaction analysis in MetMex

To evaluate the TG GWAS variants in the *cis* regions of the adipocyte network VIII genes DE by sex for interactions with sex on TGs, we fit linear models regressing TG levels against the variant genotype, sex, and interaction between genotype and sex. We limited our analysis to sex-specific TG GWAS variants, i.e., variants passing *p* < 5 × 10^−8^ in either one of the sex-stratified TG GWAS summary statistics but not both. We included age, age^2^, BMI, and 20 genetic PCs as covariates, applied a rank-based inverse-normal transformation to the outcome, and adjusted *p-*values for multiple testing using FDR, with FDR < 0.1 as the significance threshold.

### Network polygenic risk assessment in UKB

To extend our partitioned network TG PRS analysis to UKB, we first conducted GWASs for TGs in half of the cohort, stratified by males (*n* = 85,793), females (*n* = 100,741), and the combined sample (*n* = 186,534). GWASs were performed using BOLT-LMM [90], adjusting for the top 20 genetic PCs, testing center, genotyping array, age, age^2^, BMI, and sex in the sex-combined analysis and applying a rank-based inverse-normal transformation to the outcome. We then constructed partitioned PRSs in the remaining half of the cohort using these UKB GWAS results as base weights. After applying a stringent QC procedure to the genotype data as previously described [8, 91], partitioned PRSs were generated and assessed for R^2^ enrichment relative to 10,000 permutations with PRSet [87, 89], using the network PRS and background variant sets from MetMex.

To test the partitioned network TG PRS for an interaction sex on TGs in UKB, we also performed a sex-combined TG GWAS in the UKB base group, in which we did not adjust for sex. We built the sex-combined network TG PRS with PRSet [87, 89], using the non-sex-adjusted TG GWAS for the variant level weights. Next, we linearly modeled the normalized TG values against this PRS, sex, their interaction, and the covariates (top 20 genetic PCs, testing center, genotyping array, age, age^2^, and BMI) and assessed the significance of the interaction coefficient.

### F_ST_ analysis of network TG PRS variants between Mexicans and Europeans

We computed overall and SNP-wise fixation index (F_ST_) values between Mexican and European men for the male network TG PRS using the Weir and Cockerham approach [92]. We calculated F_ST_ between the unrelated Mexican men in the MetMex cohort and an equal number of randomly selected unrelated European men from UKB [41, 42], as well as between the unrelated Mexican men and an equal number of randomly selected unrelated Finnish men from the all-male METSIM cohort [38]. To assess enrichment, we compared the overall F_ST_ of these variants to the distribution of overall F_ST_ values from 10,000 randomly selected, size-matched sets of variants from the PRS background, i.e., independent variants in the *cis* regions of all adipocyte expressed genes.

### Transcription factor motif enrichment analysis

We tested the independent clumped variants that we used in PRSet to construct the male-specific partitioned network TG PRS for an enrichment of transcription factor (TF) binding motifs using HOMER [93] with a window size of 100 bp.

### Cell-type level cis-eQTL mapping

We identified *cis*-expression quantitative trait loci (eQTLs) at the cell-type level by conducting eQTL mappings on the cell-type level pseudobulk data. We only tested the four most abundant cell-types to ensure sufficient numbers of nuclei and expression data in all samples. After aggregating the UMI level expression into pseudobulk, we converted the pseudobulk counts into TMM-normalized counts per million (CPMs) within each tested cell-type. We filtered for genes with both TPM greater than 0.1 and at least six raw counts in over 20% of the individuals [80] and performed rank-based inverse-normal transformations. To detect cell-type level *cis*-eQTLs, we used the Matrix eQTL R package [94], testing all variants with MAF > 10% within the *cis* region of each gene [39, 80]. As covariates, we included the estimated global AMR ancestry, the number of nuclei within the cell-type per sample, and a varying number of pseudobulk expression PCs, which we determined on a per cell-type basis using the BE algorithm [95, 96]. *P*-values were adjusted using FDR for all tested gene-variant pairs with FDR < 0.1 as the significance threshold.

To estimate the statistical power of our cell-type-level *cis*-eQTL analysis, we used the powereQTL R package [97]. In this power analysis, we estimated the minimal detectable effect sizes for SNPs with a MAF greater than 10%, assuming at least 80% power and a family-wise error rate (adjusted α) < 0.05. We set the number of nuclei per individual to 693.7, the number of tested SNPs to 4,585,807 SNPs, and modeled gene expression as log-normally distributed with a log-scale standard deviation of 0.189, based on empirical estimates from the adipocyte data.

### Allele frequency analysis between Finns and Mexicans

To assess population differentiation between Finns and Mexicans, we used PLINK [48] to derive allele frequency estimates in Finnish METSIM and Mexican MetMex population cohorts. We only included individuals without metabolic syndrome [98] in either cohort (n_METSIM_ = 3,637, n_MetMex_ = 3,746).

### Replication assessment of the Mexican cis-eQTLs in SAT bulk and snRNA-seq cohort

To examine the SAT cell-type level *cis*-eQTLs for replications, we used Matrix eQTL to perform *cis*-eQTL mappings in the GTEx v8 SAT bulk RNA-seq data [99] and cell-type level *cis*-eQTL mappings in the SAT cell-type level pseudobulk data from the RYSA cohort [84]. We applied the same gene expression filtering criteria as in the Mexican discovery cohort. For GTEx, we included the covariates of top five genetic PCs, sequencing platform, sequencing protocol, sex, and top 60 Probabilistic Estimation of Expression Residuals (PEER) factors [80], and included variants with MAF > 0.01 in the full GTEx cohort [80]. We used the number of nuclei and top expression PCs, as determined by the BE algorithm, as the covariates for the RYSA cohort and only assessed imputed and QC passing variants with MAF > 10% in the larger Finnish KOBS (*n* = 509) cohort [100]. Testing was further limited to the significant *cis*-eQTLs in the discovery cohort, and *p*-values were adjusted using FDR. We defined replication on a per-eGene basis, and thus considered an eGene to be replicated if it had at least one *cis*-eQTL replicating as significant (FDR < 0.1) with consistent direction in the replication cohort. We additionally took the lead *cis*-eQTL per eGene and performed Wilcoxon tests per cell-type to compare the FDR-adjusted *p*-values and effect allele frequency of those that replicated in RYSA and GTEx.

To assess the Mexican cell-type level *cis*-eQTL results for replication in the recent AdipoExpress SAT bulk *cis*-eQTL meta-analysis data [101], we also conducted a separate *cis*-eQTL mapping, in which we followed the AdipoExpress analytic pipeline with minor modifications [101]. We used tensorQTL in nominal mode, which is methodologically equivalent to the APEX tool [102] used by AdipoExpress, with a *cis* window size of 1 MB around the transcription start site for the analysis. For each cell-type, we harmonized the analyses by search space, assessing only the genes and variants passing the respective testing criteria in both cohorts, and significance cut-point (FDR < 0.01), and included the same covariates as in the Matrix eQTL [94] analysis. To evaluate replication, we considered whether the eGenes in the Mexican cohort had at least one gene-variant pair reported as significant (FDR < 0.01) with a consistent direction in AdipoExpress when limiting the multiple testing to only those pairs passing FDR < 0.01 in the Mexican cohort.

### Colocalization of allele frequency stratified cis-eQTL variants

We performed colocalization analysis between the cell-type level *cis*-eQTL and GWAS signals for HDL-C, TGs, and TC using previous trans-ancestry GWAS summary statistics [88]. We focused on the genomic regions of genes that included a significant *cis*-eQTL (FDR < 0.1) and GWAS (*p*-value_GC_ < 5 × 10^−8^) variant with an allele frequency difference between Mexicans and Finns over 10% in their *cis* regions. For each region, we applied the Bayesian method coloc [103] v5.1.0 (coloc.abf) with the default prior parameters (p1 = 1 × 10^−4^, p2 = 1 × 10^−4^, p12 = 1 × 10^−5^), testing for all variants within 500 kb from the ends of the gene body that were included in the cell-type level *cis*-eQTL and GWAS analyses. A locus was defined to have a significant colocalization when the posterior probability of a shared causal variant (PPH_4_) was > 0.5 [101, 104, 105].

### Comparison of CYP26B1 SAT bulk expression using RNA-seq before and after bariatric surgery

We used limma [106] with the voom [79] normalization method to test *CYP26B1* for DE between the operation and 1-year post bariatric surgery timepoints in SAT bulk RNA-seq data from the MOSS cohort (*n* = 45). Briefly, after filtering the bulk expression data to retain only expressed genes, defined as genes with at least 1 CPM in over 10% of the samples, we applied TMM normalization. We then modeled the expression of *CYP26B1* against the bariatric surgery status, using the duplicateCorrelation method with participant ID as the blocking factor to test for differences per individual. The first three genetic PCs, as described previously [13, 40], RNA integrity number (RIN), mitochondrial read percentage, uniquely mapped read percentage, median 3’ bias, and intronic base percentage were included as covariates in the model to account for the admixed population structure and technical factors. Significance for the bariatric surgery status was defined as *p*-value < 0.05.

### Longitudinal DE analysis of CYP26B1 across human adipogenesis

We examined the expression of *CYP26B1* during human adipogenesis using previously generated [8] bulk RNA-seq data collected from six timepoints across a 14-day primary human preadipocyte differentiation experiment [107]. To assess for longitudinal DE, we ran ImpulseDE2 [108] to model the expression trajectory of *CYP26B1* and conduct a log-likelihood ratio test with a significance threshold of *p*-value < 0.05. We then used DPGP [109] with default parameters to cluster *CYP26B1* and other adipogenesis pathway genes from WikiPathway WP236 [110] into subclusters based on their longitudinal co-expression patterns over 14 days.

## Results

### SnRNA-sequencing of SAT biopsies from Mexicans with cardiometabolic phenotypes produces a rich single cell level reference

While SAT is known to exhibit cellular heterogeneity [5, 6, 18, 19, 21–23], less is known about how the contexts, such as age, sex, and ancestry, shape this heterogeneity. In particular, the contributions of SAT genes and their *cis* regulatory variants to CMDs at the cell-type or subcell-type level remain unclear, largely due to the lack of comprehensive single cell level reference data sets. To address these important knowledge gaps, we profiled a cohort of 49 Mexicans, a population with high obesity and obesity-related CMD risks [26–28], from Mexico City for relevant cardiometabolic clinical characteristics, i.e., age, sex, BMI, T2D, and lipid traits, and genome-wide genotypes as well as generated snRNA-seq data from their SAT biopsies (Fig. 1). As shown in Fig. 1B, this cohort spans a wide age range (23–70 years), comprises 12 males and 37 females, and has a roughly balanced representation of the three obesity status groups of individuals with normal weight (BMI < 25.0; *n* = 14), overweight (25.0 ≤ BMI < 30.0; *n* = 16) and obesity (BMI ≥ 30.0; *n* = 19) (see Additional file 1: Table S1 for the clinical characteristics).

Furthermore, as the Mexican population is admixed, and shaped by a 3-way flow from Europe, African, and the indigenous populations of the Americas [25], we also explored the ancestral composition of the cohort. We performed global ancestry inference and observed that the estimated proxy for the AMR component in the cohort averaged 65.0% (SD = 15.8%), which was notably higher than in the Mexican Americans from the 1000 Genomes Project (52.4%; SD = 19.4%) (Fig. 1B; Additional file 2: Fig. S1; Additional file 1: Table S2). As the effects of global AMR ancestry have not previously been investigated in genetic and single cell level omics studies of CMDs in SAT, we included the estimated global AMR ancestry as one of the tested contexts into our analyses.

To study the SAT gene expression profiles of the 49 Mexicans at a single cell resolution, we performed snRNA-seq on SAT biopsies. Following a multistep data pre-processing and quality control procedure (see Methods), we produced a Mexican single cell adipose reference of 128,057 nuclei from 15 distinct SAT cell-types (Fig. 1D) [57, 58], consistent with the cell-types identified in previous single cell surveys of SAT and providing a 49% increase in the number of nuclei from an admixed population not represented well in the recent adipose atlas study with predominantly European participants (*n* = 86,084 SAT nuclei and cells), (Additional file 2: Fig. S2; Additional file 3: Tables S3, S4) [5]. Adipocytes were generally the most abundant cell-type, followed by ASPCs, endothelial cells, and then macrophages, in line with other previously published SAT snRNA-seq datasets [5, 20–23](Additional file 2: Fig. S4; Additional file 3: Tables S4, S5); however, we did observe some variability in this pattern across individuals (Additional file 3: Table S4). Across the 15 SAT cell-types, we identified a total of 2,082 unique cell-type marker genes, i.e., the genes differentially upregulated in only one of the SAT cell-types, including previously established key canonical marker genes, such as *ADIPOQ* and *PLIN1* in adipocytes [5] (Fig. 1E; Additional file 3: Table S6). These unique marker genes were generally enriched for biological processes highly relevant to the cellular functions of the particular cell-type (Additional file 3: Table S7), supporting their roles in cell-type identity and functionality.

### Testing compositional and transcriptional profiles of the SAT cell-types for differences by contexts and CMDs

We first searched for cell-type proportion differences relating to context or CMD traits. Overall, we observed only modest compositional changes, with no drastic shifts in the main cell-type proportions across contexts or CMDs (Additional file 2: Figs. S6, 7). Adipocytes increased in prevalence with age while macrophages had higher proportions in females, in individuals with obesity, T2D, high TGs, and low HDL-C (Additional file 2: Figs. S6, 7; Additional file 3: Table S8), consistent with previous studies of cellular compositions using bulk decomposition or cell-line based counting [9, 111, 112]. These results suggest that differences in main cell-type proportions are not a major contributor to the cell-type heterogeneity in SAT.

**Fig. 2:**
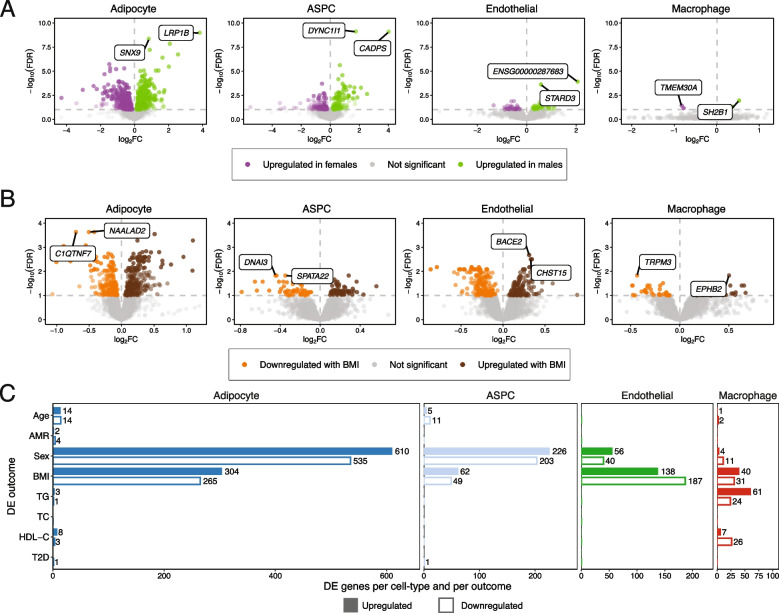
Cell-type level gene expression is influenced by various contexts and cardiometabolic disease (CMD traits. **A-B** The differential expression (DE) patterns in each cell-type by (**A**) sex (excluding genes on sex chromosomes) and (**B**) BMI are depicted in volcano plots. We plot each gene by the − log_10_(FDR-adjusted *p*-value) and log_2_fold change in expression per cell-type, color points by the direction of DE as well as the significance, and label the top two most significantly DE genes per outcome and cell-type. **C** Bar plots show the cell-type level differential expression (DE) patterns of the expressed genes in each cell-type by the contexts of age, global admixed American (AMR) ancestry, and sex, and the CMD traits of BMI, type 2 diabetes (T2D), serum triglycerides (TGs), serum high-density lipoprotein cholesterol (HDL-C), and serum total cholesterol (TC). Filled bars represent upregulated genes, and outlined bars indicate downregulated genes. For sex, upregulation indicates higher expression in males. We include the number of significant DE (FDR < 0.1) genes per outcome in each direction after every bar. Outcomes and directions with no DE genes do not contain a label

As gene expression patterns have been shown to link to interindividual variation [8, 10, 14, 80], we next sought to systematically understand the transcriptional heterogeneity by context and CMDs in each cell-type (Fig. 2). Using the CNA tool (see Methods), we detected widespread heterogeneity in all SAT cell-types on the UMAP space, quantitatively supported by global heterogeneity testing for sex, BMI, and age (Additional file 2: Figs. S8, 9; Additional file 3: Table S9). To identify more specific patterns, we performed stratified analyses within each of the four main SAT cell-types profiled. We found evidence for heterogeneity by sex in all cell-types; by BMI in adipocytes, endothelial cells, and macrophages; and by age in adipocytes (Additional file 3: Table S9). Overall, the CNA results suggest the presence of context- and CMD-associated gene expression in the full dataset as well as within specific individual cell-types.

To capture system-level transcriptional programs and gain higher-resolution insight into this heterogeneity in our data, we then performed a MOFAcell analysis [78]. We observed that latent factor 1, which explained the largest fraction of variance in the data, primarily reflected the transcriptomic variability within adipocytes (Additional file 2: Fig. S10). We also found separation by sex with latent factors 2 and 5 (FDR = 6.77 × 10–3 and 6.92 × 10–3) that was mainly driven by the adipocytes and ASPCs. Associations with BMI were driven by adipocytes and endothelial cells with latent factors 5 and 6 (FDR = 0.019 and 6.92 × 10–3), respectively (Additional file 2: Fig. S10; Additional file 3: Table S10). These MOFAcell findings indicate that among the four SAT cell-types, adipocytes particularly display heterogeneity, with sex and BMI influencing their variability.

To identify genes underlying these cell-type level differences, we performed DE analysis at the cell-type pseudobulk level by context (age, sex, and ancestry) and the CMD traits (BMI, T2D, TGs, HDL-C, and TC)**.** Across the different cell-types and outcomes assessed, we observed most DE genes in adipocytes and ASPCs, particularly by sex and BMI. However, we also identified smaller numbers of DE genes in other cell-types and for other outcomes, including 85 genes DE by TGs in macrophages, 16 genes DE by age in ASPCs, and six genes DE by global AMR ancestry in adipocytes (Fig. 2; Additional file 2: Fig. S11; Additional file 3: Table S11). We then evaluated each of these DE gene sets for functional overrepresentation and observed enrichment for immune response-related genes upregulated in ASPCs by BMI, cytoskeletal binding and cell adhesion in the genes upregulated in macrophages by triglycerides, and metabolic processes in the adipocyte genes upregulated in females, among others (Additional file 2: Fig. S11; Additional file 3: Table S12). Together, these results indicate that contexts and CMDs contribute to the SAT cell-type level gene expression profiles and functions.

### Cellular subtypes represent functional units and are linked to cell-type level co-expression networks

**Fig. 3:**
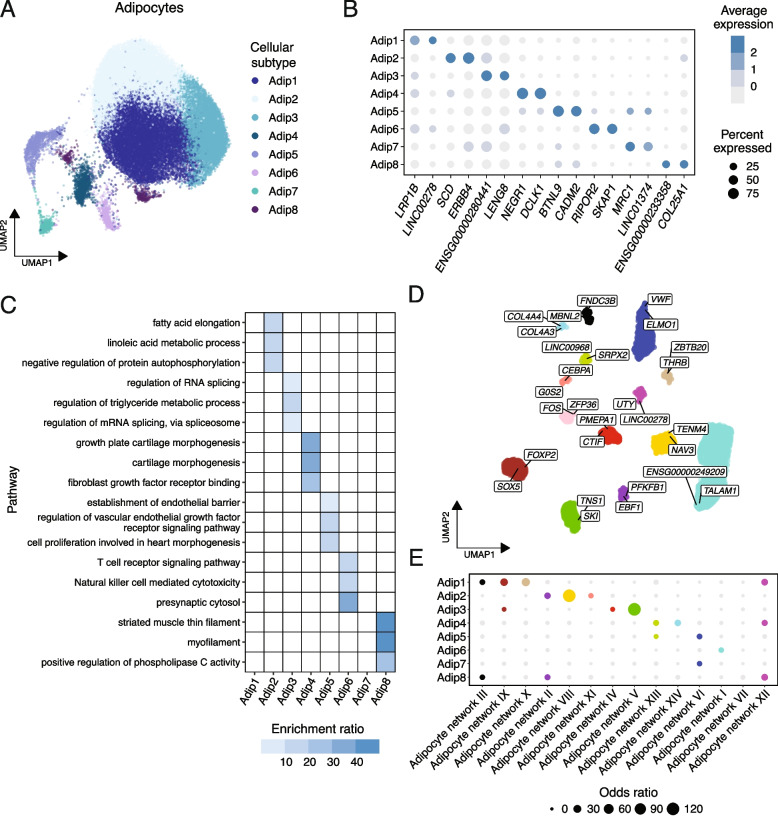
Adipocyte heterogeneity is reflecting underlying cellular subtypes and networks. **A** Uniform manifold approximation and projection (UMAP) visualization of the 34,495 nuclei broadly classified as adipocytes, colored by cellular subtype. **B** Dot plots compare the average scaled expression of the two unique subtype marker genes with the highest log_2_fold change per adipocyte subtype across the eight adipocyte subtypes. The size of each point represents the percentage of nuclei expressing the y-axis gene in the x-axis subtype, and the color intensity depicts the average expression of the gene in the subtype. **C** A heatmap visualizes the three most significantly (FDR < 0.05) enriched functional pathways for the unique subtype marker genes of each adipocyte subtype. We shade the tiles on the plot by significance of enrichment and enrichment ratio of the pathway for the subtype marker genes, with a darker shading indicating a larger enrichment ratio and sets without significant enrichment shown as white. **D** The top 25 most connected “hub” genes of each adipocyte network, i.e., the genes with the highest intra-modular connectivity score based on the correlation of expression values with the module eigengene (kME), are visualized on a supervised UMAP space. We label the two genes with the highest kME per network. **E** Dot plots show the correspondence between the adipocyte subtype unique marker genes and network genes. Each point is colored by significance (FDR < 0.05) of enrichment between the subtype unique marker genes and network genes, where the size represents the odds ratio of the overlap, and non-significant overlaps are depicted as light grey points

Our tissue and cell-type level results suggested that deeper subcell-type level differences drive cellular heterogeneity. To examine this, we partitioned the snRNA-seq expression data into the five broad cell-type groups previously defined in SAT [5, 6], subclustered each cell-type group into cellular subtypes, and determined the unique marker genes of each subtype (see Methods) (Figs. 3A, B; Additional file 2: Figs. S12a, 12b, 13a, 13b, 14a, 14b, 15a, 15b; Additional file 4: Table S13). To first confirm high quality of our data and derived subtypes, we compared the distribution of technical quality metrics at the subtype level in our data to those in the SAT snRNA-seq from the previous adipose single cell atlas [5]. We found our data quality to be comparable or better and did not observe any subtypes with extreme upper outliers in doublet scores or other artifact metrics exclusive to our cohort (Additional file 2: Fig. S16).

We also assessed the correspondence of our subtype labels with those from previously published SAT atlases [5, 21, 22, 66, 72, 73]. We observed a high concordance between our subtypes and separation of adipocytes into the classical and nonclassical adipocytes reported by the Lazarescu et al. study [21] but other adipocyte subtype labels [5, 22] did not transfer well, likely due to the known high heterogeneity of adipocytes [6, 113] (Additional file 2: Fig. S17). However, the subtypes of the other SAT cell-types matched considerably better than the adipocyte subtypes, further supporting adipocytes as the most heterogeneous cell-type in SAT rather than any issues in the subtyping procedure (Additional file 2: Figs. S16, 17).

Next, we observed that the unique marker genes of most adipocyte subtypes represent distinct functional groups, with separate subtypes harboring distinct enrichments for metabolic processes, immune activity, morphogenesis, and cardiovascular activity (Fig. 3C; Additional file 4: Tables S13, S14). Similar distinctions of developmental, immune-related, and cellular maintenance enriched subtypes were also observed among the ASPCs, myeloid, vascular, and lymphoid cell-types (Additional file 2: Figs. S12c, S13c, 14c, 15c; Additional file 4: Table S14).

We hypothesized that the subtypes may largely reflect underlying cell-type level co-expression networks, i.e., groups of genes with highly correlated expression that often represent biologically meaningful and functionally relevant transcriptomic units [114]. As these gene–gene correlations may differ between the cell-types due to the distinct biological functions of each cell-type and technical variability [82], we performed WGCNA [82, 114] separately within each broad SAT cell-type. This decomposes the transcriptome of each broad cell-type into cell-type level modules (i.e., networks) of genes with highly connected gene expression profiles. We identified 14 networks of co-expressed genes in adipocytes, and between five to nine networks in the other cell-types (Fig. 3D; Additional file 2: Figs. S12d, 13 d, 14 d, 15 d; Additional file 4: Table S15).

To first evaluate the reproducibility of our co-expression networks, we performed network preservation analysis for our cell-type level co-expression networks in a total of six external independent datasets (see Methods). We found that the vast majority of networks are highly preserved (preservation Z score ≥ 10) [85] very consistently across the six external cohorts (Additional file 2: Fig. S18), indicating that the cell-type level co-expression network structure is very robust across all SAT cell-types, including adipocytes.

Next, we checked for the overlap between the unique marker genes of a cellular subtype and the genes in a cell-type level co-expression network. In general, we observed that for most networks, their network genes showed statistically significant overlaps (FDR < 0.05) with the unique marker genes of only one subtype while the unique marker genes of a given subtype could show statistically significant overlaps with genes of multiple networks (Fig. 3E; Additional file 2: Figs. S12e, 13e, 14e, 15e; Additional file 4: Table S16). Furthermore, augmenting the detected functional enrichments at the subtype marker gene level, we found that most networks per cell-type exhibit distinct network-specific functional pathway enrichments (Fig. 3E; Additional file 2: Figs. S12f, 13f, 14f, 15f; Additional file 4: Table S17).

### A sex-dependent adipocyte co-expression network comprises replicated adipocyte function enriched genes differentially expressed by sex

**Fig. 4:**
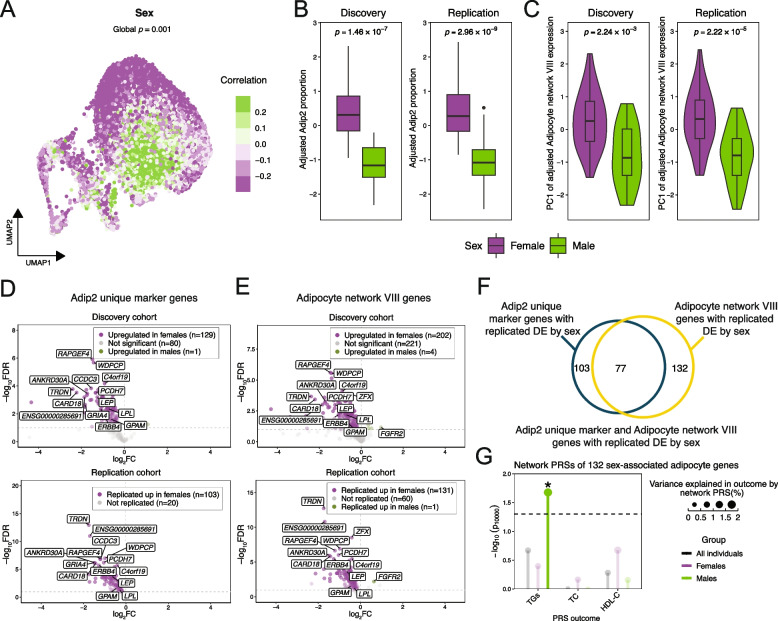
The sex-associated adipocyte subtype, Adip2, is linked to the sex-associated Adipocyte network VIII, which has 132 replicated, sex-associated network genes that confer a sex-specific polygenic risk on serum triglycerides. **A** Uniform manifold approximation and projection (UMAP) visualization of the 34,495 nuclei broadly classified as adipocytes, colored by the association of each nucleus with sex, as computed by the CNA tool [77]. The subtitle reports the *p*-value of global transcriptomic heterogeneity by the context, derived using the CNA tool [77]. **B** Boxplots compare the adjusted Adip2 proportions between females and males in the Mexican cohort (*n* = 49), i.e., the discovery cohort, and in the RYSA cohort (*n* = 68), i.e., the replication cohort. **C** Violin plots compare the first principal component (PC1) of the adjusted pseudobulk expression of Adipocyte network VIII between the females and males in the discovery cohort (*n* = 49) and replication cohort (*n* = 68). **D-E** The Adip2 unique marker genes (D) and Adipocyte network VIII network genes (E) with replicated differential expression (DE) by sex (FDR < 0.1) in the discovery and replication cohort are visualized in volcano plots. We plot each adipocyte marker gene by the –log_10_(FDR-adjusted *p*-value) and log_2_fold change in adipocyte expression, color points by the direction of DE as well as the significance (top) or replication (bottom), and label the top 10 most significant replicated DE genes per sex. **F** We show the overlap of the Adip2 unique marker genes with replicated DE by sex and Adipocyte network VIII genes with replicated DE by sex in a Venn diagram, where the area of each part is proportional to the number of genes unique to the respective group. **G** Lollipop plots compare the performance of the partitioned polygenic risk scores (PRSs) for serum triglycerides (TGs), serum high-density lipoprotein cholesterol (HDL-C), and serum total cholesterol (TC) in all individuals, males, and females built from variants in the *cis* regions of the 132 Adipocyte network VIII genes with replicated DE by sex. We color the significant TG PRSs built in males in green, where asterisks indicate a significant (*p*_perm10,000_ < 0.05) enrichment in R^2^, and the PRSs without R^2^ enrichments are colored translucent. Exact *p*-values are listed in Additional file 5: Table S23. **A**-**G** Nuclei associated with males, proportions, and PC1 values from males, genes upregulated in males, and PRS enrichments in males are colored in green, and those in females are colored in purple. **B**-**C** The *p*-value indicates the significance of difference between the females and males, as assessed by a Wilcoxon test. The box limits indicate the first and third quartiles; whiskers of each box, 1.5 × the interquartile range (IQR) from the first and third quartiles; center line, median; and points, outliers

We next searched for context and CMD -dependent compositional differences among the subtypes (Additional file 2: Figs. S19-25). We discovered that the proportions of two adipocyte subtypes, subtypes Adip1 and Adip2, significantly differ by sex (Fig. 4B; Figs. S19, 24; Additional file 4: Table S18) (see Methods). These two sex-associated subtypes, Adip1 and Adip2, corresponded to the “classical adipocytes” from the Lazarescu et al. study that are enriched for fatty acid and lipid metabolic processes [21]. Of these two subtypes, Adip2 is distinctly enriched for the genes in Adipocyte network VIII while no distinct single network enrichment was observed with Adip1 (Fig. 3E). In line with the observed Adip2 subtype proportion difference by sex and enrichment of network VIII genes among its markers, we found that the eigengene of this adipocyte co-expression network also differs by sex (Fig. 4C).

To verify these sex-associated adipocyte subtype and the corresponding network findings, we transferred the adipocyte subtypes and networks to an independent Finnish SAT snRNA-seq data set from the RYSA cohort [37, 84](*n* = 68; 72% female) and tested the adipocyte subtype proportion and network differences by sex for replication (see Methods). In these replication analyses, we observed replicated associations between the proportions of the same adipocyte subtypes Adip1 and Adip2 and sex as well as between the eigengene of Adipocyte network VIII and sex (Figs. 4B, C). Additionally, Adipocyte network VIII was not only highly preserved (Z_summary_ = 32.4) in RYSA but also ranked among the top two highest preserved networks in all six investigated external cohorts (Additional file 2: Fig. S18). These analyses robustly verify the association of both the adipocyte subtype Adip2 and Adipocyte network VIII with sex in both Mexicans and Finns.

To further elucidate the sex-dependencies observed at the subtype level, we examined whether the DE genes by sex and sex-associated MOFAcell factor genes in adipocytes are enriched in specific adipocyte subtypes (see Methods). We found enrichment in subtype Adip1 for DE genes upregulated in males (Odds ratio (OR) = 5.31; *p*_adj_ = 1.07 × 10^–9^) and in subtype Adip2 for DE genes upregulated in females (OR = 11.8; *p*_adj_ = 1.28 × 10^–53^). We also observed negative enrichment in subtype Adip1 (Enrichment score = –0.494; *p*_adj_ = 1.26 × 10^–3^) and positive enrichment in subtypes Adip2 (Score = 0.446; *p*_adj_ = 9.26 × 10^–10^) and Adip3 (Score = 0.396; *p*_adj_ = 2.76 × 10^–3^) for the genes comprising the sex-associated MOFAcell latent factor 2, and enrichments in subtypes Adip2 (Score = –0.797; *p*_adj_ = 1.60 × 10^–49^) and Adip3 (Score = 0.461; *p*_adj_ = 7.63 × 10^–5^) for the sex-associated MOFAcell latent factor 5. Overall, these enrichments provide additional support for the associations between subtypes Adip1 and Adip2 and sex.

Next, we investigated the sex-associated adipocyte subtypes for genes differentially expressed (DE) by sex. We first tested the 83 unique marker genes of subtype Adip1 for DE by sex and observed 50 DE genes, 30% of which replicated in RYSA (Additional file 4: Table S19). Testing the Adip2 unique marker genes and network genes of the corresponding Adipocyte network VIII, we then identified 130 subtype marker genes and 206 network genes DE by sex. These genes are functionally enriched for key adipocyte pathways, such as fatty-acyl-CoA metabolic and biosynthetic processes, brown fat cell differentiation, fatty acid metabolism, and triglyceride biosynthetic process (Additional file 4: Tables S20-S22), with a 77% overlap between the subtype marker genes DE by sex and the network genes DE by sex. We then evaluated these DE genes by sex for replication in the RYSA cohort and found that 84% of the Adip2 and 69% of the Adipocyte network VIII DE genes by sex replicated in RYSA (Figs. 4D-F; Additional file 4: Tables S20-S22). We also observed that 75% of the replicated subtype marker genes DE by sex overlap with the replicated network genes DE by sex (*n* = 77 overlapping replicated genes). Notably, 99% of the replicated Adipocyte network VIII DE genes by sex were upregulated in females, further confirming their co-expression. The 132 replicated Adipocyte network VIII DE genes by sex genes include four TF-encoding genes, *HIVEP3*, *EEA1*, *ZFX,* and *CHCHD3,* as well as key genes of TG biosynthesis, *GPAM*, *DGAT2*, *ACSL1,* and *LPL* [115–118] with only five genes located on sex chromosomes. Taken together, these results discover a robustly replicated sex-associated adipocyte network and its linked adipocyte subtype with many replicated genes DE by sex, involved in key pathways of adipocyte function. Overall, this elucidates how sex significantly affects adipocyte heterogeneity and function both in Mexicans and Finns.

### Network PRS shows a sex-dependent significant enrichment for serum triglycerides

We next investigated whether the replicated adipocyte genes DE by sex are also pertinent to the genetic risk for CMD traits. Due to the strong preservation of the cell-type level co-expression networks in external datasets (Additional file 2: Fig. S18), we focused on the 132 genes DE by sex in the sex-associated, highly preserved Adipocyte network VIII. Our analyses centered on lipid related outcomes due the feasibility to build PRSs for these lipid traits in the currently available Mexican cohorts with GWAS data, as well as the high dyslipidemia and hypertriglyceridemia prevalences in the Mexican population [26, 29, 31]. Thus, we constructed sex-combined and sex-stratified partitioned network polygenic risk scores from the variants in the *cis* regions of these 132 sex-associated adipocyte genes in the independent larger Mexican MetMex cohort [35] (Additional file 2: Fig. S26), using the summary statistics of the previous multi-ancestry GWAS of serum TGs, HDL-C, and TC by GLGC [88] as the base weights of the PRS model (see Methods). We found that a sex-associated network PRS for TGs significantly explained 1.70% of variance in serum TG levels strikingly only in males using 10,000 permutations (Fig. 4G; Additional file 5: Table S23). No enrichments were observed in the sex combined TG PRS, or for HDL-C and TC, indicating that this sex-dependent adipocyte network links to sex-specific genetic risk in serum TGs in Mexican males (Fig. 4G; Additional file 5: Table S23). We also observed a similar, male-specific enrichment of TG variance explained when performing the partitioned PRS analysis using the 77 replicated, adipocyte subtype Adip2 and Adipocyte network VIII genes DE by sex (Additional file 5: Table S23).

To identify sex-interacting variants within the *cis* regions of the 132 sex-associated genes, we performed a targeted variant-sex interaction (G × sex) analysis in the MetMex cohort, focusing our investigations on the variants with sex-dependent GWAS significance for TGs (see Methods). In line with the sex effects of this adipocyte network and subtype, we identified a total of 40 TG GWAS variants, spanning two independent regions near the *FFAR4/SLC35G1* and *LPL* genes, that interact with sex on serum TGs (Additional file 5: Table S24).

We then extended our partitioned PRS analysis to Europeans using the unrelated Europeans from UKB (*n* = 373,483) [41, 42]. The partitioned PRS did significantly explain variance in TGs in the Europeans of UK Biobank (*p*_all_ < 2.2 × 10^–308^, *p*_female_ = 5.36 × 10^–241^, and *p*_male_ = 1.68 × 10^–161^); however unlike in the Mexicans, we did not observe any significant enrichments in TG variance explained by the partitioned PRS in either sex (*p*_perm10,000_ > 0.05). Still, in line with the Mexican results, we also detected a statistically significant partitioned PRS × sex interaction on TGs in all individuals (β = 7.60 × 10^–3^; *p* = 0.026). In line with the male-specific PRS enrichment observed in the Mexicans, this interaction was positive, indicating a stronger effect of the PRS on TGs in males than females.

To characterize whether ancestry stratification of the male TG PRS variants could contribute to the observed differences in the partitioned TG PRS results between the Mexicans and Europeans, we computed their overall F_ST_ between Mexican men in the MetMex cohort and European men in UKB, and between the Mexican men and the all-male Finnish METSIM cohort [38] to match the population in the F_ST_ comparisons with the Finnish RYSA snRNA-seq replication cohort. Consistently in both UKB Europeans and Finns, we observed that the F_ST_ values of these partitioned TG PRS variants were significantly higher than expected by chance when assessed using 10,000 permutations (*p*_perm_UKB = 0.0007, *p*_perm_METSIM = 0.0096) (see Methods).

Notably, among the sex-DE genes, we also found multiple genes involved in key adipose tissue function to harbor variants with a high F_ST_ between the Mexicans and Europeans/Finns in their *cis* regions (Additional file 5: Table S25). These genes include 1) *GPAM*, a well-established TG and metabolic dysfunction–associated steatotic liver disease (MASLD) GWAS gene and an adipocyte marker gene involved in triglyceride synthesis [118], which harbors a variant, rs2148489, with F_ST_ > 0.25 (TG risk allele frequency (AF)_Mexicans_ = 0.614, AF_Finns_ = 0.224, AF_UKB_ = 0.225), indicating a very high differentiation [119] between the Mexicans and both European populations; 2) *DGAT2*, a lipid GWAS gene central to finalizing triglyceride synthesis [116], which ranks among the top five genes in the number of variants with F_ST_ values between 0.15 and 0.25, indicating a high differentiation between the Mexicans and Europeans in UKB; and 3) *FFAR4*, a free fatty acid receptor and a TG GWAS gene involved in both fatty acid uptake and oxidation [120], which also ranks among the top five genes in variant number with F_ST_ values of 0.15–0.25 between the Mexicans and Finns (Additional file 5: Table S25). Overall, our F_ST_ results indicate that the TG PRS variants show significant differences in AFs between the Mexican and European men, which may contribute to the absence of sex-dependent PRS enrichment in UKB.

We also assessed the variants used to construct the significantly enriched male-specific partitioned network TG PRS for enriched TF binding motifs to search for hormonal factors that may mediate the expression of these adipocyte sex DE genes and effects of their *cis* regional variants. We found that these partitioned TG PRS variant sites are enriched for the binding of progesterone receptor (PGR) (*p* = 1 × 10^–14^). PGR binds to the key female sex hormone, progesterone, which plays critical roles in regulating the menstrual cycle and early pregnancy [121]. Thus, these results indicate that the observed sex DE results and the corresponding sex-associated partitioned polygenic TG risk may be due to sex-dependent hormonal regulation via direct binding of progesterone to PGR in females but not in males at the partitioned TG PRS variant sites. Taken together, our results identify partitioned sex-specific polygenic risk for TGs from the variants residing in the sex-associated adipocyte subtype marker and network genes.

### Cell-type level cis-eQTLs elucidate distinct patterns of cis regulation of SAT gene expression per cell-type in Mexicans

**Fig. 5:**
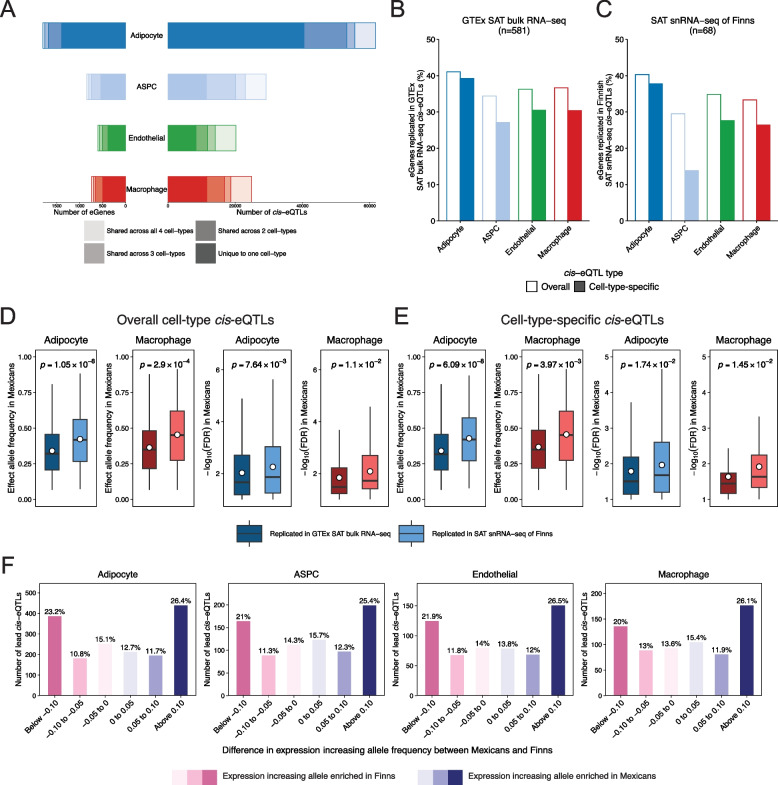
Cell-type level *cis*-eQTL analyses identify distinct patterns of *cis* regulation per cell-type. **A** Mirrored bar plots depict the number of *cis*-eQTLs and eGenes (i.e. *cis*-eQTL target genes) (FDR < 0.1), where the bars are filled proportionally by the number of cell-types, for which each *cis* regulatory signal or gene is reported. **B-C** Bar plots show the percentage of eGenes with at least one replicated cell-type level *cis*-eQTL variant (outlined bar) and at least one replicated cell-type-specific *cis*-eQTL, i.e., a *cis*-eQTL seen in only one cell-type (filled bar) that replicates in the same direction in (**B**) the subcutaneous adipose tissue (SAT) bulk tissue *cis*-eQTL mapping in the GTEx cohort (*n* = 581) and (**C**) the SAT single nucleus RNA-sequencing (snRNA-seq) cell-type level *cis*-eQTL mapping in the Finnish RYSA cohort (*n* = 68). **D-E** Boxplots compare the effect allele frequency and FDR-adjusted *p*-value, all in Mexicans, between the lead (**D**) adipocyte and macrophage, and (**E**) adipocyte-specific and macrophage-specific *cis*-eQTL variants per eGene that replicate in the SAT bulk tissue *cis*-eQTL mapping in the GTEx cohort (*n* = 581) versus those that replicate in the cell-type level mapping in the RYSA snRNA-seq cohort (*n* = 68). The *p*-value indicates the significance of the difference between the replicating signals of the lead *cis*-eQTL variants per eGene in the two cohorts, as assessed by a Wilcoxon test. The box limits indicate the first and third quartiles; whiskers of each box, 1.5 × the interquartile range (IQR) from the first and third quartiles; center line, median; and center dot, mean. **F** Bar plots show the distribution of differences in gene-expression-increasing allele frequencies (AF) between Mexicans and Finns for the lead cell-type level *cis*-eQTL variant of each eGene. Variants enriched in Mexicans are colored blue, while variants enriched in Finns are colored pink. **A**-**E** Data are colored by the cell-type, with adipocytes shown as dark blue, adipose stem and precursor cells (ASPCs) as light blue, endothelial cells as green, and macrophages as red

To search for genetic regulation of SAT gene expression at the cell-type level, we conducted cell-type level *cis*-expression quantitative trait locus (*cis*-eQTL) analysis (see Methods) on the pseudobulk expression data from the four most prevalent SAT cell-types (Fig. 5). Although our cohort size is modest, it aligns with the ones in recent cell-type level *cis*-eQTL studies [122, 123] and furthermore, our power analysis confirmed sufficient power to detect *cis*-eQTLs with reasonable effect sizes (*β* > 0.395) using MAF > 10% (see Methods) (Additional file 2: Fig. S27). We observed the most signals in adipocytes, with 61,772 *cis*-eQTL variants regulating 1,800 genes (i.e., eGenes) at FDR < 0.1, with still tens of thousands of *cis*-eQTL variants and hundreds of eGenes in the ASPCs, endothelial cells, and macrophages (Fig. 5A; Additional file 5: Table S26). While 6,176 *cis*-eQTL variants across 31 eGenes were shared across the four tested cell-types, we noted that 39–65% of the *cis*-eQTL variants and 76–87% of the eGenes were only detected in one cell-type, highlighting the notion of genetic regulation of gene expression in SAT as a cell-type level heterogeneous entity (Fig. 5A). However, larger future studies are warranted to confirm the cell-type specificity of these eQTLs.

We then evaluated replication of our cell-type level *cis*-eQTL signals in other available, primarily European SAT RNA-seq cohorts. When applying the same methodological approach as in the discovery cohort, we observed that 34–41% of the Mexican cell-type level eGenes replicated (see Methods) in SAT bulk RNA-sequencing (bulk RNA-seq) data from the GTEx cohort [80, 99] (*n* = 581) and 30–40% of the Mexican cell-type eGenes replicated in RYSA (*n* = 68), with adipocyte *cis*-eQTLs consistently having the highest replications (Figs. 5B, C; Additional file 5: Table S27). Despite the different populations used in the replication cohorts, these replication rates are well in line with those reported in previous, predominantly European cell-type level *cis*-eQTL studies [68, 124, 125]. To elucidate whether the lack of resolution in heterogeneous bulk RNA-seq data influences replications, we also examined the replication rates of the *cis*-eQTLs detected in only one cell-type, i.e. cell-type-specific *cis*-eQTLs, and cell-type shared *cis*-eQTLs, which were consistently seen in all four tested SAT cell-types. We found that the cell-type-specific *cis*-eQTLs show generally similar replication trends as the overall, whereas the replication rates of cell-type shared *cis*-eQTLs were higher than of the cell-type-specific or overall *cis-*eQTL replications and higher in single cell data than in bulk (Figs. 5B, C; Additional file 5: Table S27). Additionally, we conducted separate cell-type level *cis*-eQTL analyses using tensorQTL [126] for comparisons with the AdipoExpress SAT bulk *cis*-eQTL meta-analysis [101] (see Methods). We found overall replication rates of 58–72%, and similar replication patterns among the cell-type-specific and shared *cis*-eQTLs as in GTEx (Additional file 5: Table S27).

We next investigated factors that could be influencing the replication of *cis-*eQTLs. We observed that the average effect allele frequency (EAF) of the signals replicating in the GTEx SAT bulk RNA-seq were generally lower than those replicating in the RYSA single cell level cohort (Figs. 5D, E; Additional file 5: Table S28). Furthermore, the *p*-values of the *cis*-eQTLs that replicated in the single cell level *cis*-eQTL mappings were significantly better than those replicating in the bulk tissue (Figs. 5D, E; Additional file 5: Table S28). Overall, these results suggest that modality (bulk versus single cell), significance, allele frequency, and cell-type specificity of a *cis*-eQTL may all impact replication.

### Population stratified cis-eQTL variants identify lipid GWAS genes in Mexicans through colocalization analysis

As differences in allele frequency and linkage disequilibrium have been previously implicated behind population-specific regulatory mechanism, gene expression profiles, and disease risk [127, 128], we next searched for ancestry-associated differences in the cell-type level *cis-*eQTLs by investigating the population stratification of the *cis-*eQTL allele frequencies between Mexicans and Europeans. We first used Finnish data for these comparisons as the replication RYSA cohort is Finnish while then also investigating the allele frequency differences in the non-Finnish Europeans from the Genome Aggregation Database (gnomAD) [129]. Consistently across the four tested cell-types, we observed that nearly half of the lead *cis*-eQTLs (46–50%) differ in allele frequency between Mexicans and Finns by over 10% and an additional 23–25% differ by between 5 and 10% (Fig. 5F), highlighting widespread variation between ancestries in the variants that regulate SAT cell-type level gene expression in Mexicans. Similar results were seen in the non-Finnish Europeans from gnomAD (Additional file 2: Fig. S28).

**Fig. 6:**
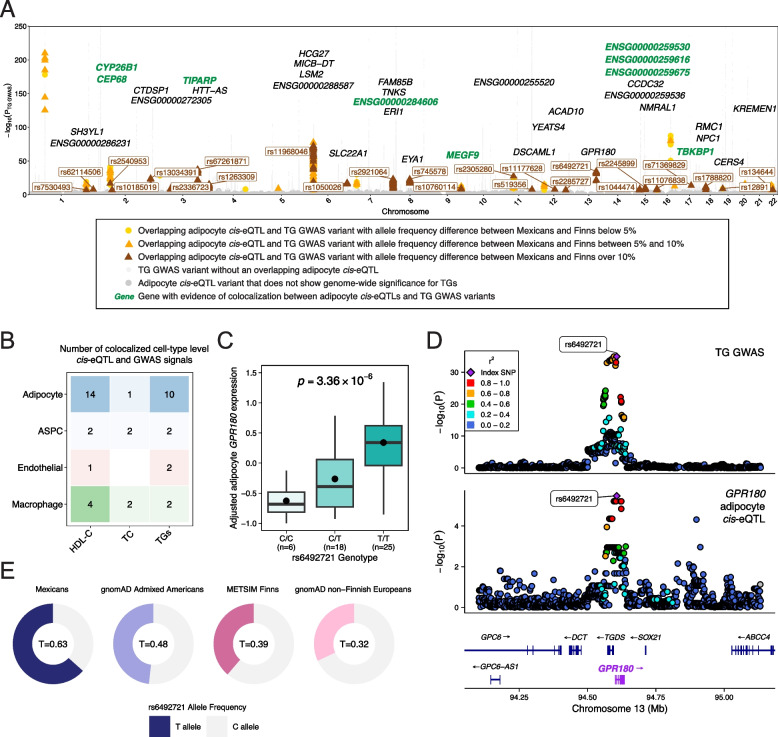
Ancestry-stratified cell-type level *cis*-eQTL variants overlap and colocalize with lipid GWAS variants. **A** GWAS variants for serum triglycerides (TGs) from the large multiethnic scan [88] and adipocyte *cis*-eQTL variants are both plotted by chromosomal position against the –log_10_(genomic control-adjusted *p*-value (*p*-value_GC_)) for the GWAS. Orange and brown triangles indicate the GWAS significant variants that overlap with the adipocyte *cis*-eQTL variants and show allele frequency (AF) differences of 5–10% and greater than 10%, respectively, between Mexicans and Finns. Gold circles denote the GWAS significant variants that overlap with the adipocyte *cis*-eQTL variants but show no population stratification (AF difference < 5%), while grey points indicate the variants that are not both *cis*-eQTL and GWAS variants. We label the most significant GWAS *cis*-eQTL variants per gene that show large population stratifications with their corresponding genes, as well as the genes with significant colocalizations (PPH_4_ > 0.5). **B** Heatmap indicating the number of significant colocalizations (PPH_4_ > 0.5) between the cell-type level *cis*-eQTL variants and GWAS variants for the three lipid outcomes, TGs, high-density lipoprotein cholesterol (HDL-C), and total cholesterol (TC). **C** The *cis*-eQTL effect of rs6492721 on *GPR180* adipocyte expression is shown through box plots, with the genotypes of rs6492721 plotted against the adjusted, normalized adipocyte pseudobulk expression of *GPR180*. The box limits indicate the first and third quartiles; whiskers of each box, 1.5 × the interquartile range (IQR) from the first and third quartiles; center line, median; and center dot, mean. **D** A regional overview of TG GWAS (upper panel) and adipocyte *cis*-eQTL variants (lower panel) demonstrates a significant colocalization of the TG GWAS and adipocyte *cis*-eQTL variant rs6492721, regulating the gene *GPR180*. The axes show the − log_10_ *p*-value_GC_ from the large multiethnic TG GWAS [88] and − log_10_ of *p*-values from the adipocyte *cis*-eQTL analysis. We indicate the lead colocalized *cis*-eQTL and TG GWAS variant, rs6492721, by a purple diamond. Colors represent the linkage disequilibrium (LD) (R^2^) of the regional variants with the colocalized *cis*-eQTL and GWAS variant, where LD was computed using the 1000 Genomes Phase 3 dataset (*n* = 2,489) [46] for the GWAS results and genotype data from the MetMex cohort (*n* = 8,375) for the adipocyte *cis*-eQTL results. Chr indicates chromosome and Mb, mega base. **E** Pie charts compare the frequencies of the TG risk allele T of rs6492721 in Mexicans, admixed Americans from gnomAD [129], Finns, and non-Finnish Europeans from gnomAD

To assess whether these population stratified, cell-type level *cis*-eQTL variants contribute to the enriched CMD risks in Mexican, we mapped the *cis*-eQTL variants from all four cell-types to the trans-ancestry lipid GWAS variants [88]. We found 65, 71, and 61 eGenes that are regulated by a population stratified *cis*-eQTL (MAF difference over 10%) that is also a GWAS variant for TGs, HDL-C, and TC, respectively (Fig. 6A; Additional file 2: Figs. S29-31; Additional file 5: Table S29). Using colocalization analysis (see Methods) among these regions, we detected evidence of colocalization between the *cis*-eQTLs and lipid GWAS variants for a total of 34 genes (Fig. 6B; Additional file 2: Figs. S29-32; Additional file 5: Table S30). We found that 25 of these 34 colocalized genes (74%) (Additional file 2: Fig. S32) have not been reported for the same colocalized lipid trait in previous SAT bulk tissue colocalization studies of Europeans [101, 130] (Additional file 2: Fig. S32). Using a MAF difference of over 10%, we observed that the 25 discovered lipid GWAS genes include 12 genes (48%) regulated by Mexican-enriched versus seven genes (28%) regulated by European enriched colocalized risk variants or their tight LD proxies (r^2^ > 0.8) (Additional file 2: Fig. S32). Taken together, our SAT single cell level *cis*-eQTL analyses followed by colocalization analyses with lipid GWAS results helped identify multiple additional lipid GWAS genes regulated at the cell-type level in *cis* by variants, enriched either in Europeans or Mexicans.

Among the 12 colocalized lipid GWAS genes regulated by a Mexican-enriched *cis*-eQTL/lipid GWAS variant, we identified a *cis*-eQTL variant, rs6492721, that colocalizes with a TG GWAS variant and regulates adipocyte expression of *GPR180* (PPH_4_ = 0.994), a component of TGFβ signaling that regulates brown and beige adipocyte function and promotes thermogenesis [131]. For rs6492721, Mexicans show a 25% higher frequency of the TG increasing allele T than Finns and 31% higher frequency than non-Finnish Europeans in gnomAD [129] (Figs. 6C-E; Additional file 5: Table S30). The colocalized genes also include three TF-encoding genes (*NPAS3, TET2, ZC3H12C*) and eight loss-of-function intolerant genes (*CLK2, CYP26B1, MEGF9, NPAS3, SCAMP5, TIPARP, YPEL3, ZC3H12C)* based on pLI score > 0.9 [132] in gnomAD, indicating that many of these identified lipid GWAS genes are loss of function-intolerant, and thus, altering their expression via *cis* effects may be functionally important. Overall, these colocalization results elucidate dyslipidemia susceptibility genes in Mexicans.

**Fig. 7:**
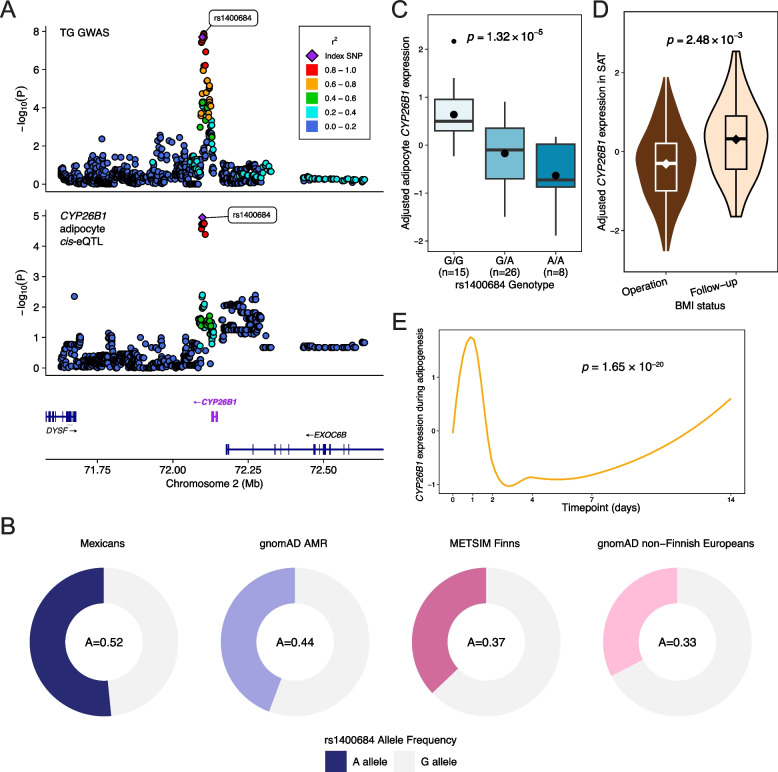
The adipogenesis gene *CYP26B1* confers its hypertriglyceridemia risk observed in GWAS via a metabolically unfavorably *cis*-eQTL effect decreasing *CYP26B1* expression in adipocytes. **A** A regional overview of serum triglycerides (TG) GWAS (upper panel) and adipocyte *cis*-eQTL variants (lower panel) demonstrates a significant colocalization of the TG GWAS and adipocyte *cis*-eQTL variant rs1400684, regulating the gene *CYB26B1*. The axes show the − log_10_ *p*-value_GC_ from the large multiethnic TG GWAS [88] and − log_10_ of *p*-values from the adipocyte *cis*-eQTL analysis. We indicate the lead colocalized *cis*-eQTL and TG GWAS variant, rs1400684, by a purple diamond. Colors represent the linkage disequilibrium (LD) (R.^2^) of the regional variants with the colocalized *cis*-eQTL and GWAS variant, where LD was computed using the 1000 Genomes Phase 3 dataset (*n* = 2,489) [46] for the GWAS results and genotype data from the MetMex cohort (*n* = 8,375) for the adipocyte *cis*-eQTL results. Chr indicates chromosome and Mb, mega base. **B** Pie charts compare the frequencies of the TG risk allele A of rs1400684 in Mexicans, admixed Americans from gnomAD [129], Finns, and non-Finnish Europeans from gnomAD. **C** The *cis*-eQTL effect of rs1400684 on *CYP26B1* adipocyte expression is shown through box plots, with genotypes of rs1400684 plotted against the adjusted, normalized adipocyte pseudobulk expression of *CYP26B1*. **D** Violin plots compare the expression of *CYP26B1* in SAT bulk RNA-sequencing data from biopsies obtained from the Mexican individuals with obesity (*n* = 45) during the bariatric surgery and one year after the surgery. The *p*-value indicates the significance of the differential expression. **C**, **D** The box limits indicate the first and third quartiles; whiskers of each box, 1.5 × the interquartile range (IQR) from the first and third quartiles; center line, median; and center dot, mean. **E** Normalized longitudinal expression of *CYP26B1* over a 14-day differentiation of human primary preadipocytes with six time-points. The *p*-value indicates the significance of the longitudinal differential expression using ImpulseDE2 [108]

Among the previously unknown colocalized signals, of particular note is also the gene, *CYP26B1*, a loss-of-function (LoF) intolerant gene that was identified for adipogenesis in earlier mouse studies [133, 134]. We identified a colocalized variant, rs1400684 (PPH_4_ = 0.901), that regulates adipocyte expression of *CYP26B1* in *cis* and is a TG GWAS variant (Figs. 7A-C). The A allele of rs1400684, which has a 15% higher frequency in Mexicans than Finns and an 18% higher frequency in Mexicans than in non-Finnish Europeans of gnomAD (Additional file 5: Table S30), increases serum TGs (Fig. 7B). Furthermore, our finding that rs1400684 decreases the adipocyte expression of *CYP26B1* in *cis* (Fig. 7C) is anticipated to be functionally important given the loss of function intolerance of *CYP26B1* with a pLI score of 1.0. This altogether suggests that lowered adipocyte *CYP26B1* expression may contribute to increased TGs in Mexicans (Figs. 7A-C).

To further understand the metabolic implication of *CYP26B1* expression in SAT, we investigated its transcriptional response to weight loss in an independent longitudinal Mexican SAT bulk RNA-seq cohort [13, 40] as well as studied its previously unknown longitudinal DE patterns during human adipogenesis. First, in the independent Mexican weight loss study, which comprises Mexicans with obesity who underwent bariatric surgery, with SAT biopsies obtained during and one year after the surgery, the SAT expression of *CYP26B1* was significantly higher after the weight loss induced by the bariatric surgery (Fig. 7D), thus revealing a metabolically beneficial association with increased *CYP26B1* expression. Second, in the longitudinal human adipogenesis experiment of human primary preadipocytes with bulk RNA-seq data collected at six time-points [8, 107] (see Methods), we observed a significant, distinct upregulation of *CYP26B1* expression over the first 24 h of preadipocyte differentiation, followed by a decline until the fourth day (Fig. 7E), and found that *CYP26B1* clustered with seven other known adipogenesis genes by its longitudinal expression (Additional file 5: Table S31), supporting its role in the early human adipogenesis and in line with its observed upregulation after bariatric surgery. Taken together, our results identify the *CYP26B1* gene as a key TG gene that is significantly upregulated by weight loss, shows dynamic DE during early human adipogenesis, and likely confers its hypertriglyceridemia risk observed in GWAS via a metabolically unfavorably *cis-*eQTL effect decreasing *CYP26B1* expression in adipocytes.

To summarize, first, our study of SAT in Mexicans at the single cell resolution discovered an adipocyte subtype, the proportions of which robustly differ by sex and 132 replicated, functionally important adipocyte genes DE by sex that reside in a highly preserved, sex-associated adipocyte co-expression network corresponding to this subtype. The sex-dependency of the 132 genes was also observed at the genetic level in the network TG PRS that significantly explains variance in serum TG levels only in Mexican males. Second, we discover 34 colocalized lipid GWAS genes, of which 25 genes represent previously undiscovered lipid GWAS genes, including 12 genes regulated by a Mexican enriched variant. These cardiometabolic GWAS risk variants exhibit large allele frequency differences between the Mexican and European populations and function as cell-type level regulatory *cis*-eQTL variants for the 34 identified lipid GWAS genes. Thus, our results discover previously unknown sex- and ancestry-stratified genes and variants, contributing to cellular interindividual heterogeneity in SAT and the genetic risk of serum lipid levels in the admixed Mexican population.

## Discussion

Subcutaneous adipose tissue, the key human fat depot for cardiometabolic health [1, 2], harbors high cellular heterogeneity likely due to interindividual differences [9–11, 14–16, 18, 19]. However, the contributions of different contexts and CMDs to these cell-type and subtype level alterations are poorly understood. Despite their rapidly-increasing prevalence of obesity and obesity-related CMDs [26, 28, 30–32], there is lack of single cell omics data in Mexicans. In this work, we used detailed cardiometabolic phenotype, genome-wide genotype, and SAT snRNA-seq data from Mexicans to characterize context- and CMD -specific patterns and their genetic regulation at the SAT cell-type and subcell-type level.

We first discover a sex-associated adipocyte subtype with a corresponding cell-type level co-expression network that harbors multiple adipocyte function enriched genes DE by sex. We then replicate these findings in an independent SAT snRNA-seq cohort. Next, we link the *cis* regional variants from this sex-associated network to a sex-dependent polygenic risk prediction of high serum TGs. Further, we identify lipid GWAS risk variants [88] that regulate cell-type level gene expression in Mexicans and show large allele frequency differences between Europeans and Mexicans. Overall, we uncover 34 colocalized lipid GWAS genes, of which 25 (74%) have not been reported in previous SAT bulk tissue colocalization studies of Europeans [101]. These 25 genes include 12 and seven genes that are *cis* regulated by a Mexican versus European enriched colocalized variant, respectively. Taken together, our work demonstrates how sex and admixed American ancestry contribute to the high predisposition to lipid disorders in Mexicans [30, 31, 34]. As Mexican-origin individuals form the largest Hispanic population in the U.S., our results are highly relevant for the large group of Mexican-origin individuals in the U.S. in addition to informing the cell-type of regulatory effects of many European enriched lipid GWAS variants.

Within SAT, transcriptomic variation carries important physiological consequences [1]. Existing studies have discerned substantial variation by sex in SAT- related cardiometabolic outcomes [135, 136] and SAT gene expression at the bulk tissue level [137, 138]. Additionally, recent SAT single cell atlases have highlighted SAT heterogeneity by contexts, including sex, and CMDs at the cell-type and subcell-type level [5, 20–23]. However, these single cell level studies have largely been conducted in European cohorts and primarily focused their subcell-type level investigations on obesity- and weight-loss-related outcomes, leaving the impacts of sex on SAT cell-type and subcell-type level gene expression poorly understood.

Our study systematically assessed the influence of sex, five CMD -related outcomes, age, and ancestry on four main SAT cell-types using a Mexican cohort with broad age range, ancestral admixture, and balanced representation of obesity categories. At the cell-type level, we first corroborated the previously reported strong influences of sex and BMI across multiple SAT cell-types [113, 139] using both transcriptomic heterogeneity assessments and DE analysis, as well as smaller alterations enriched in specific cell-types for other traits. Our DE analysis recovered several previously reported patterns, including upregulation of *XIST* and *JPX* in females and *UTY* in males across all cell-types, reflecting differences in X- and Y-chromosome linked gene expression [137, 140]. Moreover, we observed downregulation of the “thinness” gene *ALK1* in adipocytes, consistent with its known role in body weight regulation [141] and age-related changes, including upregulation of *LMO3* in ASPCs with age, as previously reported [8] and downregulation of *MACROD2*, consistent with prior bulk tissue studies of aging [142, 143], respectively. We also identified genes DE by ancestry, but fewer than for other outcomes and only in adipocytes, suggesting that direct effects of the global admixed American ancestry on SAT cell-types may be less pronounced than ancestry effects mediated by allele frequency differences of variants regulating gene expression.

Consistent with increasing evidence showing that SAT cell-type populations are heterogeneous and impacted by context and CMDs [5, 20–23], refining our study to the subcell-type level discerned additional contributions of these CMDs and contexts. Notably, when comparing our subtype labels with previously published external datasets, we found that for most cell-types, subtype labels corresponded well between cohorts, but the adipocyte concordance was limited beyond the separation of adipocytes into the classical and nonclassical adipocytes, reported by the Lazarescu et al. study [21]. These differences in adipocyte subtypes likely reflect the high individual heterogeneity of adipocytes, in line with the inconsistent marker gene overlaps observed in adipocytes previously [6]. This conclusion is further supported by our MOFAcell analysis, which found that the transcriptomic variability within adipocytes explains the largest fraction of variance in the Mexican SAT snRNA-seq data. Adipocyte heterogeneity is likely impacted by specific individual contexts that may differ between the cohorts, with known influences by sex, obesity level, and other CMD traits [5, 22, 23, 113, 144].

In addition to subtypes, groups of genes with highly correlated expression within each cell-type, also known as co-expression networks or modules, may represent biologically meaningful and functionally relevant transcriptomic units [114]. We found that these cell-type level co-expression networks, including those in adipocytes, are robustly preserved across independent external cohorts, indicating that the cell-type level co-expression network structure is robust and highly reproducible.

Our subtype and network assessment centered around the discovery of a sex-associated adipocyte co-expression network that corresponds to a sex-associated “classical” adipocyte subtype and includes 132 genes with replicated sex-associated expression in adipocytes. Among these sex-associated genes, there are key contributors to adipocyte function and classical adipocyte markers, such as *LPL* [115], *VLDLR* [145], *FFAR4* [120], *DGAT2* [116], *LEP*; and an X-chromosomal gene, *CHRDL1*, that has been implicated in the regulation of adipogenesis [146]. The expression of *LPL* and *CHRDL1* has also been inversely associated with lower lipid levels [147, 148]. By integrating these sex-associated signatures with large scale population DNA level data, we determined that the regional variants of these co-expressed, sex-associated genes both interact with sex on serum TG levels and harbor heightened polygenic risk for elevated serum TGs in Mexican males, but not in females.

We discovered PGR-mediated progesterone regulation at the TG PRS variant sites within the sex DE gene regions, which likely influences both their sex-associated expression and corresponding sex-specific genetic TG risk. This regulation, i.e., progesterone binding PGR at the TG PRS variant sites, is present only in females because progesterone is a female-specific hormone. Thus, the progesterone binding at the sites may attenuate TG risk by increasing expression of the sex DE genes, in line with 99% of the DE genes being more highly expressed in females than males. The upregulation of these genes could protect females against high TGs, whereas in males binding of the female sex-hormone progesterone will not take place at these PGR sites, resulting in lower expression of the genes and higher TG risk.

Additionally, we found enriched population stratification at the PRS TG variants sites between the Mexican and European men, which may explain the observed differences in replication of partitioned TG PRS effects across these ancestries. Thus, while the DE results by sex are universally observed across both Mexican and Finns, likely reflecting progesterone effects mediated by PGR both in Mexican and Finnish females, the sex-dependent partitioned TG PRS enrichments only seen in Mexicans are likely due to the ancestry-stratification between Mexicans and Europeans at the *cis* regional variant sites of the sex DE genes. Together, these findings expand the understanding of sex-associated adipocyte function by identifying sex-specific contributions to adipocyte heterogeneity and lipid risk via a sex-specific subtype, co-expression network, and DE genes under sex-specific hormonal regulation. This emphasizes the importance of analyzing gene expression at the subcell-type level when studying cellular heterogeneity of SAT.

While the genetic regulation of SAT tissue level gene expression has previously been established using bulk-RNA sequencing data [39, 80], our study utilizes the cell-type level expression derived from single cell level data to explore the patterns of *cis* regulation per cell-type in Mexicans. In line with recent single cell -based analyses of *cis* regulation in other tissues [149–151], we identified genetic *cis* regulation both shared across the cell-types and specific to one cell-type. We showed that *cis*-eQTLs from adipocytes generally have the best replications in SAT bulk and snRNA-seq cohorts, consistent with them having the best power for being captured in *cis*-eQTL mappings as the most prevalent cell-type in SAT [80]. However, we also determined that sample size, modality (bulk versus single cell), and ancestry all influence replication rates of these cell-type level *cis*-eQTLs, likely reflecting the contributions of sample size and allele frequencies on the statistical power for *cis*-eQTL discovery [80]. Overall, our results highlight the importance of large transcriptomic datasets of SAT in Mexicans to optimize the discovery of regulatory variants in SAT at the single cell resolution.

Due to the lack of Mexican data in existing omics studies of CMDs, the genes and mechanisms underlying the Mexican population’s increased risk for dyslipidemia and hypertriglyceridemia [30, 31, 33] have remained largely elusive. Although ancestry-specific effects on gene expression have generally been challenging to disentangle, likely due to their small effect sizes, previous studies [127, 128] have suggested that ancestry-associated differences in gene expression relate closely linked to the allelic signatures and LD patterns of their *cis* regulatory regions. Consistent with these existing works, while we detected limited evidence of cell-type level DE by the admixed American ancestry, we found widespread population-based variation in allele frequencies of the cell-type level *cis*-eQTL variants. We recognize, however, that identification of small to moderate differences in SAT cell-type level gene expression associated with the global admixed American ancestry may require larger cohorts. By integrating the identified cell-type level *cis*-eQTL variants that exhibit allelic differences across Europeans and Mexicans with large lipid GWASs, we discovered ancestry-stratified colocalizations between lipid risk and the *cis* regulation of SAT cell-type level gene expression. The identified colocalizations not only defined the cell-type of origin for nine previous SAT bulk tissue colocalized genes detected in Europeans, such as the *TIPARP* locus for TGs [101, 130] and *NPAS3* for HDL-C [101], but also discovered 25 previously unknown lipid GWAS genes.

Among the 25 lipid GWAS genes not reported in previous SAT bulk tissue studies [101, 130], 12 genes exhibit colocalized variants enriched in Mexicans. One such example is rs6492721, which colocalizes as an adipocyte *cis-*eQTL for the key thermogenesis-involved gene *GPR180* [131] and TG GWAS variant in the previous trans-ancestry study [88]. As the major allele in Mexicans and minor allele in Europeans, the TG risk allele T has a large, 25–30% higher prevalence in Mexicans compared to Europeans and was observed to increase *GPR180* expression in adipocytes in the Mexican SAT snRNA-seq data. We also observed that rs6492721 is replicated for its *cis*-eQTL effect on *GPR180* in both SAT and VAT in the previously published *cis*-eQTL data from GTEx [80]. In a previous in vitro human and mous*e* study [131], silencing of *GPR180* in human adipose-derived stem cells led to reduced *UCP1* expression on both the mRNA and protein levels. They further demonstrated the role of *Gpr180* in the regulation of mouse brown adipose tissue development and function via TGFβ signaling through *Gpr180* knockout mice [131]. *Gpr180* has also been observed to inhibit lipogenesis in cultured mouse adipocytes [152]. Thus, a metabolically disadvantageous upregulation of *GPR180* in Mexicans, driven by the colocalized variant, may reduce fatty acid uptake in adipocytes, thereby elevating circulating TG levels. Future studies are warranted, however, to comprehensively characterize the metabolic consequences of *GPR180* in human adipocytes.

We also identified a previously unknown colocalization in SAT adipocytes between the regulation of a LoF-intolerant gene, *CYP26B1*, and TGs for the adipocyte *cis*-eQTL variant rs1400684. While LoF-intolerant genes are typically constrained for *cis*-eQTL variants, they are often enriched for GWAS risk variants [132], suggesting that their *cis*-eQTL variants are likely of high clinical relevance. In our study, the TG risk allele A of the colocalized variant rs1400684 reduces adipocyte *CYP26B1* expression and exhibits a 15–18% higher prevalence in Mexicans than in Europeans, indicating that its downregulation poses a metabolic disadvantage in Mexicans. Supporting this, *Cyp26b1* knockout mice exhibit atrophied and dysfunctional adipose tissue [153] and mouse *Cyp26b1* expression decreases with obesity [154]. We also show this metabolic relation in humans using SAT RNA-seq from Mexicans, where we observed a significantly lower *CYP26B1* expression in SAT after weight loss by bariatric surgery. Additionally*, CYP26B1* has been suggested as a potential adipogenesis gene largely based on mouse models [133, 134], but its role in human adipogenesis remains elusive. Leveraging longitudinal data collected during human adipogenesis, we discovered that *CYP26B1* was longitudinally DE during human primary preadipocyte differentiation with particularly dynamic changes during the first four days, and temporally clustered with seven other known adipogenesis genes, including a lipodystrophy gene, *LMNA* [155] and a TF-encoding gene, *NCOR2*, previously implicated in obesity-associated adipose tissue inflammation [156], supporting its involvement during early human adipogenesis. Overall, these results discover *CYP26B1* as a key TG-associated gene that may confer a hypertriglyceridemia risk via a metabolically unfavorably *cis*-eQTL effect that impairs SAT function through decreasing *CYP26B1* expression in adipocytes.

While our study offers an increased understanding of the sex and population-based differences in SAT and their relationships to CMD risk, we recognize several limitations. We focused on the single cell level variation in SAT gene expression, but it would also be important to study additional genomic layers in SAT at the single cell level, such as chromatin accessibility. Furthermore, the lack of the Mexican SAT snRNA-seq cohorts precluded direct replication investigations of ancestry specific effects and thus, our replications were conducted using data from individuals of primarily European ancestry. Future even larger, independent snRNA-seq studies in Mexicans are warranted to verify the effects of the contexts studied here, examine the effects of additional phenotypic factors, e.g. socioeconomic factors, and increase power for detecting additional cellular and subtype level differences and *cis* regulatory signals. In our genetic risk assessments, we employed summary statistics from the large-scale multi-ancestry GWASs containing Mexicans [88] to build PRSs and conduct colocalization analysis. However, using large Mexican-only GWAS summary statistics, or exploring the polygenic risk contributions of the highly polygenic obesity phenotype, could be investigated next when larger Mexican GWASs of obesity and CMDs become available in the future.

## Conclusions

Overall, we provide a characterization of the SAT cell-type and subcell-type profiles in Mexicans as they relate to sex, admixed American ancestry, genetic cell-type level *cis* regulation, and CMD risk. Our study improves understanding of complex cell-type level, sex-specific gene expression, genetic regulation, and links to dyslipidemia risk in two ways. First, we identify a sex-associated adipocyte co-expression network that corresponds to a sex-associated adipocyte subtype and harbors sex-associated genes, conferring a sex-dependent polygenetic risk for serum TGs. Second, we discover colocalized genes regulated at the cell-type level by ancestry-stratified *cis*-eQTL and lipid GWAS risk variants, thus discovering the underlying gene for 28 lipid GWAS variants. Taken together, our study refines the understanding of SAT heterogeneity and its implications for cardiometabolic health in the admixed Mexican population.

## Supplementary Information

Additional file 1: Tables S1 and 2. Excel-formatted files for Tables S1 and 2. Table S1: Clinical characteristics of the Mexican subcutaneous adipose single nucleus RNA-sequencing cohort (*n*=49). Table S2: The estimated global AMR ancestry is higher in the Mexican snRNA-seq cohort than in most admixed American populations from the 1000 Genomes Project (1000G).

Additional file 2: Supplementary Figures. Figs. S1-32. Fig. S1: Subcutaneous adipose tissue single nucleus RNA-sequencing cohort from Mexico City has a high average proportion of estimated global admixed American (AMR) ancestry. Fig. S2: Multi-step quality control of SAT snRNA-seq data from 49 Mexican individuals produces a large SAT single cell reference. Fig. S3: The contexts and cardiometabolic disease (CMD) traits show significant correlations in the Mexican study cohorts. Fig. S4: The proportions in the Mexican SAT snRNA-seq cohort of the four main cell-types are comparable to those of previously published SAT snRNA-seq cohorts. Fig. S5: Principal component analysis of cell-type level pseudobulk gene expression per sample. Fig. S6: Comparisons of the cell-type proportions derived from the single cell level data by context and CMD traits detect relatively minor differences. Fig. S7: The proportions of main cell-types and cellular subtypes differ by binary traits (sex and type 2 diabetes (T2D) status) and correlate with continuous contexts and CMD traits. Fig. S8: The Mexican SAT snRNA-seq data shows statistically significant differences by sex, BMI, and age on the reduced dimension space. Fig. S9: Single nucleus RNA-sequencing data of SAT biopsies from 49 Mexican individuals shows differences in the UMAP space by the CMD traits of type 2 diabetes (T2D), serum triglycerides (TGs), serum total cholesterol (TC), and serum HDL-cholesterol (HDL-C). Fig. S10: Multi-cellular factor analysis (MOFAcell) [78] reveals that particularly adipocytes display strong cellular heterogeneity, with sex and BMI influencing their variability. Fig. S11: Ancestry and total serum triglycerides affect gene expression in adipocytes and macrophages, respectively, and the differentially expressed genes by BMI, sex, and triglycerides show functional enrichments. Fig. S12: Adipose stem and precursor cells (ASPCs) contain functionally distinct subtypes and cell-type level co-expression networks. Fig. S13: Subtypes and cell-type level co-expression networks within the vascular cell-types. Fig. S14: The subtypes and cell-type level co-expression networks within the lymphoid cell-types capture distinct subtype functions. Fig. S15: Subtypes and cell-type level co-expression networks within the myeloid cell-types. Fig. S16: Quality of the Mexican SAT snRNA-seq data compared to that of the SAT snRNA-seq of a previously published adipose single cell atlas [5, 66]. Fig. S17: Comparisons of the identified subtypes per cell-type with SAT subtype annotations from published atlases [5, 21, 22, 66, 72, 73]. Fig. S18: The SAT cell-type level co-expression networks are highly preserved consistently across six independent external datasets. Fig. S19: Comparisons of the adipocyte subtype proportions by context and CMD traits detect sex differences among two adipocyte subtypes. Fig. S20: The ASPC subtype proportions exhibit minor differences by CMD context and CMD trait. Fig. S21: Proportions of the lymphoid subtypes show minor differences by CMD context and CMD trait. Fig. S22: Comparisons of myeloid subtypes identify a myeloid subtype associated with BMI. Fig. S23: The vascular subtypes show minor differences in proportions by CMD context and CMD trait. Fig. S24: The proportions of cellular subtypes differ by binary traits (sex and type 2 diabetes (T2D) status) and correlate with continuous contexts and CMD traits. Fig. S25: The proportions of cellular subtypes are correlated with continuous contexts and CMD traits. Fig. S26: Principal component analysis of the MetMex cohort on the 1000 Genomes PC space. Fig. S27: Power estimation analysis indicates that we have adequate power (>80%) to detect cell-type level *cis*-eQTLs with realistic effect sizes. Fig. S28: Cell-type level *cis*-eQTL variants differ in their allele frequencies between the Mexicans and non-Finnish Europeans from gnomAD [129]. Fig. S29: The ancestry-stratified ASPC, endothelial cell, and macrophage *cis*-eQTL variants overlap and colocalize with GWAS variants for serum triglycerides in the large trans-ancestry GWAS [88]. Fig. S30: The ancestry-stratified cell-type level *cis*-eQTL variants overlap and colocalize with GWAS variants for serum HDL-cholesterol in the large trans-ancestry GWAS [88]. Fig. S31: The ancestry-stratified cell-type level *cis-*eQTL variants overlap and colocalize with GWAS variants for serum total cholesterol in the large trans-ancestry GWAS [88]. Fig. S32: Of the colocalized genes, 25 genes have not been reported in previous SAT bulk colocalization studies [101], including 12 genes regulated by Mexican-enriched and 7 genes regulated by European enriched colocalized risk variants.

Additional file 3: Tables S3 to 12. Excel-formatted files for Tables S3 to 12. Table S3: Summary of the prediction confidence scores per cell-type in the Mexican SAT snRNA-seq data (*n*=49). Table S4: Summary of the proportions of nuclei from each identified cell-type in the SAT snRNA-seq data (*n*=49). Table S5: The cell-type proportions in the Mexican SAT snRNA-seq cohort of the four main cell-types are comparable to those of previously published SAT snRNA-seq cohorts. Table S6: Unique marker genes identified in each cell-type. Table S7: Unique marker genes show significant overrepresentation (FDR<0.05) of functional pathways related to their cell-type functions. Table S8: Comparisons of the proportions of the four main cell-types by contexts and CMD traits. Table S9: CNA detects significant heterogeneity (p<0.05) by contexts and CMD traits at the tissue and cell-type level resolutions. Table S10. Multi-cellular factor analysis (MOFAcell) reveals transcriptomic variability in SAT cell-types associated with sex, BMI, and HDL-cholesterol. Table S11: Differential expression patterns at the cell-type level are strongest by the outcomes of BMI and sex. Table S12: Genes differentially expressed in each cell-type by contexts and CMD traits show significant (FDR<0.05) functional enrichments.

Additional file 4: Tables S13 to 22. Excel-formatted files for Tables S13 to 22. Table S13: Unique marker genes of each cellular subtype. Table S14: The unique marker genes of the cellular subtypes show significant (FDR<0.05) overrepresentation of biological pathways. Table S15: Top 100 most highly connected genes in each of the cell-type level co-expression networks. Table S16: Network genes of the cell-type level networks overlap with the unique marker genes of cellular subtypes. Table S17: Cell-type level networks of co-expressed genes contain distinct, significant (FDR<0.05) enrichments of biological pathways and processes. Table S18: Comparisons of the subtype proportions by contexts and CMD traits. Table S19: Among the unique marker genes of adipocyte subtype Adip1, 15 genes show replicated differential expression by sex in adipocytes. Table S20: Of the unique marker genes of adipocyte subtype Adip2, 103 genes show replicated differential expression by sex in adipocytes. Table S21: Adipocyte network VIII has 132 genes which show replicated differential expression by sex in adipocytes. Table S22: The adipocyte subtype Adip2 unique marker genes and Adipocyte network VIII genes differentially expressed (DE) by sex show significant (FDR<0.05) enrichments of key adipocyte function-related pathways.

Additional file 5: Tables S23 to 31. Excel-formatted files for Tables S23 to 31. Table S23: TG polygenic risk scoresbuilt from the sex-associated Adip2 marker and Adipocyte network VIII genes show sex-specific polygenic risk enrichment for variance explained in TGs in the MetMex cohort (*n*=8,375). Table S24: Variants residing within the *cis* regions of the 132 Adipocyte network VIII genes interact with sex on TGs in the MetMex cohort (*n*=8,375). Table S25: The 132 sex-associated network genes harbor variants in their *cis* regions with high F_ST_ between Mexican and European men. Table S26: Lead *cis*-eQTLs per eGene in the cell-type level *cis*-eQTL mapping. Table S27: Replication rates of the cell-type level *cis*-eQTL mappings in SAT RNA-seq cohorts. Table S28: Differences in significance and effect allele frequency in Mexicans between the *cis*-eQTLs replicating in SAT bulk tissue versus cell-type level mappings. Table S29: List of cell-type level eGenes that are regulated by *cis* regional lipid GWAS variants with allele frequency differences between Mexicans and Finns over 10%. Table S30: Ancestry-stratified cell-type level *cis*-eQTL variants colocalize with lipid GWAS variants. Table S31: *CYP26B1 *temporally co-clusters with seven other known adipogenesis genes in its expression trajectory over a 14-day primary human preadipocyte differentiation.

## Data Availability

The Mexican SAT snRNA-seq data are available in the NIH Gene Expression Omnibus (GEO), under accession number GSE316698 (https://www.ncbi.nlm.nih.gov/geo/query/acc.cgi?acc=GSE316698) [57], and in the Single Cell Portal, under study number SCP3602 (https://singlecell.broadinstitute.org/single_cell/study/SCP3602) [58]. The Mexican cell-type level cis-eQTL summary level data and the full summary results of the DE analysis are available on Zenodo at https://zenodo.org/records/19080953 [157]. The RYSA SAT snRNA-seq data were made available previously in NIH GEO, under accession number GSE274778, (https://www.ncbi.nlm.nih.gov/geo/query/acc.cgiacc=GSE274778) [84]. The bulk RNA-seq data from the primary human preadipocyte differentiation experiment were previously made available in GEO, under accession number GSE249195 (https://www.ncbi.nlm.nih.gov/geo/query/acc.cgi?acc=GSE249195) [8, 107]. The SAT snRNA-seq data from Emont et al. [5] were deposited at Single Cell Portal, under study number SCP1376 (https://singlecell.broadinstitute.org/single_cell/study/SCP1376) [66]. The SAT snRNA-seq data from Miranda et al. [22] were made available previously in NIH GEO, under accession number GSE295708 (https://www.ncbi.nlm.nih.gov/geo/query/acc.cgiacc=GSE295708) [72]. The SAT snRNA-seq data from Lazarescu et al. [21] were made available in NIH GEO, under accession number GSE281356 (https://www.ncbi.nlm.nih.gov/geo/query/acc.cgiacc=GSE281356) [73]. The GTEx whole-genome sequencing and gene expression data are available at dbGaP phs000424.v8.p2 (https://dbgap.ncbi.nlm.nih.gov/study/phs000424.v8.p2) [80, 99]. Data access to the existing MOSS [13] and MetMex cohorts [35] are described in the original publications cited here for each cohort. The data that support the findings in this manuscript are available from the UKB. However, restrictions apply to the availability of these data, which were used in this study under UKB application number 33934. UKB data are available for bona fide researchers through the application process at https://www.ukbiobank.ac.uk/learn-more-about-uk-biobank/contact-us. The analysis code is available on GitHub (https://github.com/ashakar/Mexican_SAT_snRNA_2026) [158].

## Abbreviations

AF: Allele frequency
AMR: Admixed American
ANOVA: Analysis of variance
ASPC: Adipose stem and precursor cell
BMI: Body mass index
Bulk RNA-seq: Bulk RNA-sequencing
CMD: Cardiometabolic disease
CNA: Covarying neighborhood analysis
CPM: Counts per million
DE: Differential expression
eQTL: Expression quantitative trait locus
F_ST_: Fixation index
GLGC: Global Lipids Genetics Consortium
gnomAD: Genome Aggregation Database
GO: Gene Ontology
GWAS: Genome-wide association study
HDL-C: Serum high-density lipoprotein cholesterol
HWE: Hardy-Weinberg Equilibrium
LD: Linkage disequilibrium
LoF: Loss of function
MAF: Minor allele frequency
MASLD: Metabolic dysfunction–associated steatotic liver disease
METSIM: METabolic Syndrome In Men
MetMex: Mexican metabolic
MOFAcell: Multi-cellular factor analysis
MREC: Multi-centre Research Ethics Committee
MOSS: Mexican Obesity Study
OR: Odds ratio
*p*_adj_: Bonferroni-adjusted *p*-value
PC: Principal component
pANN: Proportion of artificial nearest neighbors
PEER: Probabilistic Estimation of Expression Residuals
PGR: Progesterone receptor
PPH4: Colocalization posterior probability of hypothesis 4
PRS: Polygenic risk score
QC: Quality control
RIN: RNA integrity number
RTB: Research Tissue Bank
RYSA: Roux-en-Y versus one-anastomosis gastric bypass
SAT: Subcutaneous adipose tissue
SD: Standard Deviation
SNP: Single nucleotide polymorphism
snRNA-seq: Single nucleus RNA sequencing
TC: Total cholesterol
TF: Transcription factor
TGs: Triglycerides
TMM: Trimmed Mean of M-values
TPM: Transcripts per million
T2D: Type 2 diabetes
