# Supplementary material for: Lessons from single cell omics: admixed American ancestry and sex confer cardiometabolic disease risk in Mexicans
Source: Genome Med. 2026 Mar 28;18:57. doi: 10.1186/s13073-026-01633-x (PMC13151335; doi:10.1186/s13073-026-01633-x)
Supplement: Supplementary file 2 — Additional file 2: Supplementary Figures. Figs. S1-32. Fig. S1: Subcutaneous adipose tissue single nucleus RNA-sequencing cohort from Mexico City has a high average proportion of estimated global admixed American (AMR) ancestry. Fig. S2: Multi-step quality control of SAT snRNA-seq data from 49 Mexican individuals produces a large SAT single cell reference. Fig. S3: The contexts and cardiometabolic disease (CMD) traits show significant correlations in the Mexican study cohorts. Fig. S4: The proportions in the Mexican SAT snRNA-seq cohort of the four main cell-types are comparable to those of previously published SAT snRNA-seq cohorts. Fig. S5: Principal component analysis of cell-type level pseudobulk gene expression per sample. Fig. S6: Comparisons of the cell-type proportions derived from the single cell level data by context and CMD traits detect relatively minor differences. Fig. S7: The proportions of main cell-types and cellular subtypes differ by binary traits (sex and type 2 diabetes (T2D) status) and correlate with continuous contexts and CMD traits. Fig. S8: The Mexican SAT snRNA-seq data shows statistically significant differences by sex, BMI, and age on the reduced dimension space. Fig. S9: Single nucleus RNA-sequencing data of SAT biopsies from 49 Mexican individuals shows differences in the UMAP space by the CMD traits of type 2 diabetes (T2D), serum triglycerides (TGs), serum total cholesterol (TC), and serum HDL-cholesterol (HDL-C). Fig. S10: Multi-cellular factor analysis (MOFAcell) [78] reveals that particularly adipocytes display strong cellular heterogeneity, with sex and BMI influencing their variability. Fig. S11: Ancestry and total serum triglycerides affect gene expression in adipocytes and macrophages, respectively, and the differentially expressed genes by BMI, sex, and triglycerides show functional enrichments. Fig. S12: Adipose stem and precursor cells (ASPCs) contain functionally distinct subtypes and cell-type level co-expression network [file 13073_2026_1633_MOESM2_ESM.pdf]

Supplementary Figures

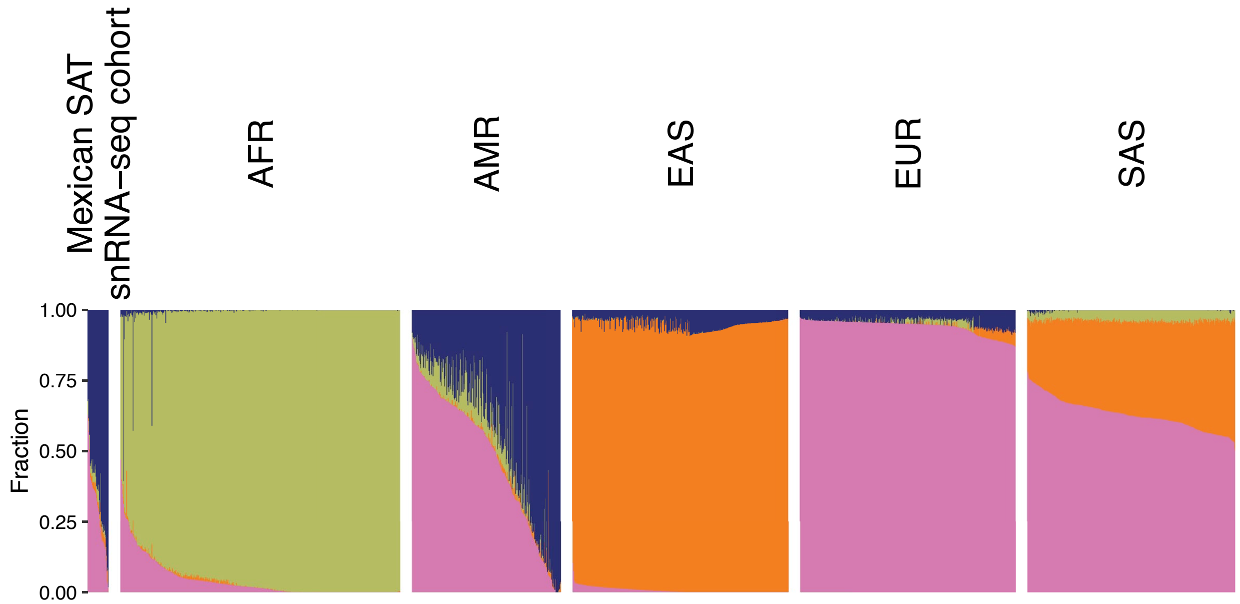

Fig. S1. Subcutaneous adipose tissue single nucleus RNA-sequencing cohort from Mexico City has a high average proportion of estimated global admixed American (AMR) ancestry.

The distribution of the estimated global admixed American, European, East and South Asian, and African ancestral components from unsupervised ADMIXTURE[44] (k=4) is shown for the Mexican single nucleus RNA-sequencing cohort (n=49) and the five defined 1000 Genomes superpopulations. In every panel, each line represents an individual, and the lines are sorted by the estimated admixed American component. The admixed American super population is shown as AMR, colored dark blue; European EUR, pink; East and South Asian EAS, orange; and African AFR, green. Mexican SAT snRNA-seq cohort indicates the Mexican single nucleus RNA-sequencing cohort.

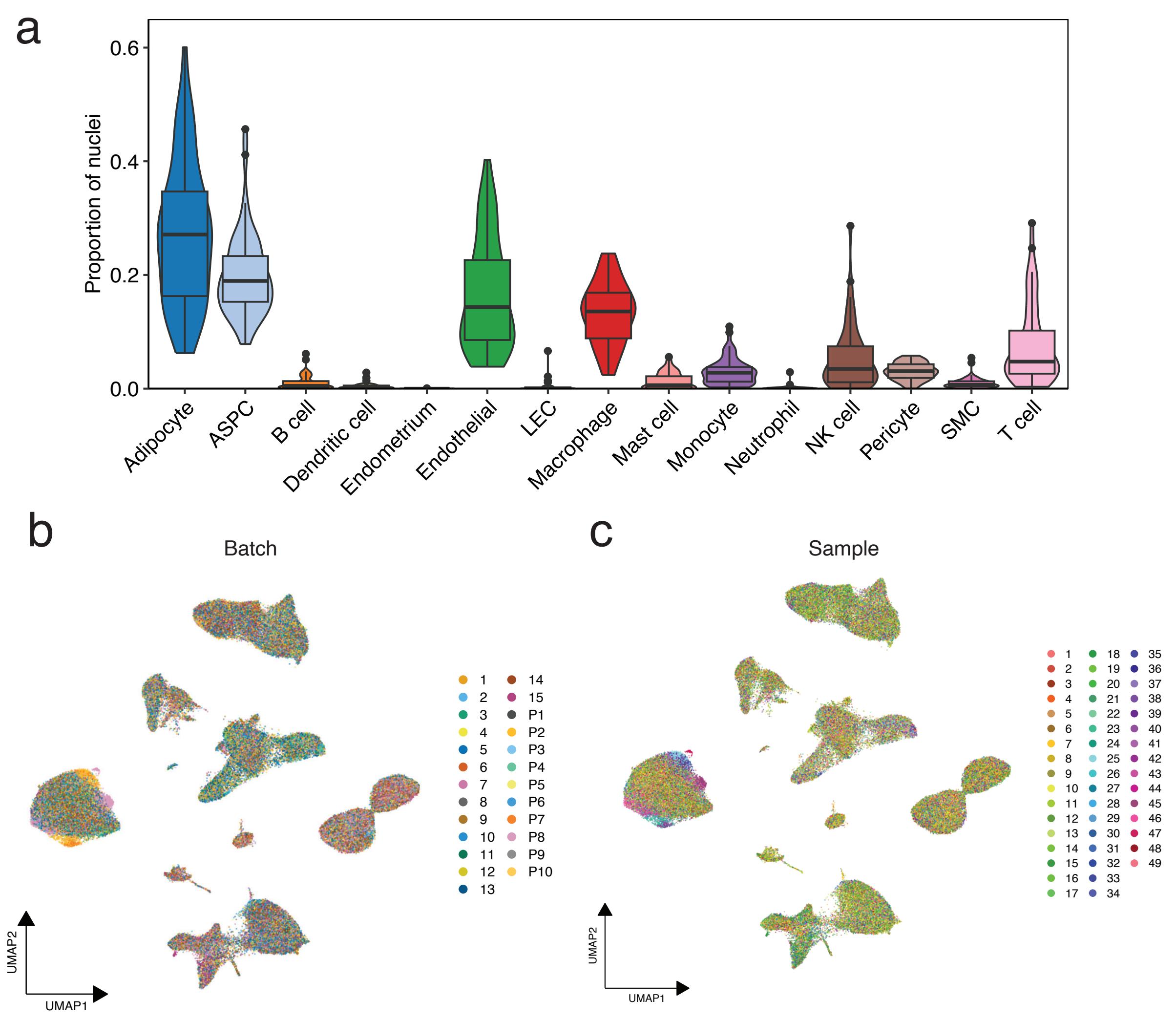

Fig. S2. Multi-step quality control of SAT snRNA-seq data from 49 Mexican individuals produces a large SAT single cell reference.

(a) Violin plots embedded with boxplots show the distributions of cell-type proportions per cell-type and individual, where cell-type proportion is defined as the proportion of nuclei from the individual annotation of the cell-type. The box limits indicate the first and third quartiles; whiskers of each box, 1.5 × the interquartile range (IQR) from the first and third quartiles; center line, median; and points, outliers.

(b-c) UMAP visualizations of the 128,057 nuclei from 49 individuals are colored by the b) sequencing batch of the samples and c) sample.

ASPC indicates adipose stem and precursor cells, LEC lymphatic endothelial cells, and SMC smooth muscle cells.

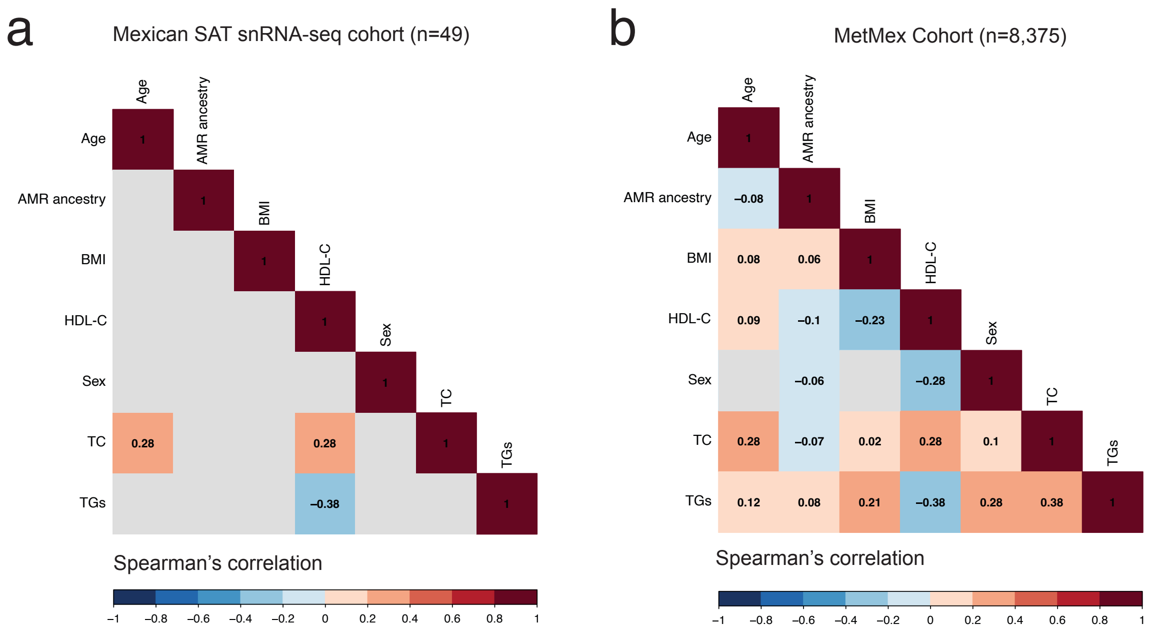

Fig. S3. The contexts and cardiometabolic disease (CMD) traits show significant correlations in the Mexican study cohorts.

(a-b) The pairwise Spearman's correlations between age, global admixed American (AMR) ancestry, serum total cholesterol (TC), serum triglycerides (TGs), serum HDL-cholesterol (HDL-C), BMI, and sex are shown in the a) Mexican SAT snRNA-seq cohort (n=49) and b) MetMex cohort (n=8,375). Blue boxes correspond to a significant (Spearman's $p<0.05$) negative correlation, red boxes correspond to a significant positive correlation, and grey boxes correspond to a non-significant correlation. For the significant correlations, the Spearman's coefficient is also displayed.

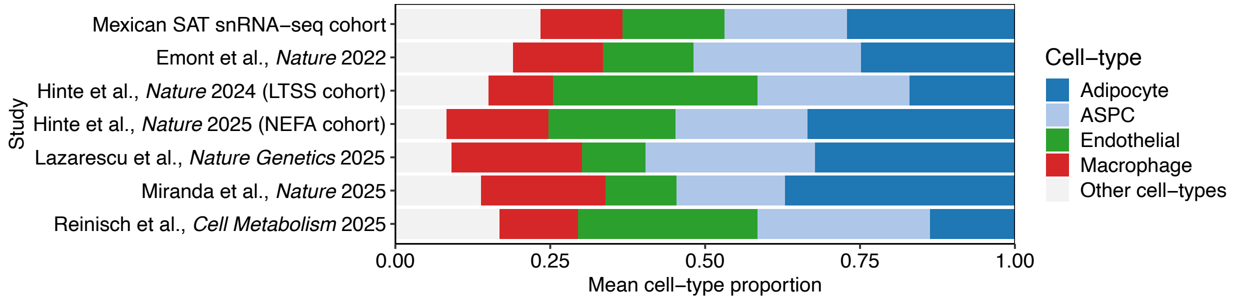

Fig. S4. The proportions in the Mexican SAT snRNA-seq cohort of the four main cell-types are comparable to those of previously published SAT snRNA-seq cohorts.

Stacked barplots compare the cellular compositions between the Mexican SAT snRNA-seq cohort and previously published SAT snRNA-seq datasets (Emont et al.[5], Lazarescu et al.[21], Reinisch et al.[23], Miranda et al.[22], and Hinte et al.[20], separating the latter by the LTSS and NEFA cohort) for the four main cell-types.

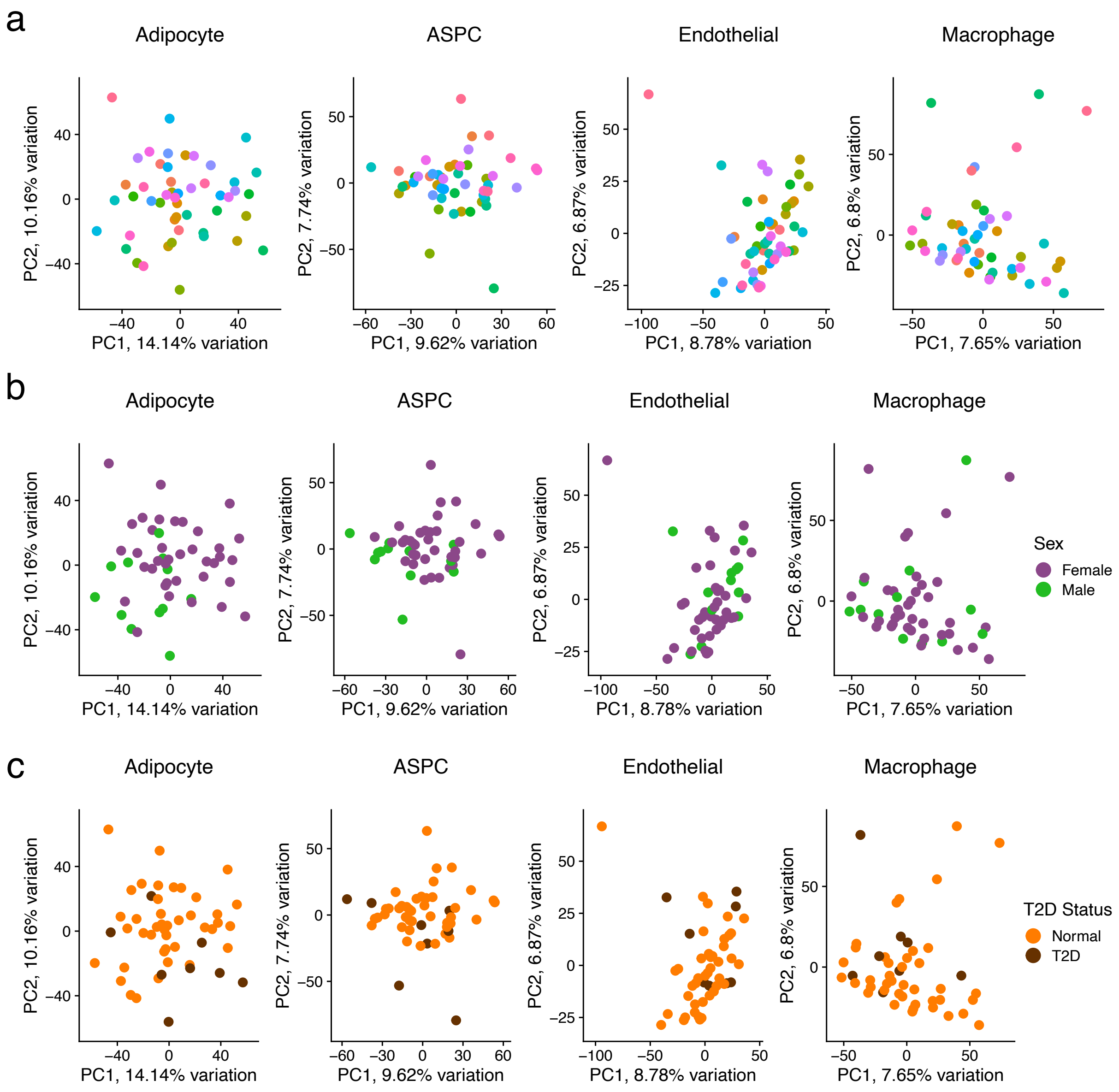

Fig. S5. Principal component analysis of cell-type level pseudobulk gene expression per sample.

(a-c) Biplots show the first two principal components (PC) of the pseudobulk gene expression, adjusted for the number of cell-type nuclei per sample, for each of the four main cell-types. Points are colored by a) sample, b) sex, and c) T2D status. The x and y axes indicate the variance in adjusted gene expression explained by the first and second PCs, respectively.

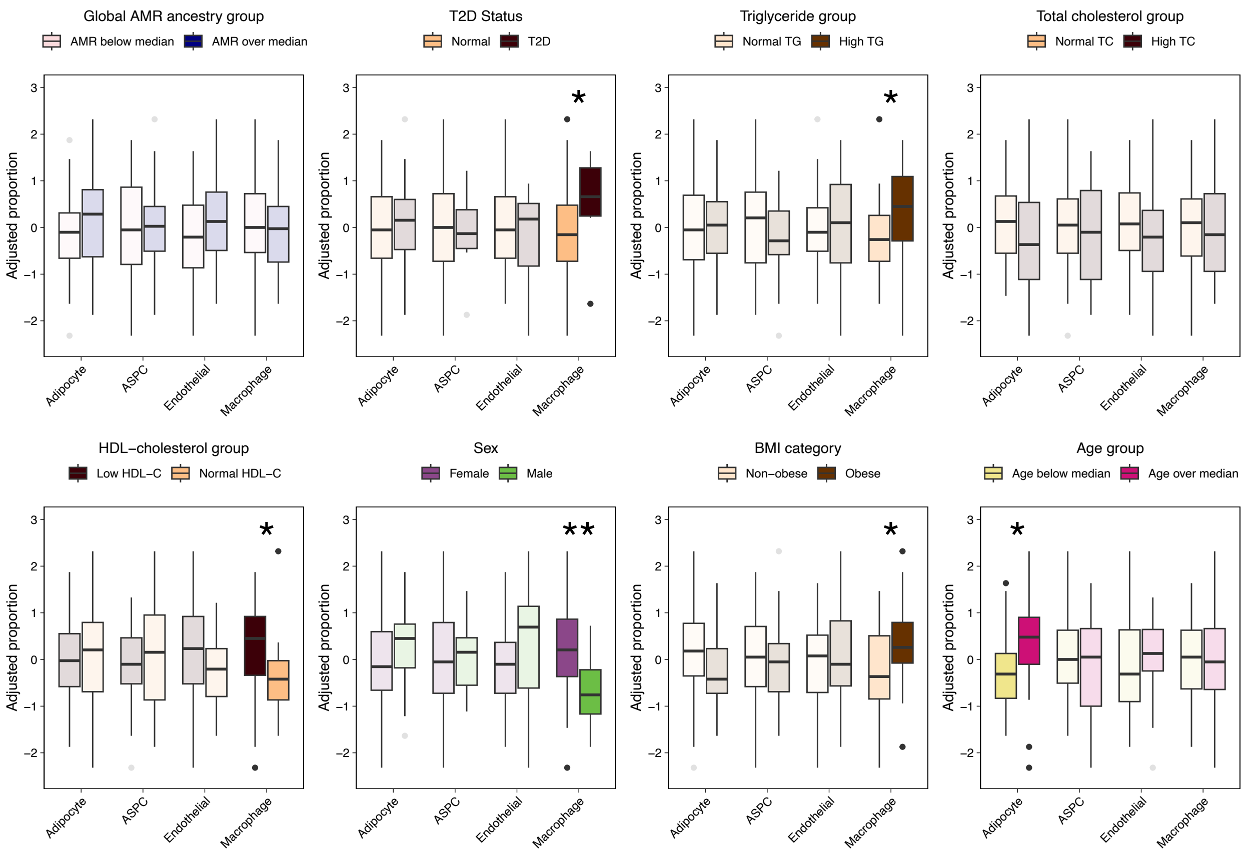

Fig. S6. Comparisons of the cell-type proportions derived from the single cell level data by context and CMD traits detect relatively minor differences.

Boxplots compare the cell-type proportions in the SAT snRNA-seq data of the 49 Mexican individuals by sex, BMI, global AMR ancestry, age, HDL-cholesterol (HDL-C), serum total cholesterol (TC), serum triglycerides (TGs) and the type 2 diabetes (T2D) status. The following groups were used: males and females; non-obese BMI (BMI<30) and obese (BMI≥30); lower global AMR ancestry (global AMR ancestry≤ median of 0.624) and higher global AMR ancestry (global AMR ancestry>0.624); lower age (age≤ median of 47 years) and higher age (age>47); low HDL-C (HDL-C<40 mg/dL for males, <50mg/dL for females) and normal HDL-C; normal TC (TC<200 mg/dL) and high TC (TC≥200); normal TGs (TGs<150mg/dL) and high TGs (TGs≥150); and T2D cases and controls. Cell-type proportions were adjusted for age, global AMR ancestry, sex, and BMI, omitting each covariate when it was the tested outcome. The box

limits indicate the first and third quartiles; whiskers of each box, 1.5 × the interquartile range (IQR) from the first and third quartiles; center line, median; and points, outliers. Significant differences in cell-type proportions between the two groups per context or CMD trait as assessed by a Wilcoxon test are shown in fully opaque colored boxes and denoted with asterisks, while nonsignificant comparisons are displayed with lighter (translucent) coloring. *P*-values are indicated as follows: *=0.049 (T2D), 0.045 (TGs), 0.012 (HDL-C), 0.036 (BMI), and 0.017 (Age group), **= 0.008, while non-significant *p*-values are not shown (see Additional file 3: Table S6 for all exact *p*-values).

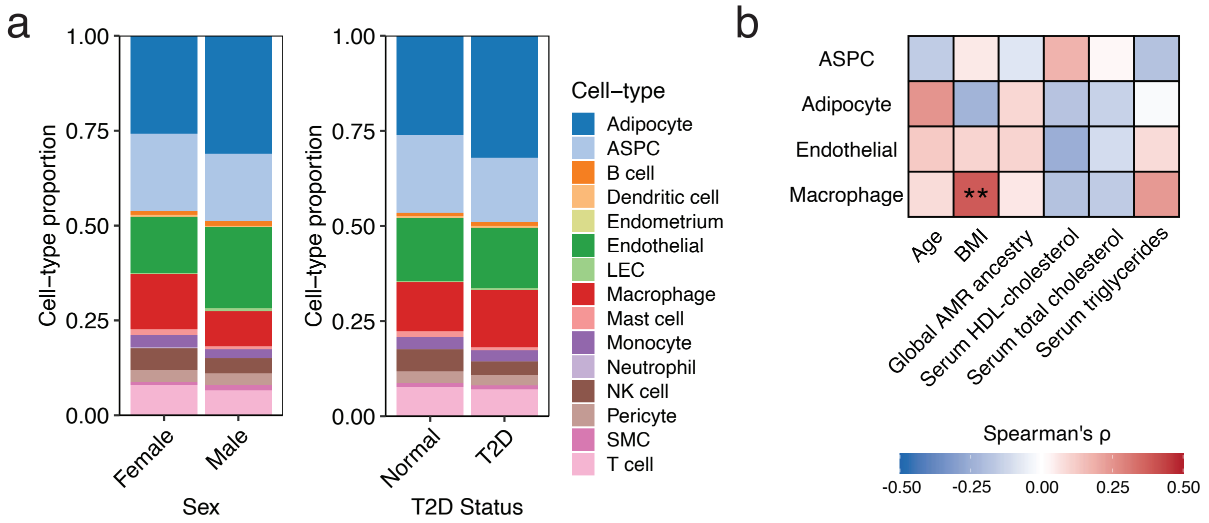

Fig. S7. The proportions of main cell-types and cellular subtypes differ by binary traits (sex and type 2 diabetes (T2D) status) and correlate with continuous contexts and CMD traits. **(a)** Stacked barplots compare the proportions of main cell-types between females and males (left) and individuals with and without T2D (right). The Wilcoxon *p*-values of these comparisons, after adjusting for the covariates of age, BMI, global admixed American ancestry, and sex, are provided in Additional file 3: Table S8. **(b)** Heatmaps display the pairwise Spearman's correlations between the continuous outcomes of age, BMI, global admixed American ancestry (AMR), serum high-density lipoprotein (HDL)-cholesterol, serum total cholesterol, and serum triglycerides and proportions of the main cell-types. Each tile is colored by the magnitude of the Spearman's correlation coefficient (ρ). Cell-type proportions were adjusted for age, global AMR ancestry, sex, and BMI, omitting each covariate when it was the tested outcome. Asterisks denote a nominally significant correlation. Significance thresholds for unadjusted *p*-values: *$p<0.05$, **$p<0.01$, ***$p<0.001$. Exact *p*-values for all correlations are listed in Additional file 3: Table S8.

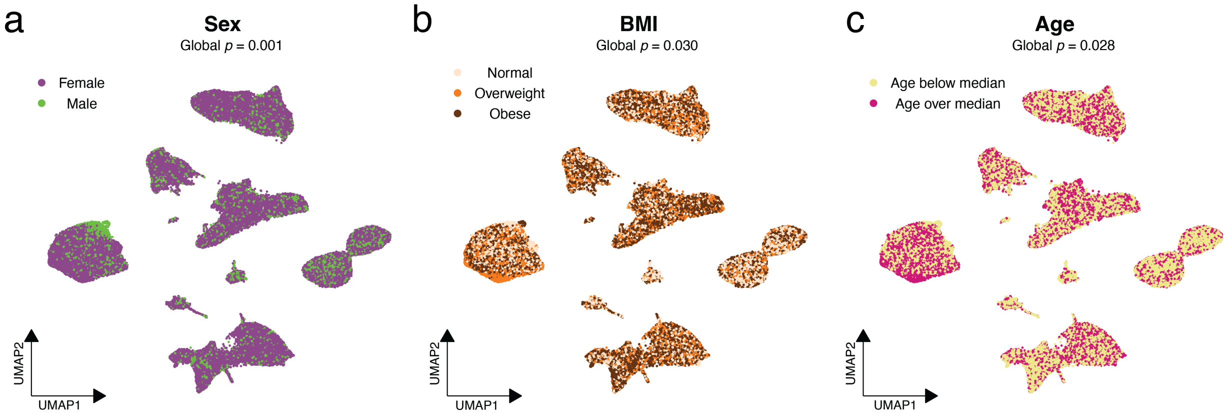

Fig. S8. The Mexican SAT snRNA-seq data shows statistically significant differences by sex, BMI, and age on the reduced dimension space.

(a-c) Uniform manifold approximation and projection (UMAP) visualizations of the 128,057 nuclei from 49 individuals are colored by a) sex, b) obesity category by BMI, and c) binarized age of the participant. We use the median to separate the lower and higher age groups and employ the obesity categories by BMI of normal weight (BMI<25), overweight (25≤BMI<30), and obese (BMI≥30). The subtitle reports the *p*-value of global transcriptomic heterogeneity by the context, derived using the CNA tool[77].

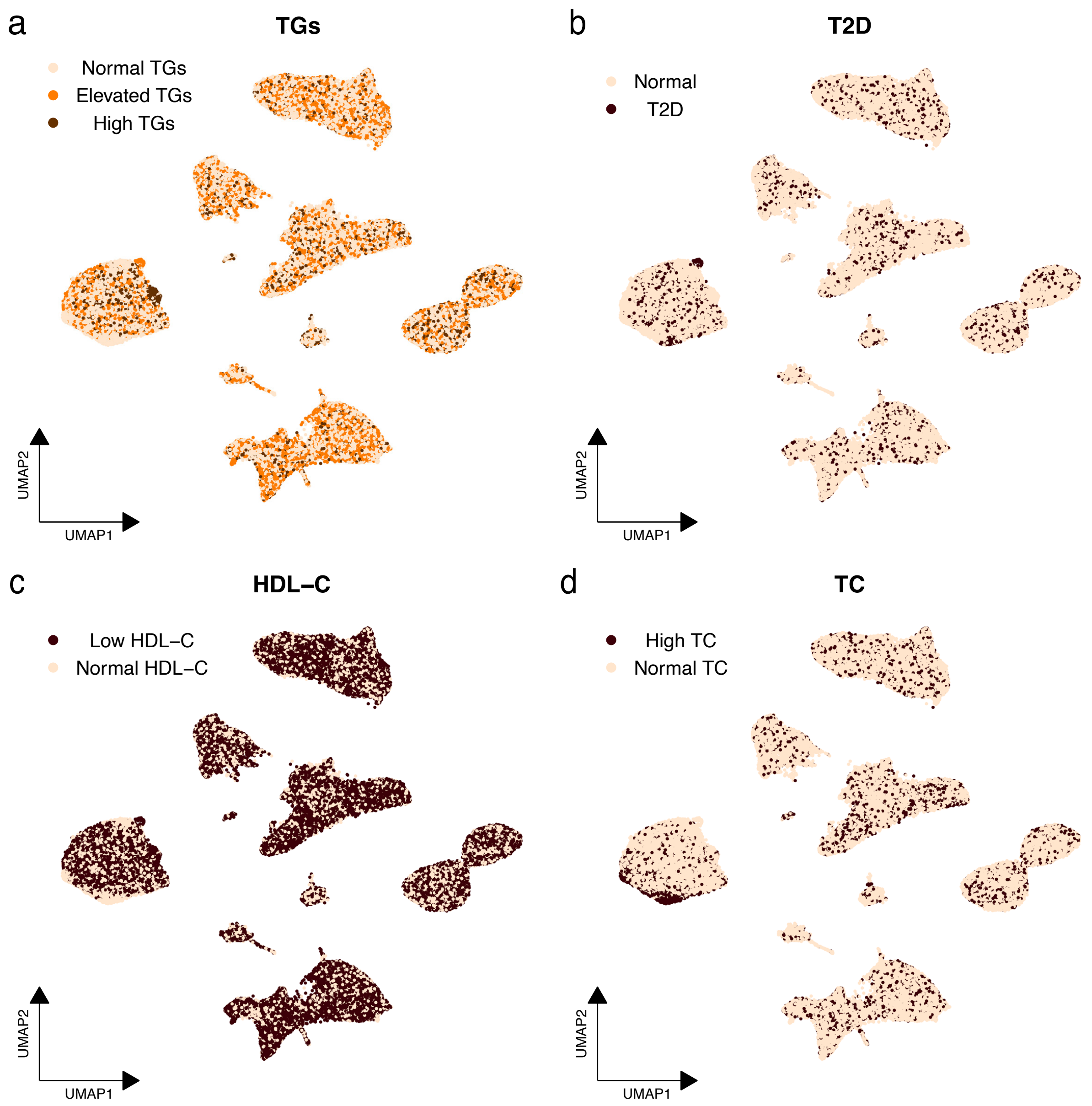

Fig. S9. Single nucleus RNA-sequencing data of SAT biopsies from 49 Mexican individuals shows differences in the UMAP space by the CMD traits of type 2 diabetes (T2D), serum triglycerides (TGs), serum total cholesterol (TC), and serum HDL-cholesterol (HDL-C). **(a-d)** UMAP visualizations of the 128,057 nuclei from the 49 individuals are colored by the a) TGs, b) T2D, c) HDL-C, and d) TC traits. We use the following groupings: a) normal TGs (TGs<150mg/dL), elevated TGs (150≤TGs<500), and very high TGs (TGs≥500); T2D case and

control; c) low HDL-C (HDL-C<40 mg/dL for males, <50mg/dL for females) and normal HDL-C; and d) normal TC (TC<200 mg/dL) and high TC (TC≥200).

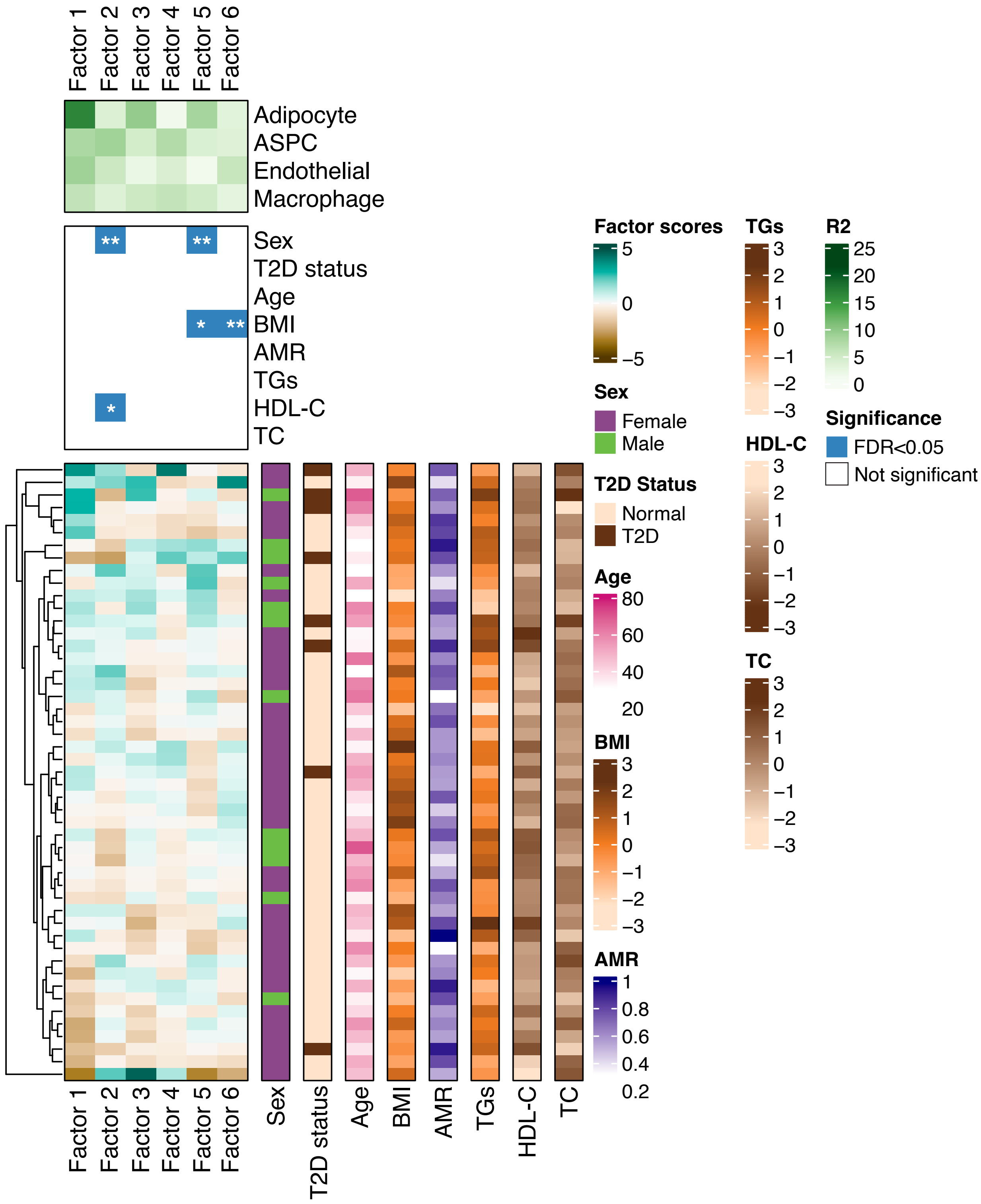

Fig. S10. Multi-cellular factor analysis (MOFAcell)[78] reveals that particularly adipocytes display strong cellular heterogeneity, with sex and BMI influencing their variability.

Heatmaps summarize the associations of the latent factor scores from the multi-cellular factor analysis with contexts and CMD conditions, sample level embeddings, and explained variance of gene expression for each cell-type recovered by each factor. The top panel shows the variance of expression explained (R^2) in each cell-type by each latent factor score. The middle row depicts the significant (FDR<0.05) associations between the latent factor scores and sex, BMI, global AMR ancestry, age, serum HDL-cholesterol (HDL-C), serum total cholesterol (TC), serum triglycerides (TGs) and the type 2 diabetes (T2D) status. The bottom panel depicts the hierarchical clustering of the latent factor scores as inferred by the model alongside the tested contexts and CMD traits of each individual. Significance thresholds for Benjamini-Hochberg adjusted *p*-values (FDR): *$p<0.05$, **$p<0.01$. Exact *p*-values for the associations are listed in Additional file 3: Table S10.

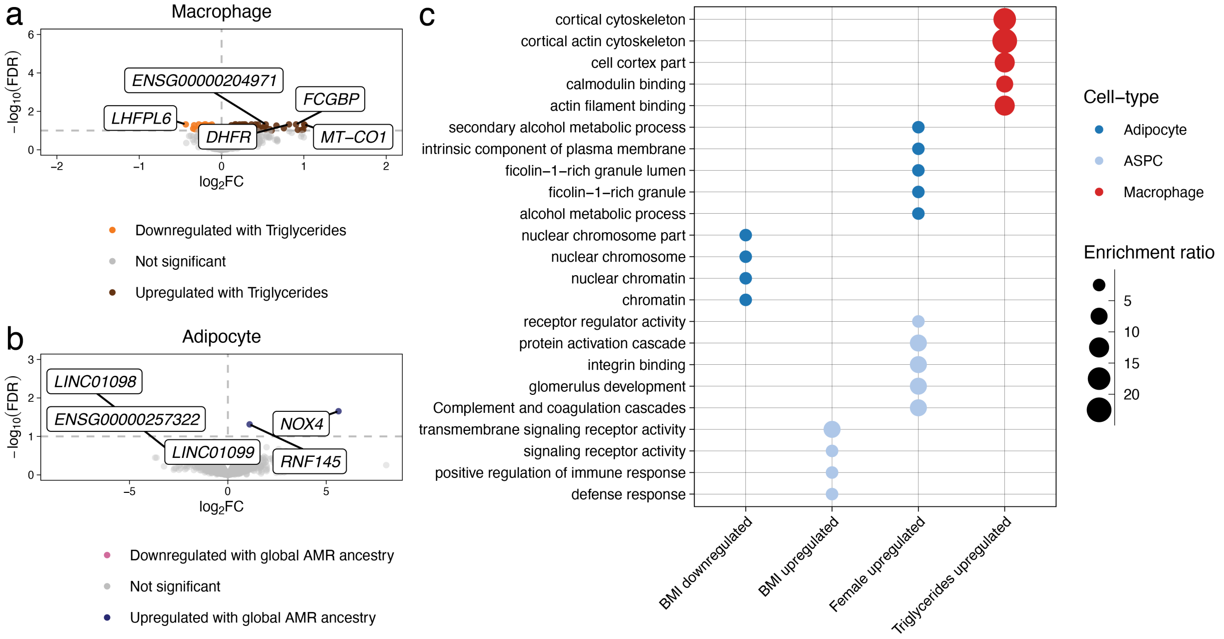

Fig. S11. Ancestry and total serum triglycerides affect gene expression in adipocytes and macrophages, respectively, and the differentially expressed genes by BMI, sex, and triglycerides show functional enrichments.

(a-b) The differential expression (DE) patterns in a) macrophages by triglycerides and b) adipocytes by the estimated global admixed American ancestry (AMR) are depicted in volcano plots. We plot each gene by the $-\log_{10}$ (FDR) and $\log_2$fold change in cell-type level expression, color points by the direction of DE as well as the significance, and label the top five most significantly DE genes per outcome and cell-type.

(c) Dot plots indicate the top five most highly enriched pathways among the significantly overrepresented pathways for each of the cell-type level DE gene sets, where the dot size indicates the enrichment ratio. Exact *p*-values of the enrichments are listed in Additional file 3: Table S12.

.

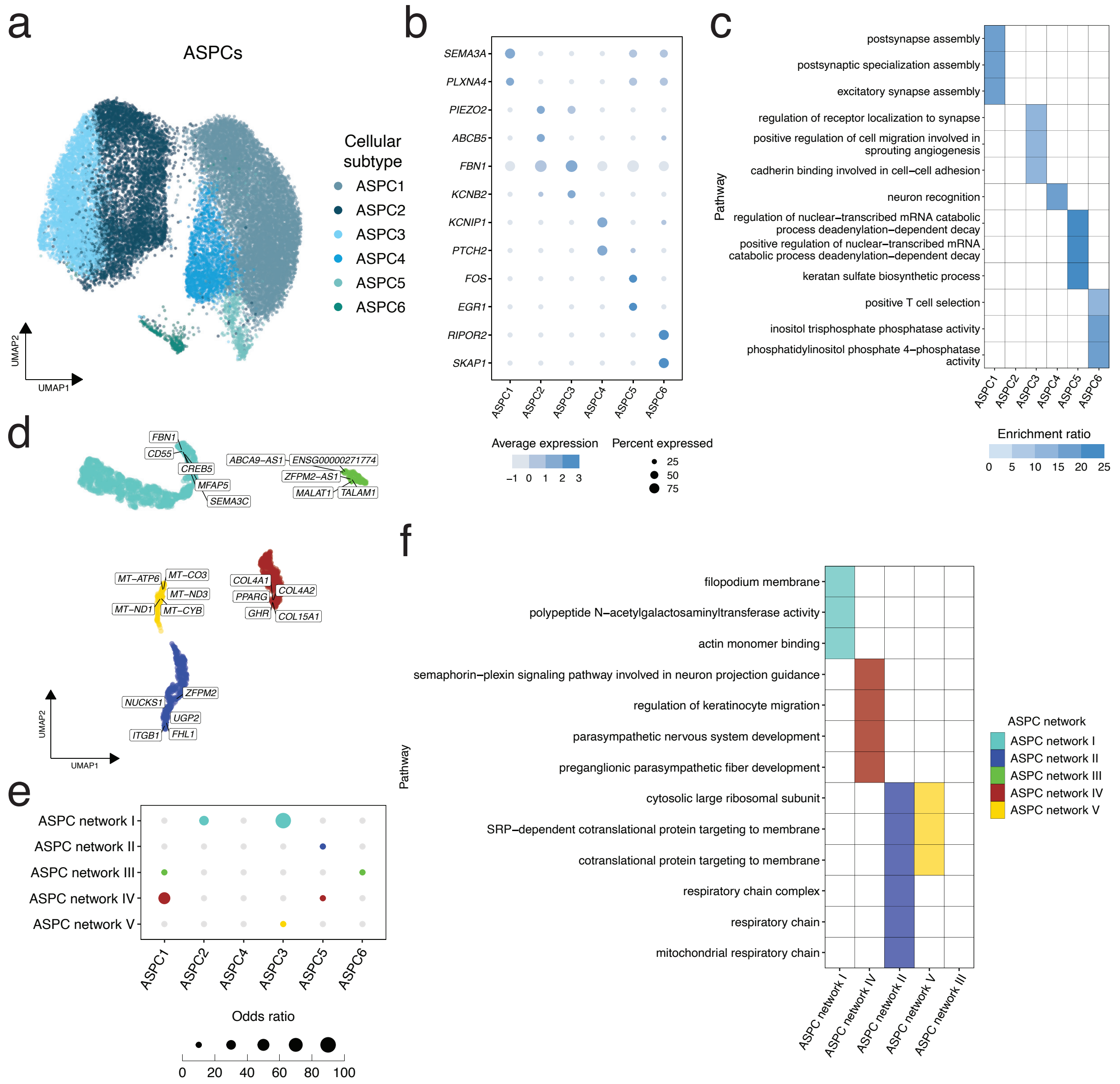

Fig. S12. Adipose stem and precursor cells (ASPCs) contain functionally distinct subtypes and cell-type level co-expression networks.

(a) UMAP visualization of the 24,477 nuclei broadly classified as ASPCs, colored by subtypes. **(b)** Dot plots compare the average scaled expression of the two unique ASPC marker genes with the highest $\log_2$fold change per ASPC subtype across the six ASPC subtypes. The size of each

point represents the percentage of nuclei expressing the y-axis gene in the x-axis subtype, and the color intensity depicts the average expression of the gene in the subtype.

(c) A heatmap visualizes the three most significantly (FDR<0.05) enriched functional pathways for the unique marker genes of each ASPC subtype. We shade the tiles on the plot by significance of enrichment and enrichment ratio of the pathway for subtype marker genes, with a darker shading indicating a larger enrichment ratio and sets without significant enrichment shown as white.

(d) The top 25 most connected "hub" genes of each ASPC co-expression network, i.e., the genes with the highest intra-modular connectivity score based on the correlation of expression values with the module eigengene (kME), are visualized on a supervised UMAP space. We label the five genes with the highest connectivity score (kME) per network.

(e) Dot plots show the correspondence between the ASPC subtype unique marker genes and network genes. Each point is colored by significance (FDR<0.05) of enrichment between the subtype unique marker genes and network genes, where the size represents the odds ratio of the overlap, and non-significant overlaps are depicted as light grey points.

(f) A heatmap compares the top three significant pathways with the largest enrichment ratio per ASPC network. Filled tiles indicate that the genes in the ASPC networks from the x-axis are significantly (FDR<0.05) enriched for the functional pathway on the y-axis, colored by network.

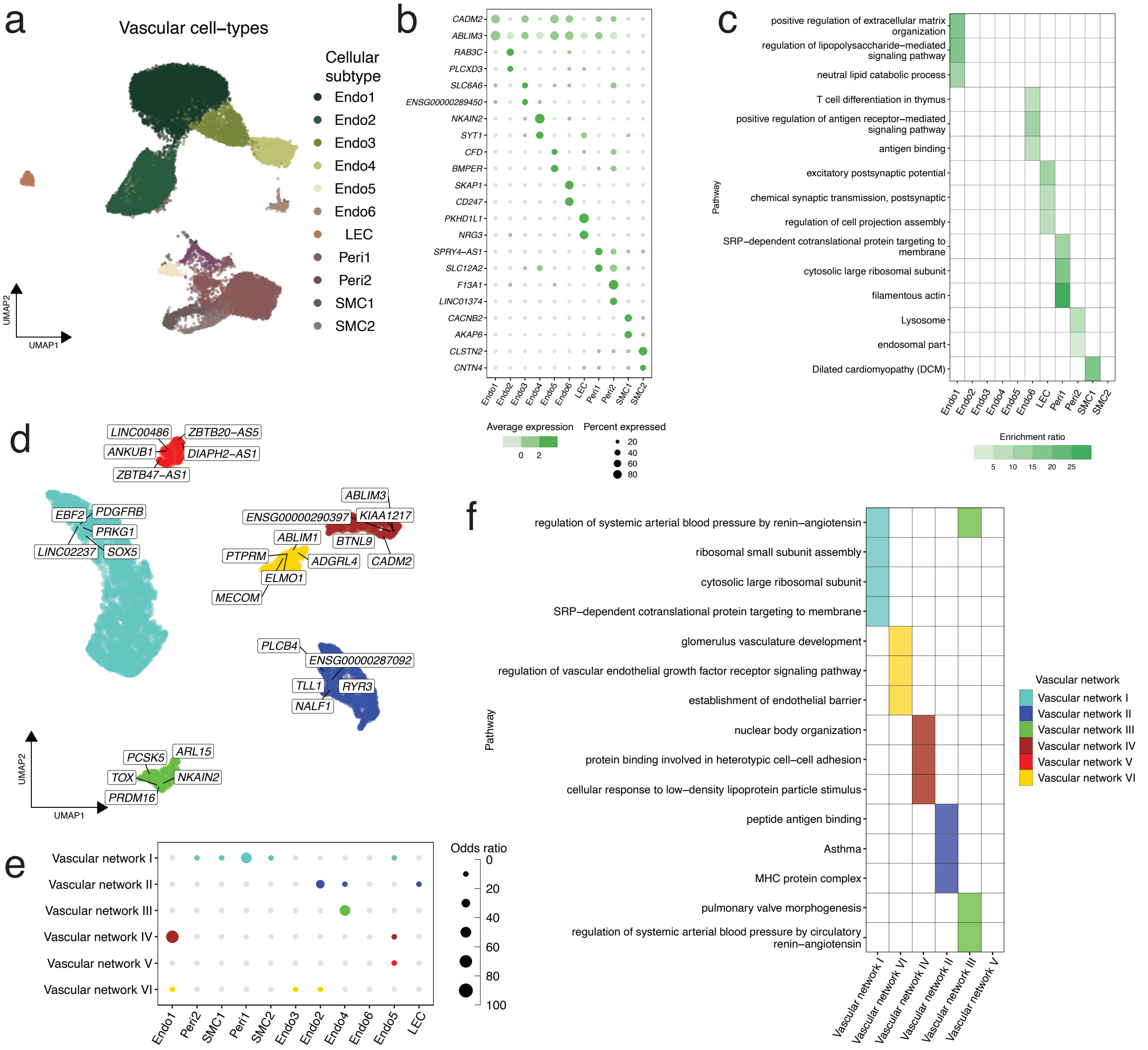

Fig. S13. Subtypes and cell-type level co-expression networks within the vascular cell-types.

(a) UMAP visualization of the 26,363 nuclei broadly classified as vascular cell-types, colored by subtypes.

(b) Dot plots compare the average scaled expression of the two unique marker genes with the highest $\log_2$fold change per vascular cell-type subtype across the 11 subtypes. The size of each point represents the percentage of nuclei expressing the y-axis gene in the x-axis subtype, and the color intensity depicts the average expression of the gene in the subtype.

(c) A heatmap visualizes the three most significantly (FDR<0.05) enriched functional pathways for the unique marker genes of each vascular subtype. We shade the tiles on the plot by significance of enrichment and enrichment ratio of the pathway for subtype marker genes, with a darker shading indicating a larger enrichment ratio and sets without significant enrichment shown as white.

(d) The top 25 most connected "hub" genes of each vascular network, i.e., the genes with the highest intra-modular connectivity score based on the correlation of expression values with the module eigengene (kME), are visualized on a supervised UMAP space. We label the five genes with the highest connectivity score (kME) per network.

(e) Dot plots show the correspondence between the vascular subtype unique marker genes and network genes. Each point is colored by significance (FDR<0.05) of enrichment between the subtype unique marker genes and network genes, where the size represents the odds ratio of the overlap, and non-significant overlaps are depicted as light grey points.

(f) A heatmap compares the top three significant pathways with the largest enrichment ratio per vascular network. Filled tiles indicate that the genes in the vascular networks from the x-axis are significantly (FDR<0.05) enriched for the functional pathway on the y-axis, colored by network.

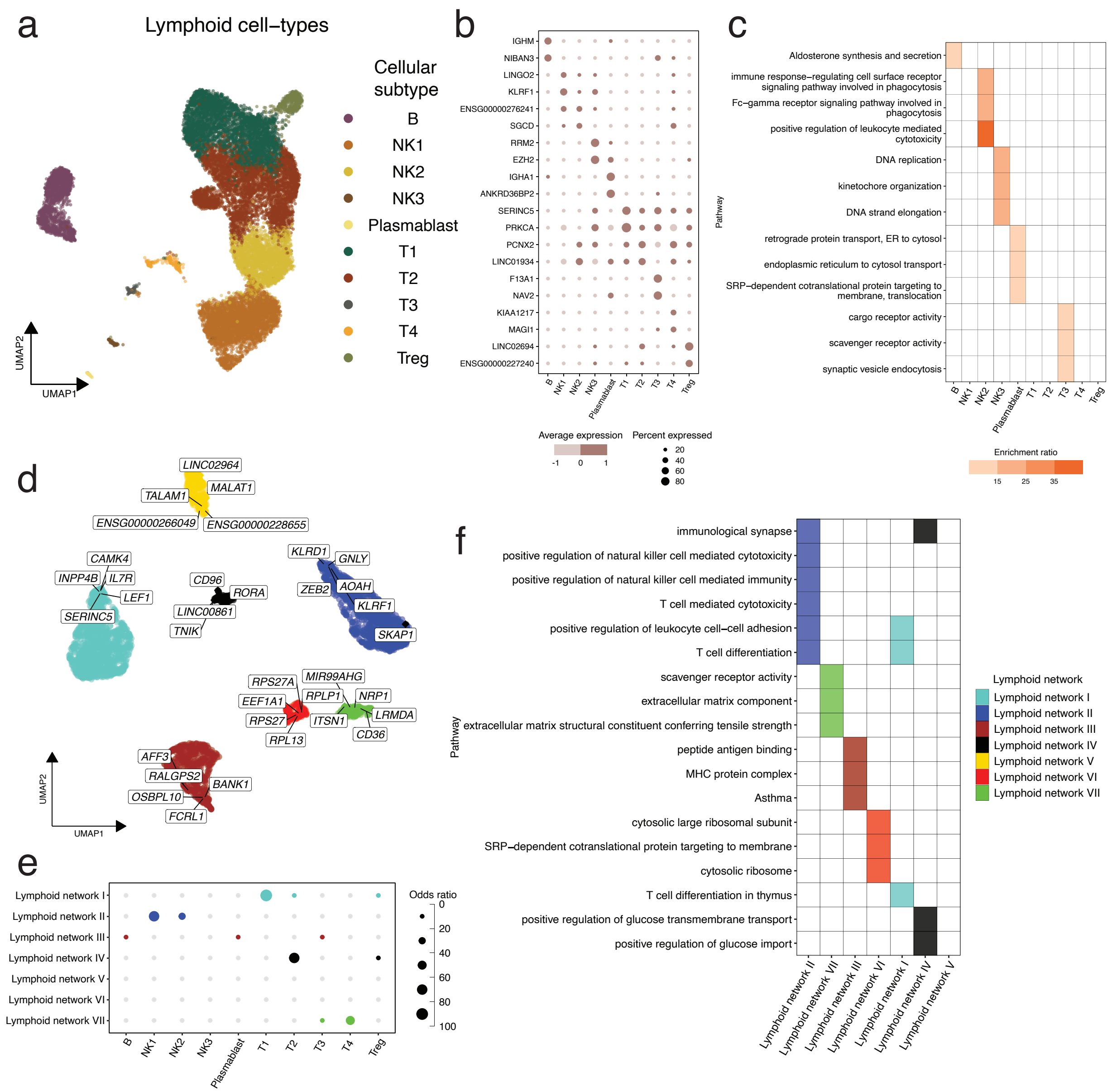

Fig. S14. The subtypes and cell-type level co-expression networks within the lymphoid cell-types capture distinct subtype functions.

(a) UMAP visualization of the 18,401 nuclei broadly classified as lymphoid cell-types, colored by subtypes.

(b) Dot plots compare the average scaled expression of the two unique marker genes with the highest $\log_2$fold change per lymphoid subtype across the 10 lymphoid subtypes. The size of each

point represents the percentage of nuclei expressing the y-axis gene in the x-axis subtype, and the color intensity depicts the average expression of the gene in the subtype.

(c) A heatmap visualizes the three most significantly (FDR<0.05) enriched functional pathways for the unique marker genes of each lymphoid subtype. We shade the tiles on the plot by significance of enrichment and enrichment ratio of the pathway for subtype marker genes, with a darker shading indicating a larger enrichment ratio and sets without significant enrichment shown as white.

(d) The top 25 most connected "hub" genes of each lymphoid network, i.e., the genes with the highest intra-modular connectivity score based on the correlation of expression values with the module eigengene (kME), are visualized on a supervised UMAP space. We label the five genes with the highest connectivity score (kME) per network.

(e) Dot plots show the correspondence between the lymphoid subtype unique marker genes and network genes. Each point is colored by significance (FDR<0.05) of enrichment between the subtype unique marker genes and network genes, where the size represents the odds ratio of the overlap, and non-significant overlaps are depicted as light grey points.

(f) A heatmap compares the top three significant pathways with the largest enrichment ratio per lymphoid network. Filled tiles indicate that the genes in the lymphoid networks from the x-axis are significantly (FDR<0.05) enriched for the functional pathway on the y-axis, colored by network.

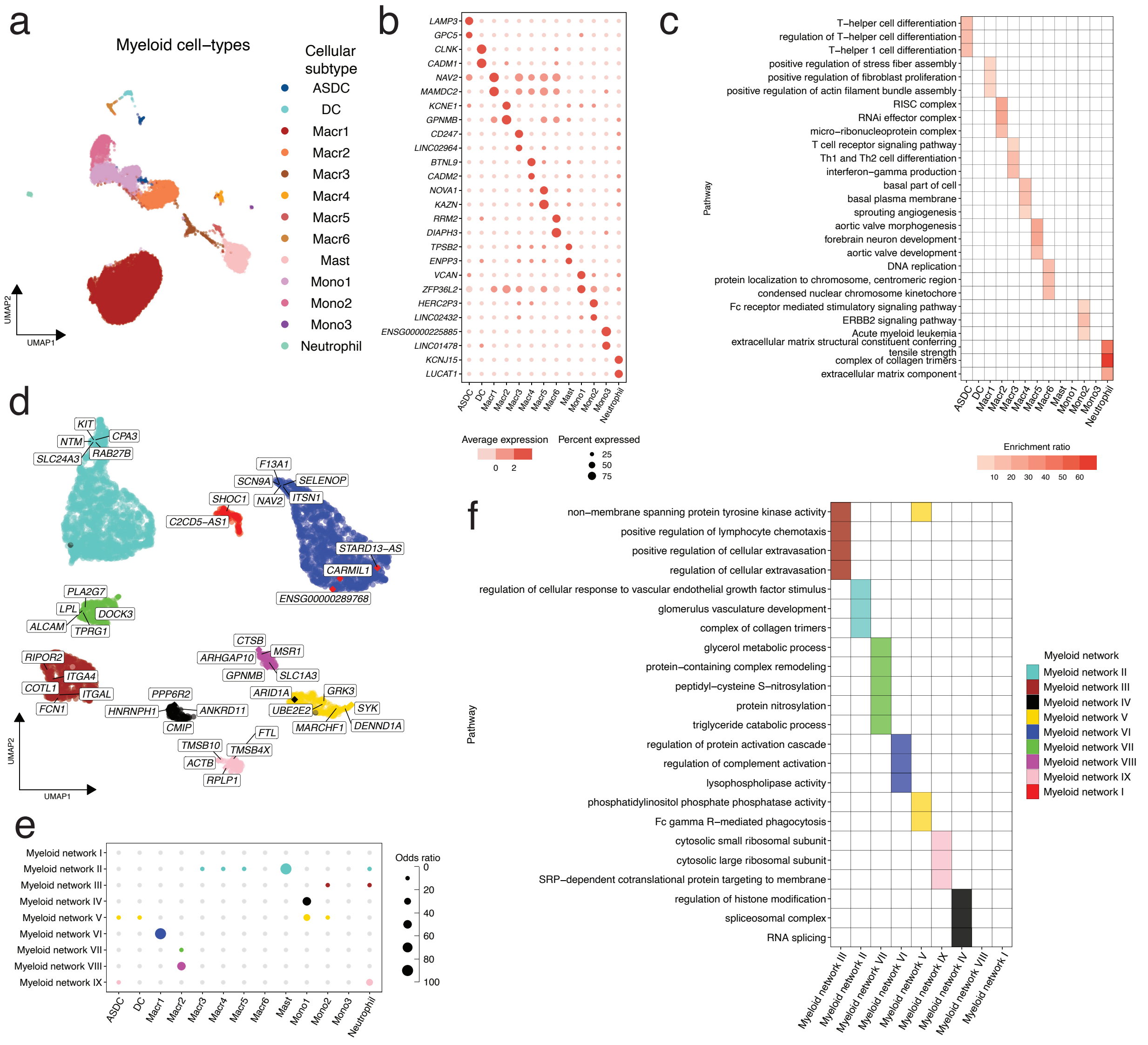

Fig. S15. Subtypes and cell-type level co-expression networks within the myeloid cell-types.

(a) UMAP visualization of the 24,318 nuclei broadly classified as myeloid cell-types, colored by subtypes.

(b) Dot plots compare the average scaled expression of the two unique marker genes with the highest $\log_2$fold change per myeloid subtype across the 13 myeloid subtypes. The size of each point represents the percentage of nuclei expressing the y-axis gene in the x-axis subtype, and the color intensity depicts the average expression of the gene in the subtype.

(c) A heatmap visualizes the three most significantly (FDR<0.05) enriched functional pathways for the unique marker genes of each myeloid subtype. We shade the tiles on the plot by significance of enrichment and enrichment ratio of the pathway for subtype marker genes, with a darker shading indicating a larger enrichment ratio and sets without significant enrichment shown as white.

(d) The top 25 most connected "hub" genes of each myeloid network, i.e., the genes with the highest intra-modular connectivity score based on the correlation of expression values with the module eigengene (kME), are visualized on a supervised UMAP space. We label the five genes with the highest kME per network.

(e) Dot plots show the correspondence between the myeloid subtype unique marker genes and network genes. Each point is colored by significance (FDR<0.05) of enrichment between the subtype unique marker genes and network genes, where the size represents the odds ratio of the overlap, and non-significant overlaps are depicted as light grey points.

(f) A heatmap compares the top three significant pathways with the largest enrichment ratio per myeloid network. Filled tiles indicate that the genes in the myeloid networks from the x-axis are significantly (FDR<0.05) enriched for the functional pathway on the y-axis, colored by network.

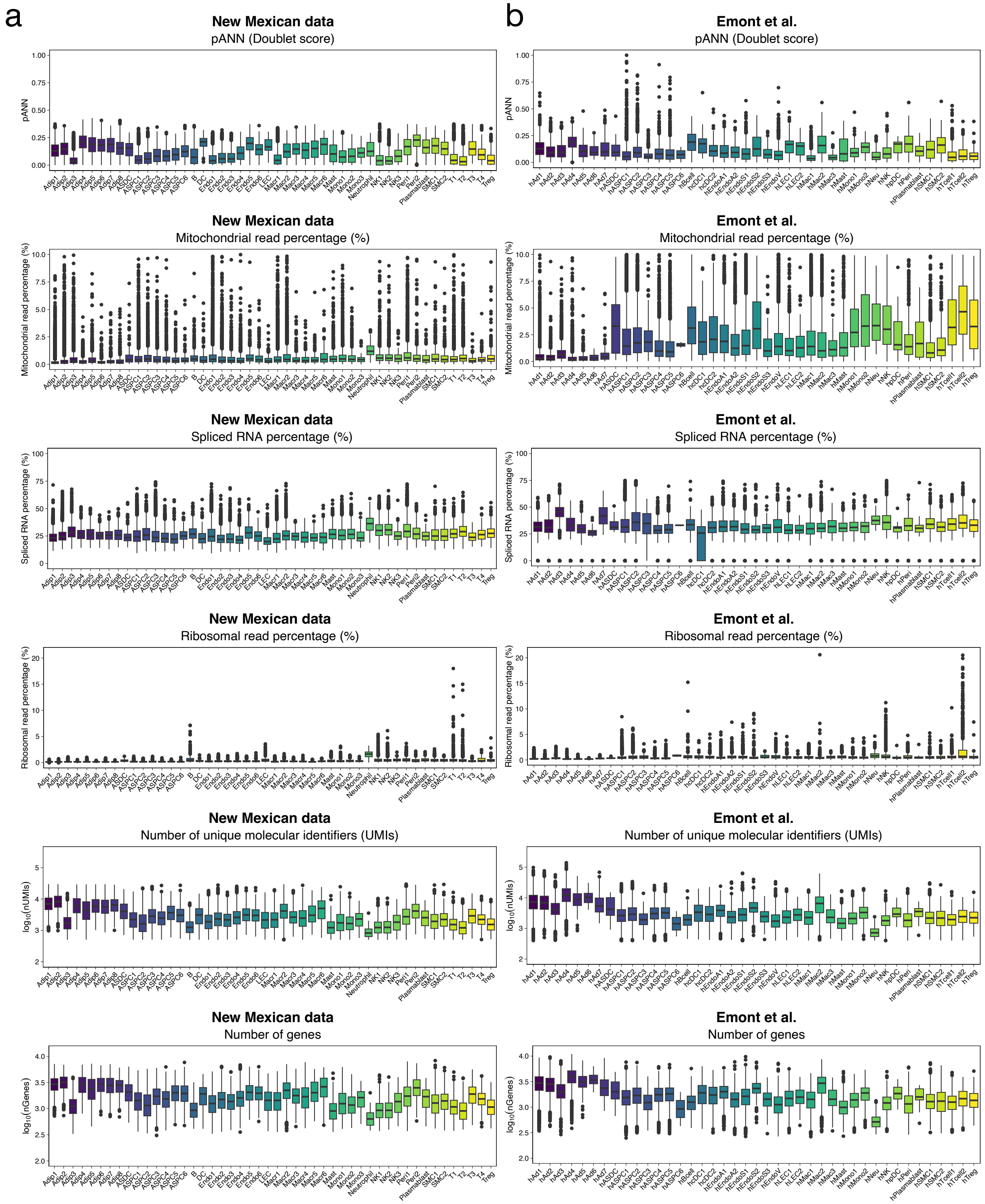

Fig. S16. Quality of the Mexican SAT snRNA-seq data compared to that of the SAT snRNA-seq of a previously published adipose single cell atlas[5,66].

(a-b) Boxplots depict the distributions of the pANN (doublet score), mitochondrial read percentage, spliced RNA percentage, ribosomal read percentage, number of unique molecular identifiers, and number of genes per subtype in a) the Mexican SAT snRNA-seq data (n=49) and b) the SAT snRNA-seq data (n=13) from the previous adipose single cell atlas[5,65]. The box limits indicate the first and third quartiles; whiskers of each box, 1.5 × the interquartile range (IQR) from the first and third quartiles; center line, median; and points, outliers. Abbreviations for the cell-types and subtypes are as follows: ASDC indicates AXL+SIGLEC6+ dendritic cells; ASPC adipose stem and precursor cells; DC dendritic cells; Endo endothelial cells; LEC lymphatic endothelial cells; Macr macrophages; Mono monocytes; NK natural killer cells; Peri pericytes; SMC smooth muscle cells; and Treg T-regulatory cells.

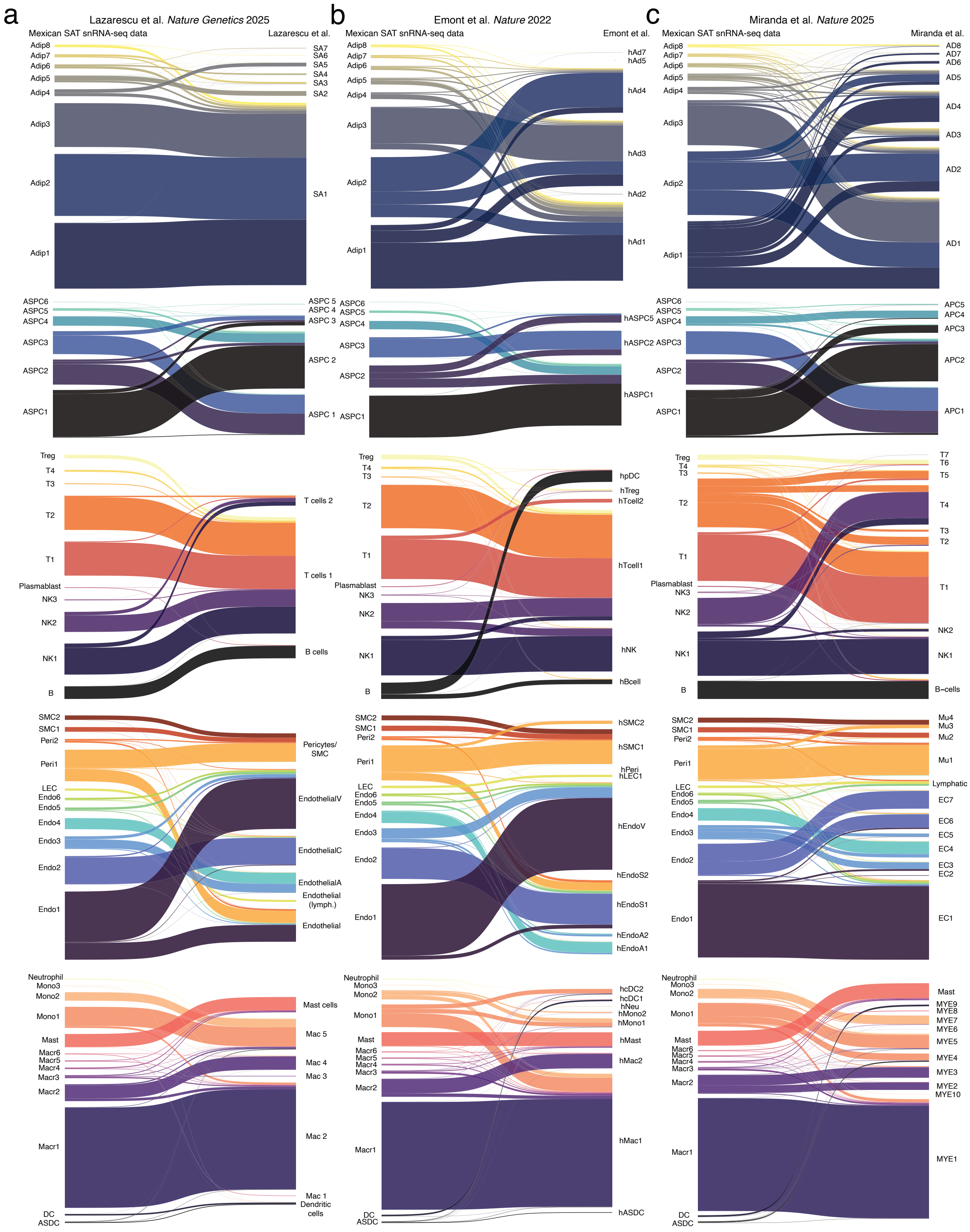

Fig. S17. Comparisons of the identified subtypes per cell-type with SAT subtype annotations from published atlases[5,21,22,66,72,73].

(a-c) Riverplots show the correspondence between the subtype labels of adipocytes, ASPCs, vascular cells, myeloid cells, and lymphoid cells in this study and the subtype annotations of our data using the annotations by the a) Lazarescu et al. study[21,73], b) Emont et al. study[5,66], and c) Miranda et al. study[22,72]. The left labels (per plot group) show nuclei by the subtype annotations in this study, and the right labels contain the annotations from the external study, where each line represents an individual nucleus from the Mexican SAT snRNA-seq data and is colored by the annotation from this study.

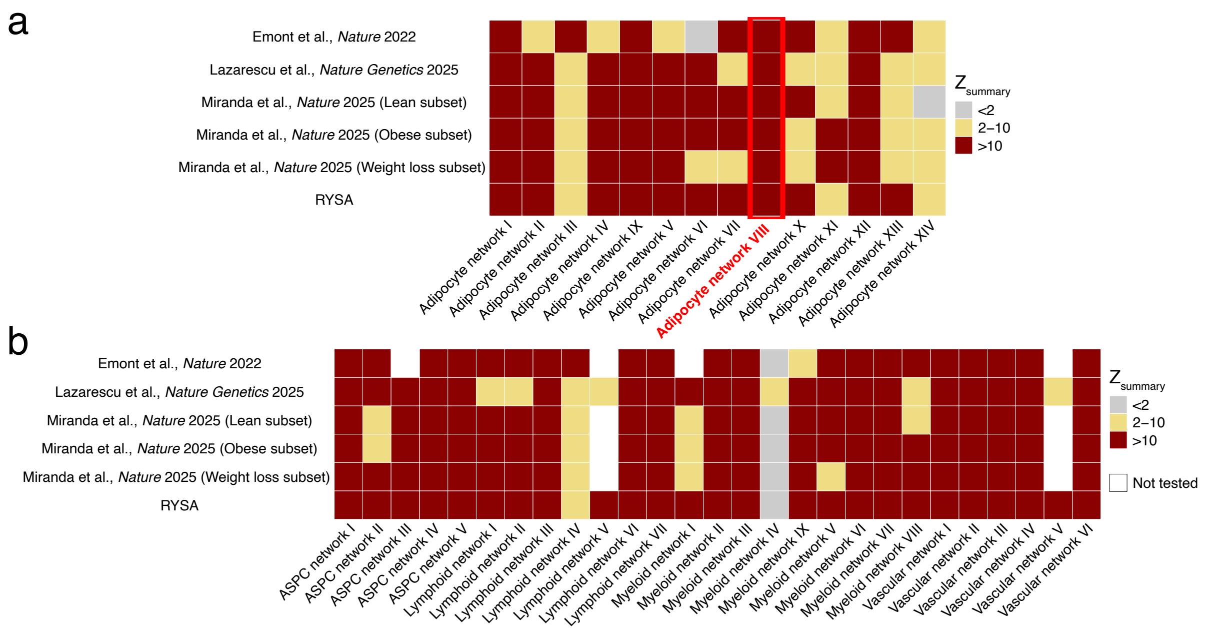

Fig. S18. The SAT cell-type level co-expression networks are highly preserved consistently across six independent external datasets.

(a-b) The preservations of a) adipocyte and b) ASPC, lymphoid, myeloid, and vascular cell-type level co-expression networks in six independent external SAT snRNA-seq datasets (Miranda et al. separately by condition (lean, obese, and weight loss)[22,72], Lazarescu et al.[21,73], Emont et al.[5,66], and the Finnish RYSA SAT snRNA-seq dataset[37,84]) based on the $Z_{summary}$ preservation statistic are shown in heatmaps. Moderate preservations, defined as $2< Z_{summary}<10$[85], are indicated in light yellow tiles, while high preservations, defined as $Z_{summary} >10$[85], are shown as dark red tiles. A grey tile indicates the network was not preserved in the external dataset, and white indicates that the network was not tested for preservation due to a high number of missing genes. The discovered sex-associated Adipocyte network VIII, boxed in this figure and bolded and colored red on the x-axis, is highly preserved across all of the external cohorts.

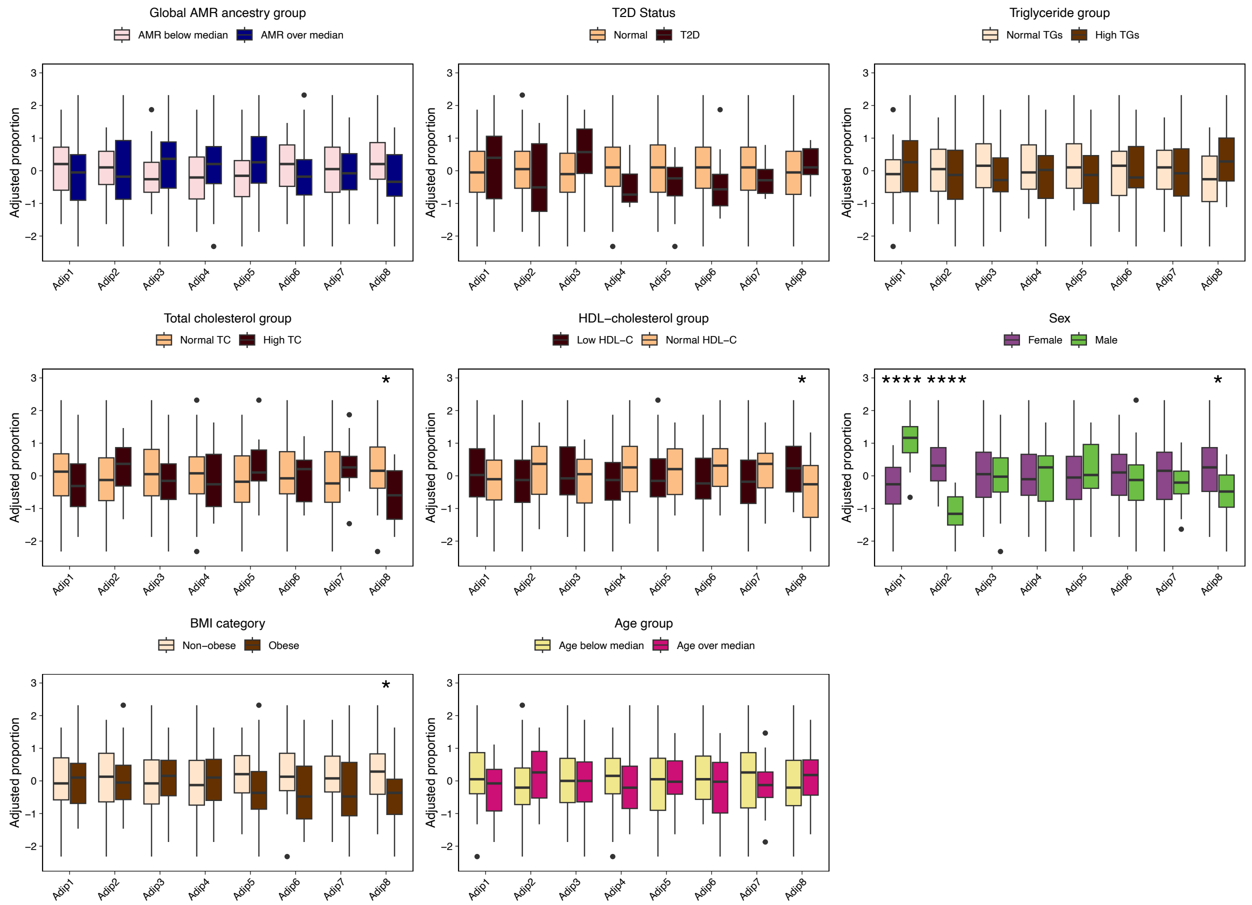

Fig. S19. Comparisons of the adipocyte subtype proportions by context and CMD traits detect sex differences among two adipocyte subtypes.

Boxplots compare the adipocyte subtype proportions in the SAT snRNA-seq data of the 49 Mexican individuals by sex, BMI, global admixed American (AMR) ancestry, age, serum HDL-cholesterol (HDL-C), serum total cholesterol (TC), serum triglycerides (TGs), and the type 2 diabetes (T2D) status. The following groups were used: males and females; non-obese BMI (BMI<30) and obese (BMI≥30); lower global AMR ancestry (global AMR ancestry≤ median of 0.624) and higher global AMR ancestry (global AMR ancestry>0.624); lower age (age≤ median of 47 years) and higher age (age>47); low HDL-C (HDL-C<40 mg/dL for males, <50mg/dL for females) and normal HDL-C; normal TC (TC<200 mg/dL) and high TC (TC≥200); normal TGs (TGs<150mg/dL) and high TGs (TGs≥150); and T2D cases and controls. Subtype proportions

were adjusted for age, global AMR ancestry, sex, and BMI, omitting each covariate when it was the tested outcome. The box limits indicate the first and third quartiles; whiskers of each box, 1.5 × the interquartile range (IQR) from the first and third quartiles; center line, median; and points, outliers. Asterisks denote a nominally significant difference in subtype proportions between the two groups per context or CMD trait as assessed by a Wilcoxon test. Significance thresholds for unadjusted *p*-values: *$p<0.05$, **$p<0.01$, ***$p<0.001$, and ****$p<0.0001$. Exact *p*-values for all comparisons are listed in Additional file 4: Table S18.

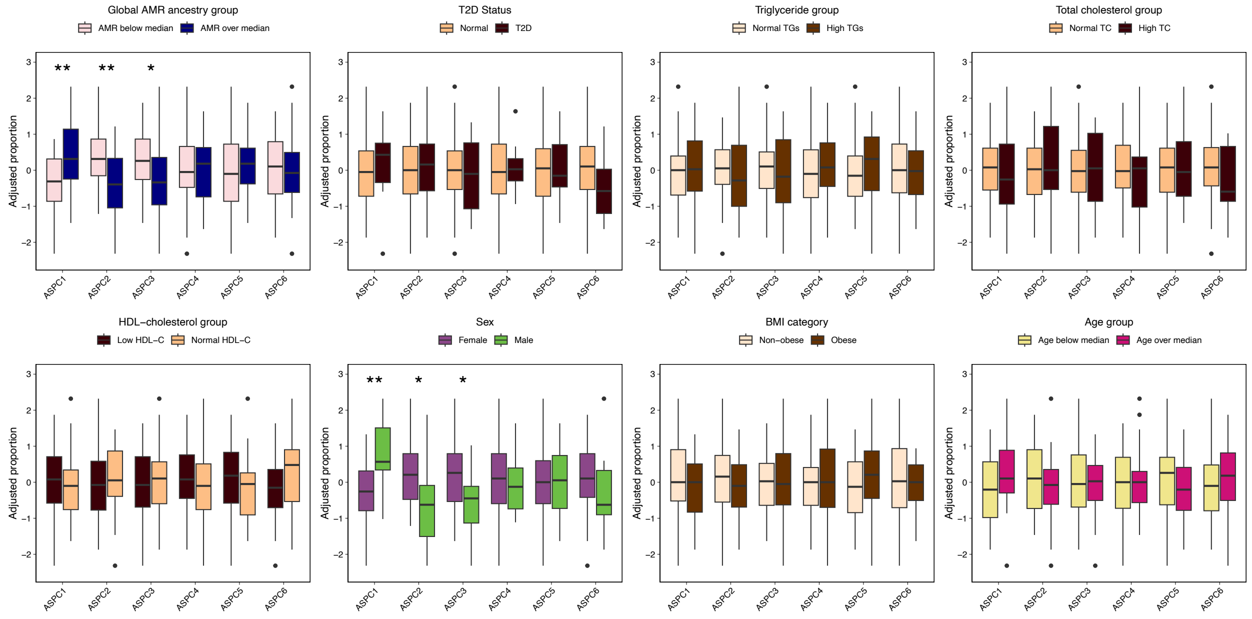

Fig. S20. The ASPC subtype proportions exhibit minor differences by CMD context and CMD trait.

Boxplots compare the ASPC subtype proportions in the SAT snRNA-seq data of the 49 Mexican individuals by sex, BMI, global admixed American (AMR) ancestry, age, serum HDL-cholesterol (HDL-C), serum total cholesterol (TC), serum triglycerides (TGs), and the type 2 diabetes (T2D) status. The following groups were used: males and females; non-obese BMI (BMI<30) and obese (BMI≥30); lower global AMR ancestry (global AMR ancestry≤ median of 0.624) and higher global AMR ancestry (global AMR ancestry>0.624); lower age (age≤ median of 47 years) and higher age (age>47); low HDL-C (HDL-C<40 mg/dL for males, <50mg/dL for females) and normal HDL-C; normal TC (TC<200 mg/dL) and high TC (TC≥200); normal TGs (TGs<150mg/dL) and high TGs (TGs≥150); and T2D cases and controls. Subtype proportions were adjusted for age, global AMR ancestry, sex, and BMI, omitting each covariate when it was the tested outcome. The box limits indicate the first and third quartiles; whiskers of each box, 1.5 × the interquartile range (IQR) from the first and third quartiles; center line, median; and points, outliers. Asterisks denote a significant difference in Subtype proportions between the two

groups per context or CMD trait as assessed by a Wilcoxon test. Significance thresholds for unadjusted *p*-values: *p<0.05, **p<0.01, ***p<0.001, and ****p<0.0001. Exact *p*-values for all comparisons are listed in Additional file 4: Table S18.

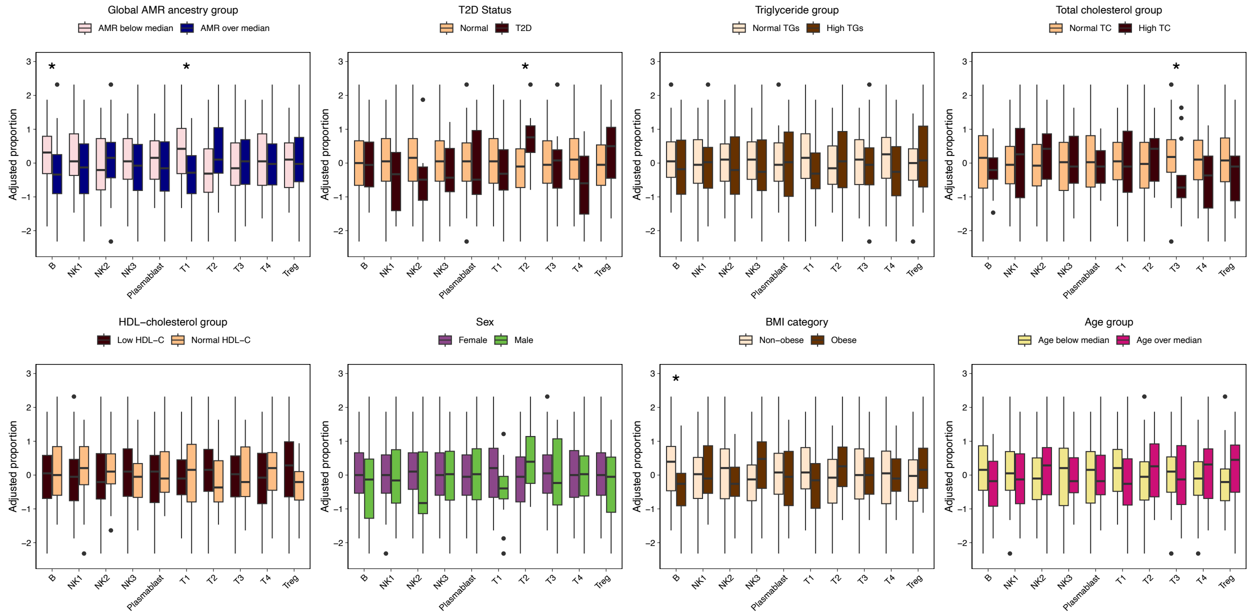

Fig. S21. Proportions of the lymphoid subtypes show minor differences by CMD context and CMD trait.

Boxplots compare the lymphoid subtype proportions in the SAT snRNA-seq data of the 49 Mexican individuals by sex, BMI, global admixed American (AMR) ancestry, age, serum HDL-cholesterol (HDL-C), serum total cholesterol (TC), serum triglycerides (TGs), and the type 2 diabetes (T2D) status. The following groups were used: males and females; non-obese BMI (BMI<30) and obese (BMI≥30); lower global AMR ancestry (global AMR ancestry≤ median of 0.624) and higher global AMR ancestry (global AMR ancestry>0.624); lower age (age≤ median of 47 years) and higher age (age>47); low HDL-C (HDL-C<40 mg/dL for males, <50mg/dL for females) and normal HDL-C; normal TC (TC<200 mg/dL) and high TC (TC≥200); normal TGs (TGs<150mg/dL) and high TGs (TGs≥150); and T2D cases and controls. Subtype proportions were adjusted for age, global AMR ancestry, sex, and BMI, omitting each covariate when it was the tested outcome. The box limits indicate the first and third quartiles; whiskers of each box, 1.5 × the interquartile range (IQR) from the first and third quartiles; center line, median; and points, outliers. Asterisks denote a significant difference in Subtype proportions between the two

groups per context or CMD trait as assessed by a Wilcoxon test. Significance thresholds for unadjusted *p*-values: *p<0.05, **p<0.01, ***p<0.001, and ****p<0.0001. Exact *p*-values for all comparisons are listed in Additional file 4: Table S18.

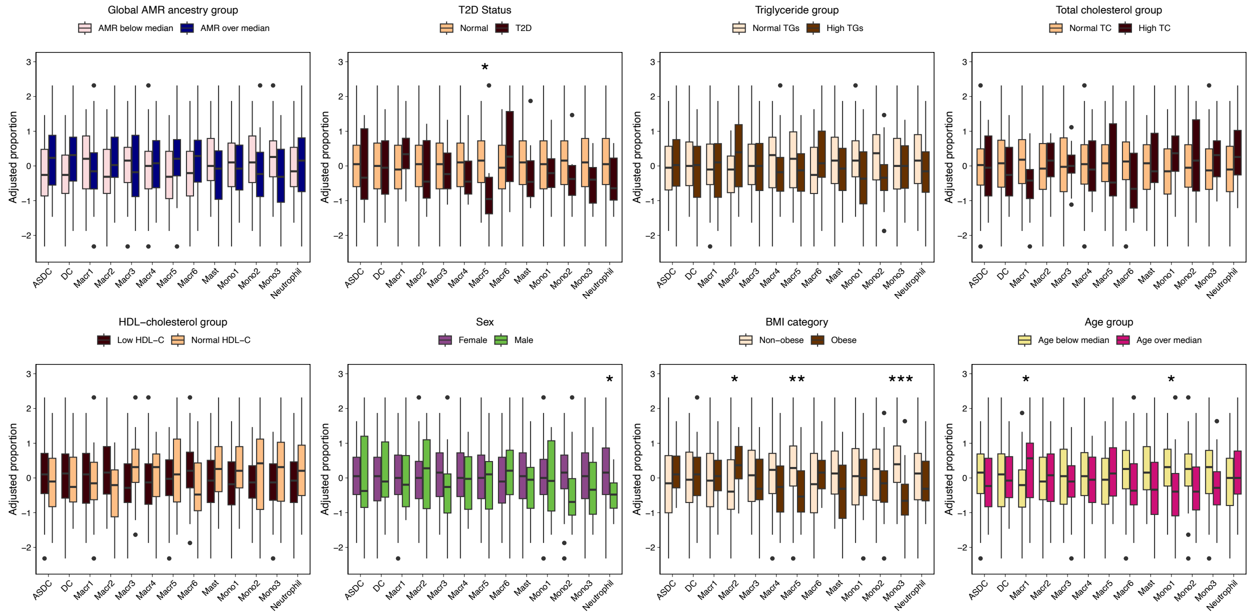

Fig. S22. Comparisons of myeloid subtypes identify a myeloid subtype associated with BMI.

Boxplots compare the myeloid subtype proportions in the SAT snRNA-seq data of the 49 Mexican individuals by sex, BMI, global admixed American (AMR) ancestry, age, serum HDL-cholesterol (HDL-C), serum total cholesterol (TC), serum triglycerides (TGs), and the type 2 diabetes (T2D) status. The following groups were used: males and females; non-obese BMI (BMI<30) and obese (BMI≥30); lower global AMR ancestry (global AMR ancestry≤ median of 0.624) and higher global AMR ancestry (global AMR ancestry>0.624); lower age (age≤ median of 47 years) and higher age (age>47); low HDL-C (HDL-C<40 mg/dL for males, <50mg/dL for females) and normal HDL-C; normal TC (TC<200 mg/dL) and high TC (TC≥200); normal TGs (TGs<150mg/dL) and high TGs (TGs≥150); and T2D cases and controls. Subtype proportions were adjusted for age, global AMR ancestry, sex, and BMI, omitting each covariate when it was the tested outcome. The box limits indicate the first and third quartiles; whiskers of each box, 1.5 × the interquartile range (IQR) from the first and third quartiles; center line, median; and points, outliers. Asterisks denote a significant difference in subtype proportions between the two

groups per context or CMD trait as assessed by a Wilcoxon test. Significance thresholds for unadjusted p-values: *$p<0.05$, **$p<0.01$, ***$p<0.001$, and ****$p<0.0001$. Exact p-values for all comparisons are listed in Additional file 4: Table S18.

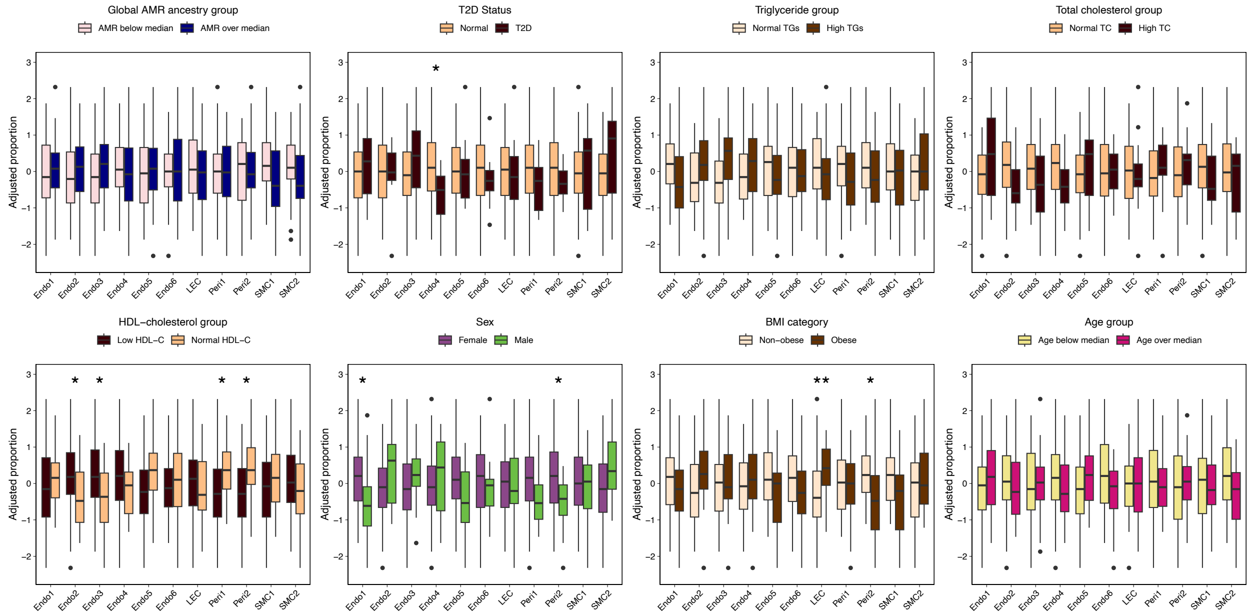

Fig. S23. The vascular subtypes show minor differences in proportions by CMD context and CMD trait.

Boxplots compare the vascular subtype proportions in the SAT snRNA-seq data of the 49 Mexican individuals by sex, BMI, global admixed American (AMR) ancestry, age, serum HDL-cholesterol (HDL-C), serum total cholesterol (TC), serum triglycerides (TGs), and the type 2 diabetes (T2D) status. The following groups were used: males and females; non-obese BMI (BMI<30) and obese (BMI≥30); lower global AMR ancestry (global AMR ancestry≤ median of 0.624) and higher global AMR ancestry (global AMR ancestry>0.624); lower age (age≤ median of 47 years) and higher age (age>47); low HDL-C (HDL-C<40 mg/dL for males, <50mg/dL for females) and normal HDL-C; normal TC (TC<200 mg/dL) and high TC (TC≥200); normal TGs (TGs<150mg/dL) and high TGs (TGs≥150); and T2D cases and controls. Subtype proportions were adjusted for age, global AMR ancestry, sex, and BMI, omitting each covariate when it was the tested outcome. The box limits indicate the first and third quartiles; whiskers of each box, 1.5 × the interquartile range (IQR) from the first and third quartiles; center line, median; and points, outliers. Asterisks denote a significant difference in subtype proportions between the two

groups per context or CMD trait as assessed by a Wilcoxon test. Significance thresholds for unadjusted *p*-values: *$p<0.05$, **$p<0.01$, ***$p<0.001$, and ****$p<0.0001$. Exact *p*-values for all comparisons are listed in Additional file 4: Table S18.

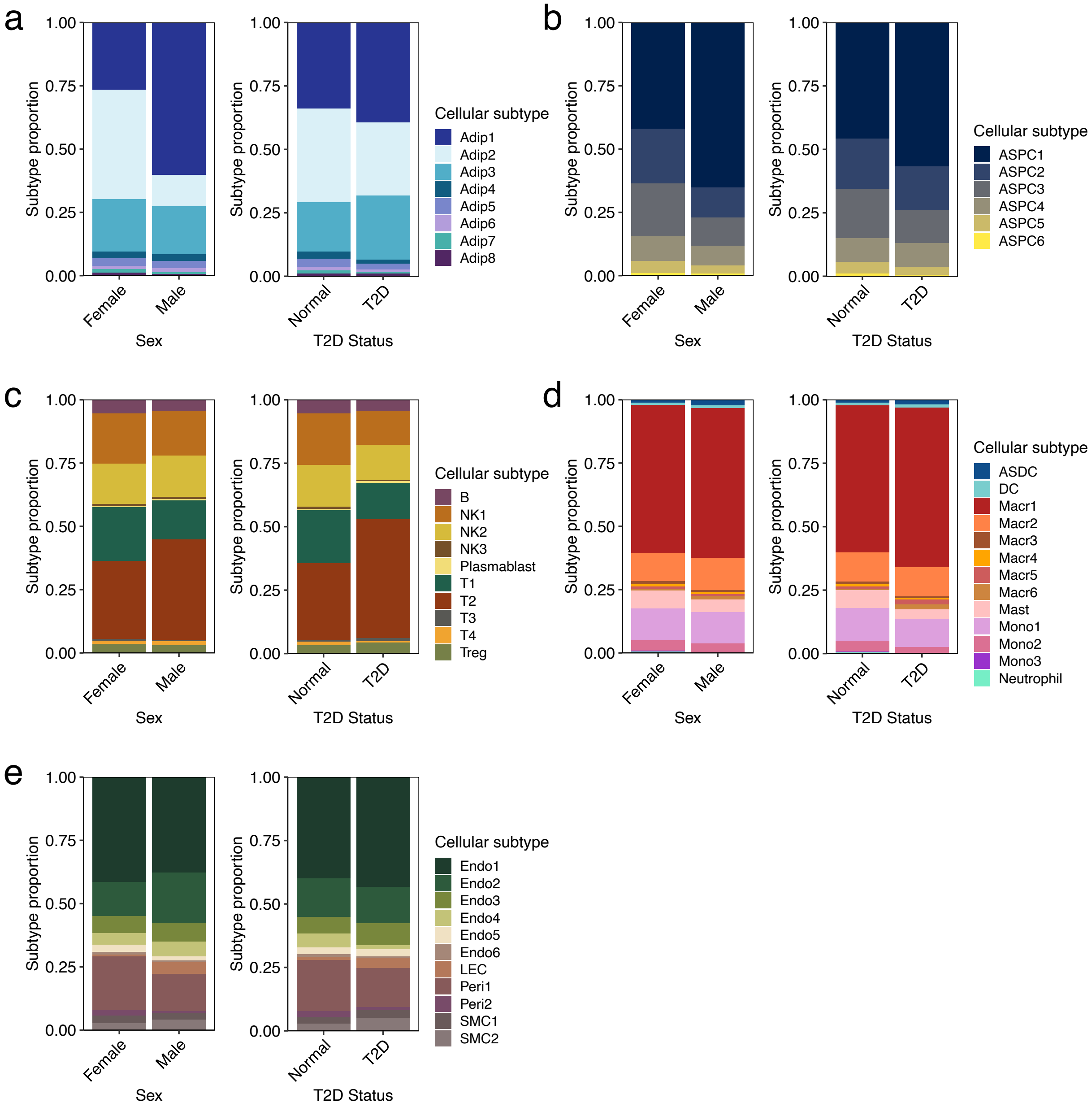

Fig. S24. The proportions of cellular subtypes differ by binary traits (sex and type 2 diabetes (T2D) status) and correlate with continuous contexts and CMD traits.

(a-e) Stacked barplots compare the proportions of a) adipocyte, b) ASPC, c) lymphoid, d) myeloid and e) vascular subtypes within their broad cell-types between females and males (left) and individuals with and without T2D (right). The Wilcoxon *p*-values of these comparisons, after

adjusting for the covariates of age, BMI, global admixed American ancestry, and sex, are provided in Additional file 4: Table S18.

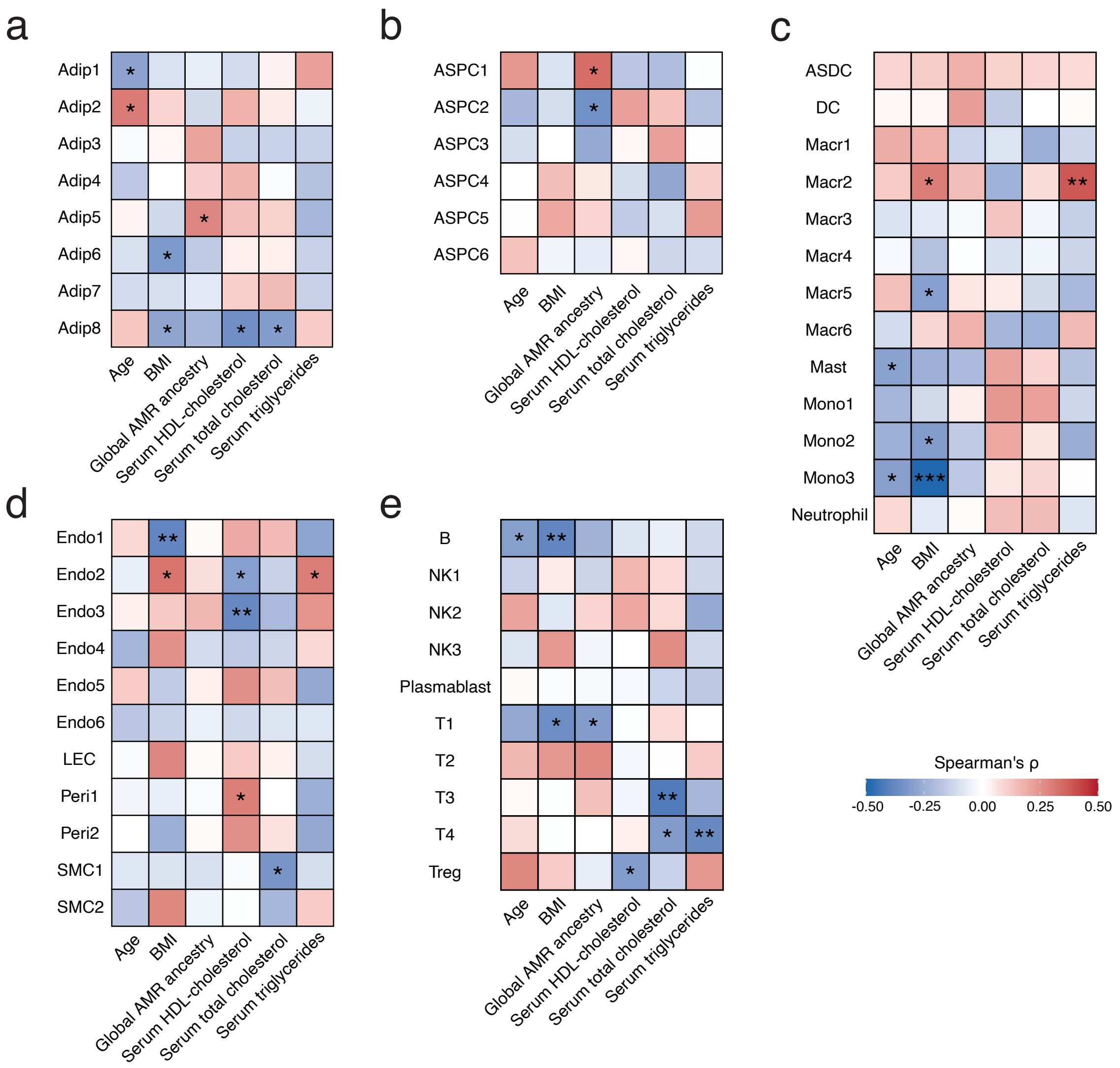

Fig. S25. The proportions of cellular subtypes are correlated with continuous contexts and CMD traits.

(a-e) Heatmaps display the pairwise Spearman's correlations between the continuous outcomes of age, BMI, global admixed American ancestry (AMR), serum high-density lipoprotein (HDL)-cholesterol, serum total cholesterol, and serum triglycerides and proportions of the a) adipocyte subtypes, b) ASPC subtypes, c) myeloid subtypes, d) lymphoid subtypes, and e) vascular subtypes. Each tile is colored by the magnitude of the Spearman's correlation coefficient (ρ). Cell-type proportions were adjusted for age, global AMR ancestry, sex, and BMI, omitting each

covariate when it was the tested outcome. Asterisks denote a nominally significant correlation. Significance thresholds for unadjusted *p*-values: *$p<0.05$, **$p<0.01$, ***$p<0.001$. Exact *p*-values for all correlations are listed in Additional file 4: Table S18.

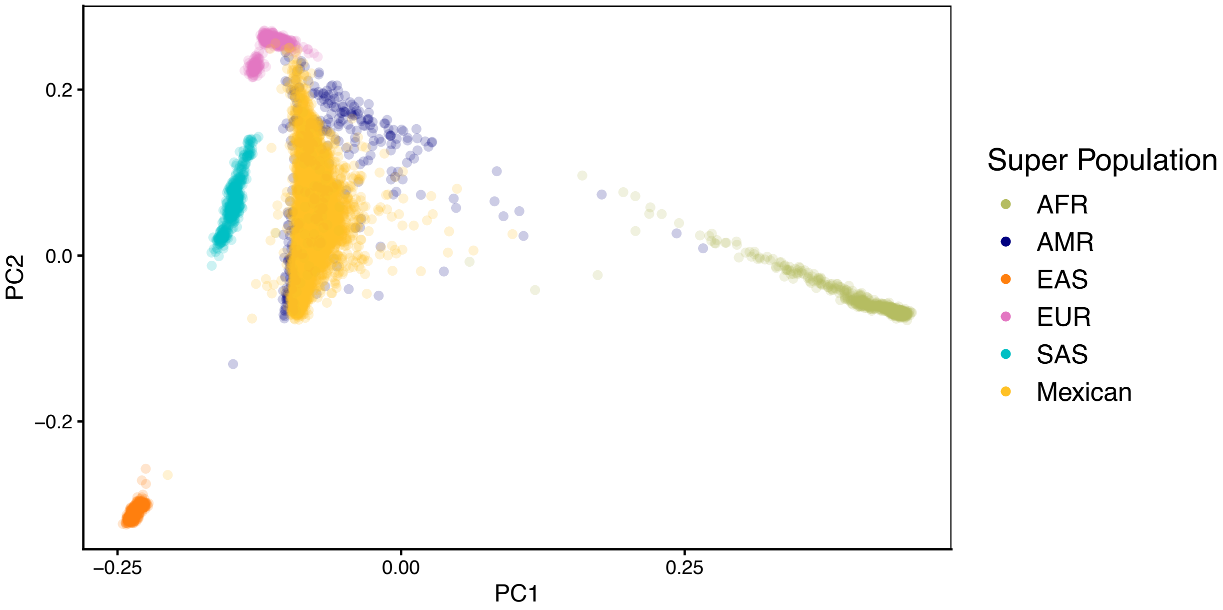

Fig. S26. Principal component analysis of the MetMex cohort on the 1000 Genomes PC space.

The MetMex cohort genotype data (n=8,375) are projected onto the 1000 Genomes principal component space using FlashPCA2[47]. Each dot represents an individual, colored by the population of origin, i.e., one of the superpopulations defined in the 1000 Genomes Project, or Mexican for those in the present study. The admixed American super population is shown as AMR, colored dark blue; European EUR, pink; East Asian EAS, orange; South Asian SAS, teal; African AFR, green; MetMex, yellow.

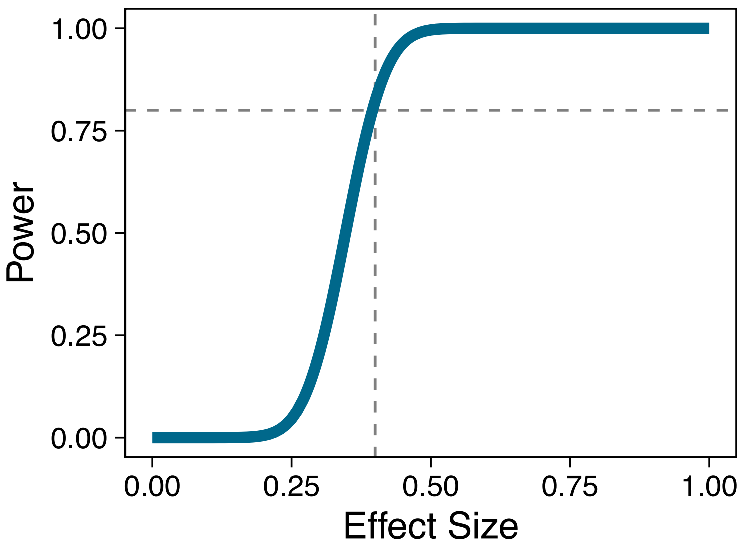

Fig. S27. Power estimation analysis indicates that we have adequate power (>80%) to detect cell-type level *cis*-eQTLs with realistic effect sizes.

Power curve for the cell-type level *cis*-eQTL analysis, derived using the powereQTL tool[97], shows the estimated statistical power to detect *cis*-eQTLs across a range of effect sizes, i.e., the change in gene expression per allele (β), for the minor allele frequency (MAF)>10% cut point (see Methods).

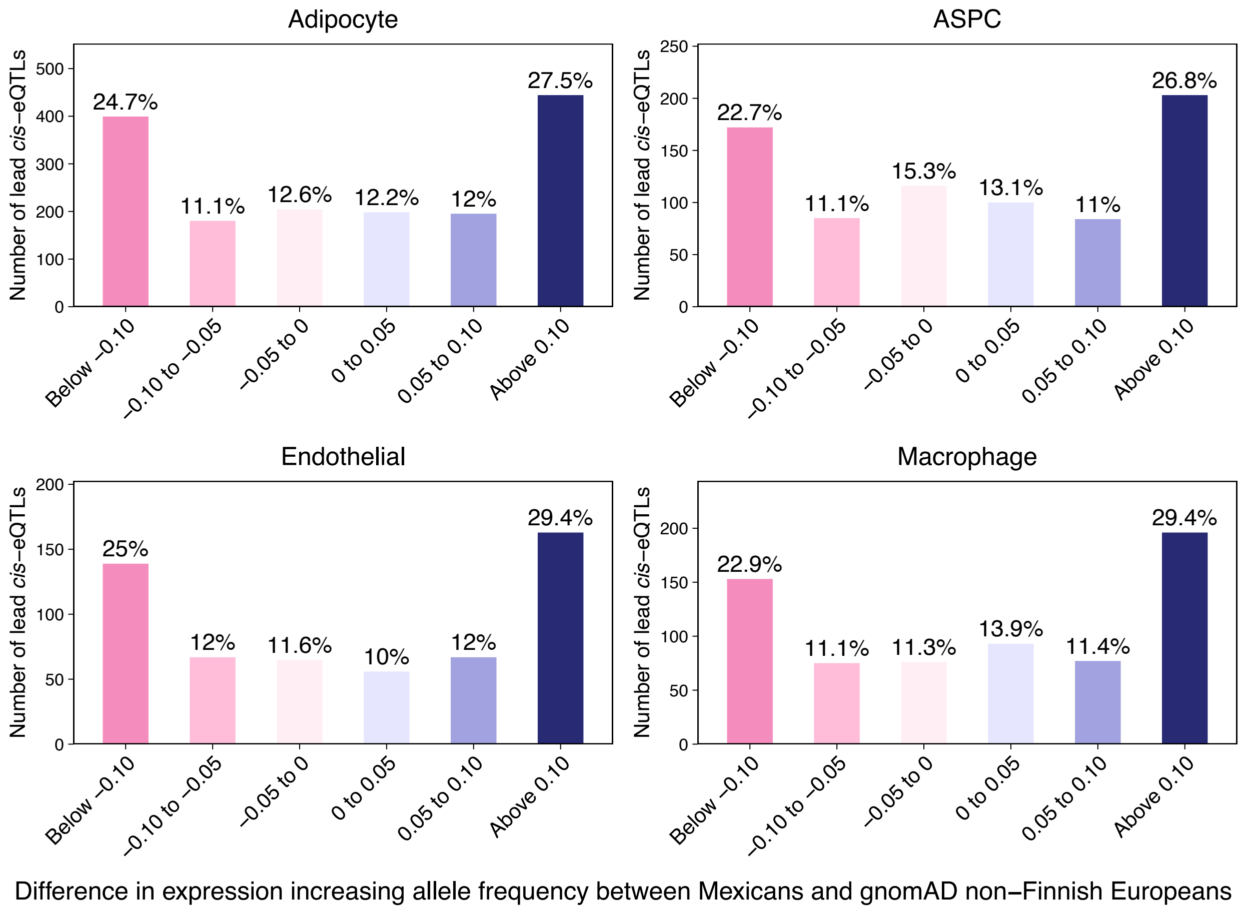

Fig. S28. Cell-type level *cis*-eQTL variants differ in their allele frequencies between the Mexicans and non-Finnish Europeans from gnomAD[129].

Bar plots show the distribution of differences in gene-expression-increasing allele frequencies (AF) between the Mexicans and non-Finnish Europeans for the lead cell-type level *cis*-eQTL variant of each eGene. Variants enriched in the Mexicans are colored blue, while variants enriched in the Europeans are colored pink.

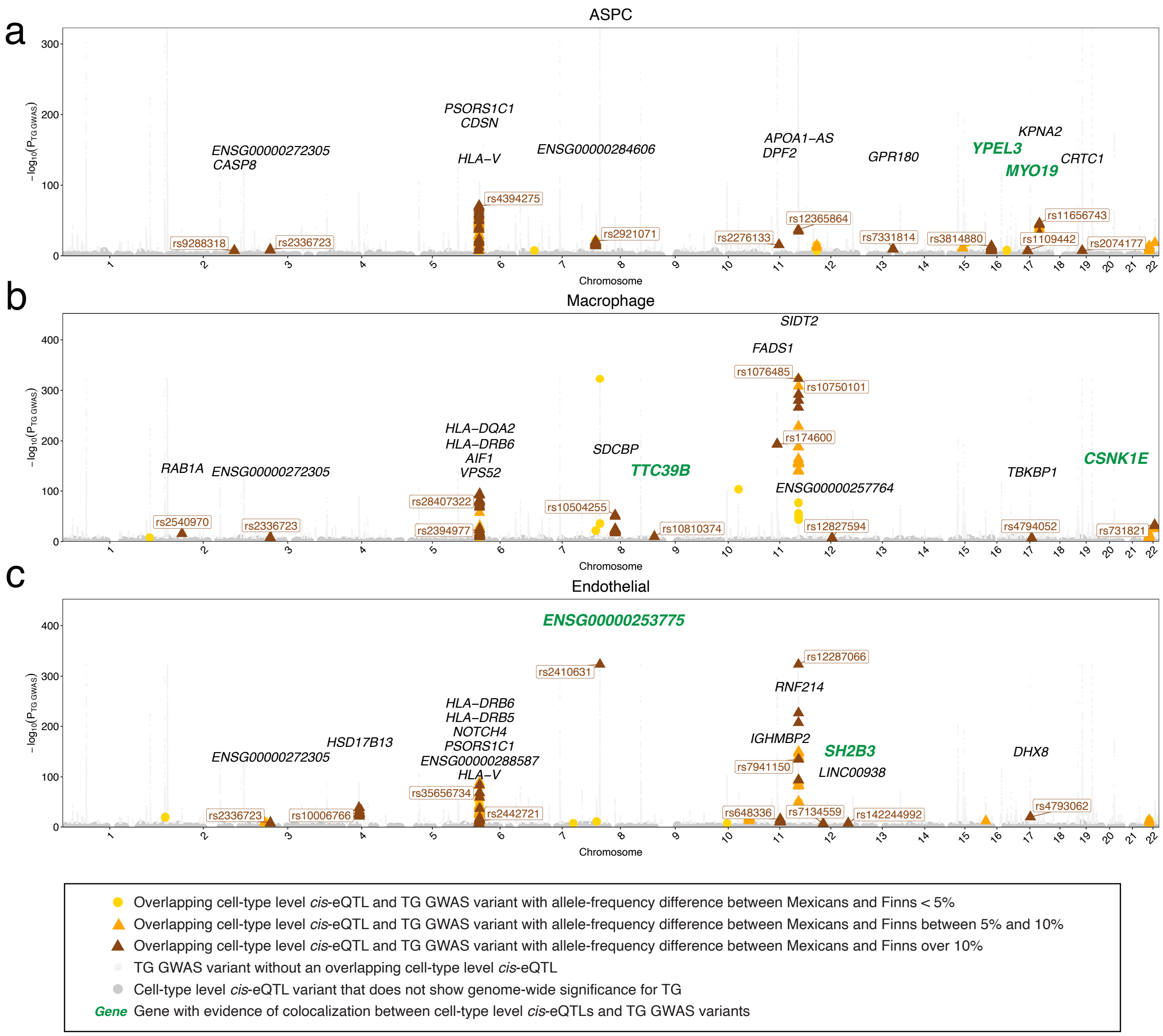

Fig. S29. The ancestry-stratified ASPC, endothelial cell, and macrophage *cis*-eQTL variants overlap and colocalize with GWAS variants for serum triglycerides in the large trans-ancestry GWAS[88].

(a-c) GWAS variants for serum triglycerides (TGs) and a) ASPC, b) macrophage, and c) endothelial cell *cis*-eQTL variants are plotted by chromosomal position against the –log$_{10}$(genomic control-adjusted *p*-value (*p*-value$_{GC}$)) from the GWAS. Orange and brown triangles indicate the significant GWAS variants that overlap with the cell-type level *cis*-eQTL variants and show allele frequency (AF) differences of 5-10% and greater than 10%,

respectively, between the Mexicans and Finns. Gold circles denote the significant GWAS variants that overlap with the cell-type level *cis*-eQTL variants but show no population stratification (AF difference below 5%), while grey points indicate the variants that are either *cis*-eQTL or GWAS variants. We label the most significant GWAS *cis*-eQTL variants per gene that show large population stratifications with their corresponding genes, as well as the genes with significant colocalizations (PPH_4>0.5).

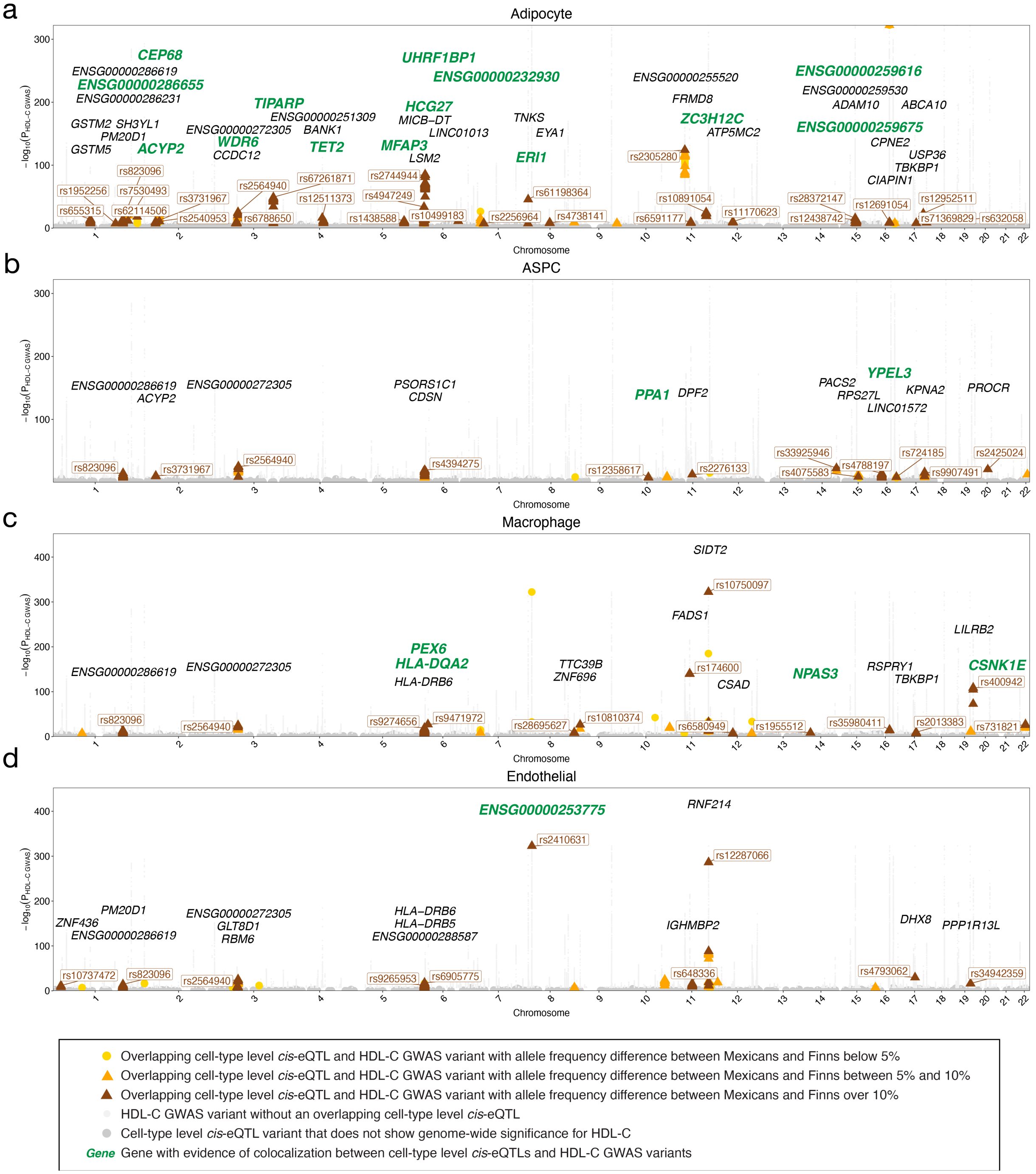

Fig. S30. The ancestry-stratified cell-type level *cis*-eQTL variants overlap and colocalize with GWAS variants for serum HDL-cholesterol in the large trans-ancestry GWAS[88]. **(a-d)** GWAS variants for serum high-density lipoprotein cholesterol (HDL-C) and a) adipocyte, b) ASPC, c) macrophage, and d) endothelial cell *cis*-eQTL variants are both plotted by

chromosomal position against the $-\log_{10}$(genomic control-adjusted *p*-value (*p*-value$_{GC}$)) for the GWAS. Orange and brown triangles indicate the significant GWAS variants that overlap with the cell-type level *cis*-eQTL variants and show allele frequency (AF) differences of 5-10% and greater than 10%, respectively, between the Mexicans and Finns. Gold circles denote the significant GWAS variants that overlap with the cell-type level *cis*-eQTL variants but show no population stratification (AF difference below 5%), while grey points indicate the variants that are either *cis*-eQTL or GWAS variants. We label the most significant GWAS *cis*-eQTL variants per gene that show large population stratifications with their corresponding genes, as well as the genes with significant colocalizations ($PPH_4>0.5$).

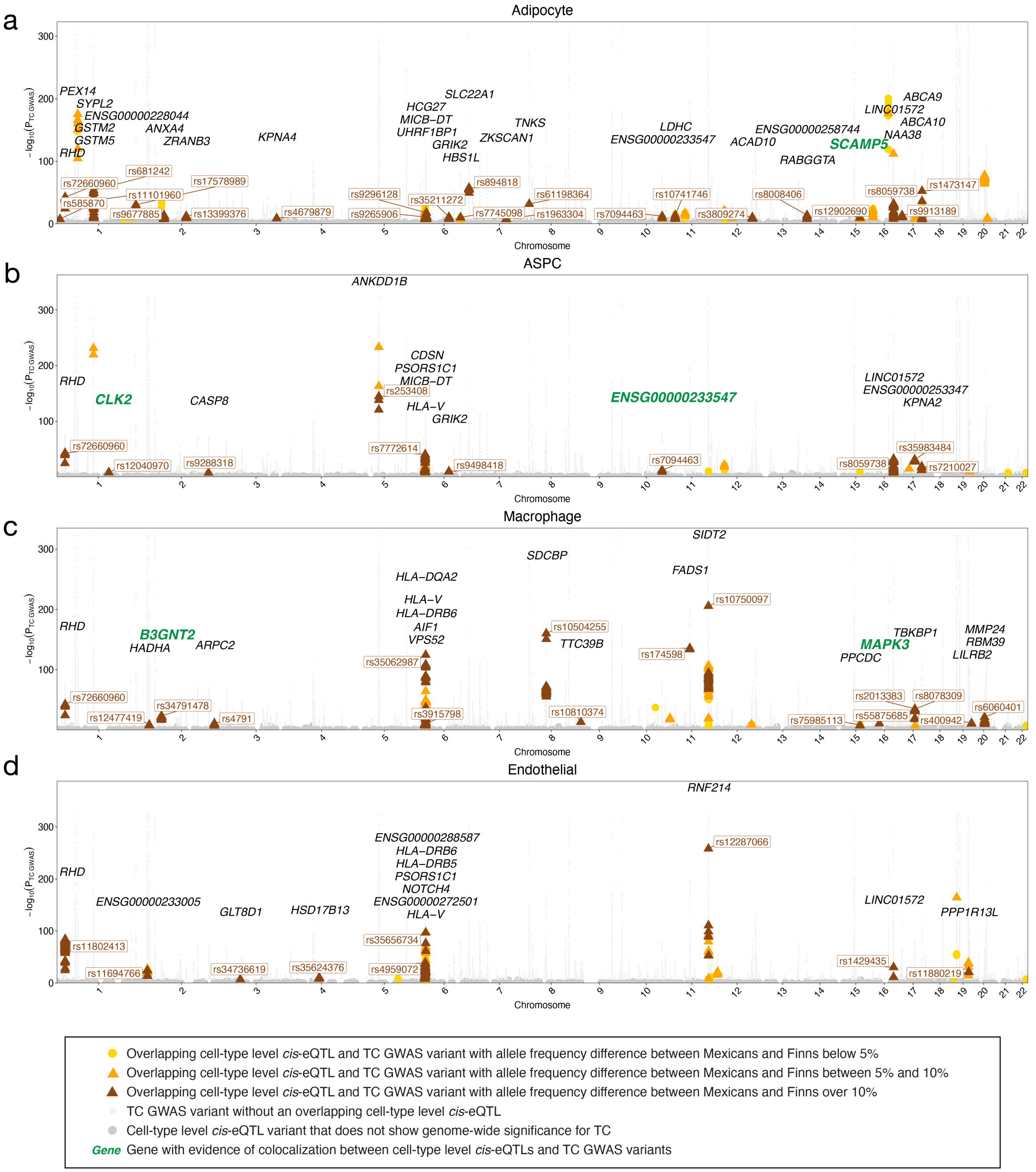

Fig. S31. The ancestry-stratified cell-type level *cis*-eQTL variants overlap and colocalize with GWAS variants for serum total cholesterol in the large trans-ancestry GWAS[88]. (a-d) GWAS variants for serum total cholesterol (TC) and a) adipocyte, b) ASPC, c) macrophage, and d) endothelial cell *cis*-eQTL variants are both plotted by chromosomal position against the –log10(genomic control-adjusted *p*-value (*p*-value$_{GC}$)) for the GWAS. Orange and

brown triangles indicate the significant GWAS variants that overlap with the cell-type level *cis*-eQTL variants and show allele frequency (AF) differences of 5-10% and greater than 10%, respectively, between the Mexicans and Finns. Gold circles denote the significant GWAS variants that overlap with the cell-type level *cis*-eQTL variants but show no population stratification (AF difference below 5%), while grey points indicate the variants that are either *cis*-eQTL or GWAS variants. We label the most significant GWAS *cis*-eQTL variants per gene that show large population stratifications with their corresponding genes, as well as the genes with significant colocalizations ($PPH_4>0.5$).

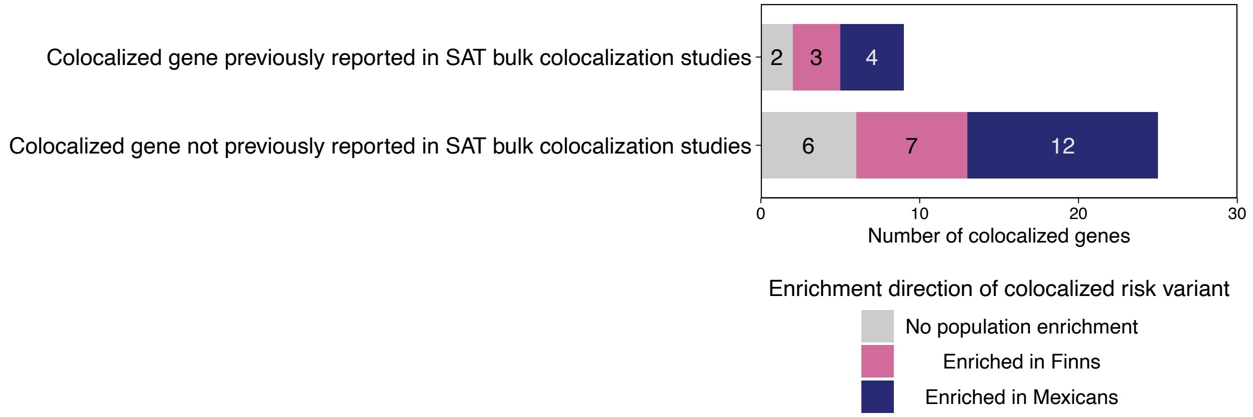

Fig. S32. Of the colocalized genes, 25 genes have not been reported in previous SAT bulk colocalization studies[101], including 12 genes regulated by Mexican-enriched and 7 genes regulated by European enriched colocalized risk variants.

Bar plots depict the number of colocalized genes that have been or have not been reported in previous SAT bulk colocalization studies across their respective colocalized GWAS outcomes. Each bar is proportionally filled and labelled to indicate the number of genes, where the colocalized risk variant is a *cis*-eQTL variant (or a tight LD proxy (r^2>0.8)) enriched in Finns, i.e., has a risk allele frequency difference over 10% higher in Finns; is a *cis*-eQTL variant (or a tight LD proxy) enriched in Mexicans; or is not a population-enriched *cis*-eQTL variant (or tight LD proxy).
